# Wholly *Rickettsia*! Reconstructed Metabolic Profile of the Quintessential Bacterial Parasite of Eukaryotic Cells

**DOI:** 10.1128/mBio.00859-17

**Published:** 2017-09-26

**Authors:** Timothy P. Driscoll, Victoria I. Verhoeve, Mark L. Guillotte, Stephanie S. Lehman, Sherri A. Rennoll, Magda Beier-Sexton, M. Sayeedur Rahman, Abdu F. Azad, Joseph J. Gillespie

**Affiliations:** a Department of Biology, West Virginia University, Morgantown, West Virginia, USA; b Department of Microbiology and Immunology, University of Maryland School of Medicine, Baltimore, Maryland, USA; Ohio State University

**Keywords:** *Rickettsia*, evolution, host-parasite relationship, host-pathogen interactions, intracellular parasites, metabolic modeling, phylogenetic analysis, phylogenomics

## Abstract

Reductive genome evolution has purged many metabolic pathways from obligate intracellular *Rickettsia* (*Alphaproteobacteria*; *Rickettsiaceae*). While some aspects of host-dependent rickettsial metabolism have been characterized, the array of host-acquired metabolites and their cognate transporters remains unknown. This dearth of information has thwarted efforts to obtain an axenic *Rickettsia* culture, a major impediment to conventional genetic approaches. Using phylogenomics and computational pathway analysis, we reconstructed the *Rickettsia* metabolic and transport network, identifying 51 host-acquired metabolites (only 21 previously characterized) needed to compensate for degraded biosynthesis pathways. In the absence of glycolysis and the pentose phosphate pathway, cell envelope glycoconjugates are synthesized from three imported host sugars, with a range of additional host-acquired metabolites fueling the tricarboxylic acid cycle. Fatty acid and glycerophospholipid pathways also initiate from host precursors, and import of both isoprenes and terpenoids is required for the synthesis of ubiquinone and the lipid carrier of lipid I and O-antigen. Unlike metabolite-provisioning bacterial symbionts of arthropods, rickettsiae cannot synthesize B vitamins or most other cofactors, accentuating their parasitic nature. Six biosynthesis pathways contain holes (missing enzymes); similar patterns in taxonomically diverse bacteria suggest alternative enzymes that await discovery. A paucity of characterized and predicted transporters emphasizes the knowledge gap concerning how rickettsiae import host metabolites, some of which are large and not known to be transported by bacteria. Collectively, our reconstructed metabolic network offers clues to how rickettsiae hijack host metabolic pathways. This blueprint for growth determinants is an important step toward the design of axenic media to rescue rickettsiae from the eukaryotic cell.

## IMPORTANCE

A hallmark of obligate intracellular bacteria is the tradeoff of metabolic genes for the ability to acquire host metabolites. For species of *Rickettsia*, arthropod-borne parasites with the potential to cause serious human disease, the range of pilfered host metabolites is unknown. This information is critical for dissociating rickettsiae from eukaryotic cells to facilitate rickettsial genetic manipulation. In this study, we reconstructed the *Rickettsia* metabolic network and identified 51 host metabolites required to compensate patchwork *Rickettsia* biosynthesis pathways. Remarkably, some metabolites are not known to be transported by any bacteria, and overall, few cognate transporters were identified. Several pathways contain missing enzymes, yet similar pathways in unrelated bacteria indicate convergence and possible novel enzymes awaiting characterization. Our work illuminates the parasitic nature by which rickettsiae hijack host metabolism to counterbalance numerous disintegrated biosynthesis pathways that have arisen through evolution within the eukaryotic cell. This metabolic blueprint reveals what a *Rickettsia* axenic medium might entail.

## INTRODUCTION

The members of the order *Rickettsiales* (*Alphaproteobacteria*) are obligate intracellular bacteria found in species across nearly every major lineage of *Eukaryota* (1). Robust phylogeny estimation places the families *Rickettsiaceae*, *Anaplasmataceae*, and *Midichloriaceae* as sisters to the mitochondrial progenitor (2), with the basal rickettsial lineage now recognized as a new order (*Holosporales* ord. nov.) (3). Rickettsial species of medical and agricultural significance are almost exclusively found in the families *Rickettsiaceae* and *Anaplasmataceae* (4), though we lack information regarding the impact on host fitness of many of the formally recognized species and most of the putative species. Despite this, all members of the order *Rickettsiales* can be considered metabolic parasites, as tremendous reductive genome evolution has resulted in a seemingly inextricable metabolic dependence on the eukaryotic cell (5).

Members of family *Rickettsiaceae* (e.g., *Rickettsia* and *Orientia* species) are unique among the members of the order *Rickettsiales* in lysing the host phagocytic vacuole and residing primarily in the host cytosol (6). This lifestyle affords access to a broad range of host metabolites, some of which may not be available to vacuole-enclosed bacteria. Indeed, members of the family *Rickettsiaceae* have a more diminished metabolic capability than other members of the order *Rickettsiales* (7–9). Unlike other notable cytosolic pathogens (e.g., *Listeria monocytogenes*, *Shigella flexneri*, *Francisella tularensis*), members of the family *Rickettsiaceae* require the host cell for replication and cannot persist extracellularly (10). For the genus *Rickettsia*, which contains dozens of formally recognized species ranging from nonpathogens to serious human pathogens (11), the doubling time is typically 8 to 12 h (12). It has been suggested that slow rickettsial growth correlates with a large array of transport systems and limiting metabolite availability in the host cytosol (13), a phenomenon better realized when considering the large numbers of rickettsiae capable of occupying a single cell.

The metabolic deficiency of *Rickettsia* spp. is well established, with studies from the pregenomics era illuminating a limited oxidative metabolism (14). Despite evidence of a functional pyruvate dehydrogenase complex (PDC) and tricarboxylic acid (TCA) cycle (15–20), glycolysis/gluconeogenesis enzymatic activities were undetectable (15, 17, 21). Import of host Glu (22, 23) and Gln (24) was determined, with a metabolic flux among Glu, Gln, and 2-oxaloglutarate indicating the importance of Glu as an energy source (15, 20, 23, 25, 26). Glu oxidation was shown to drive electron transport coupled to oxidative phosphorylation (22, 27–29), with generated ATP facilitating import of Pro (30, 31) and Lys (32, 33). Import of Ser and Gly was demonstrated to be critical for rickettsial growth (34, 35), with Ser-Gly interconversion via serine hydroxymethyltransferase (GlyA) observed (35). These findings, combined with characterized uptake of Met (36, 37), indicated that *Rickettsia* species are likely auxotrophic for the majority of proteogenic amino acids.

Despite generating ATP via Glu oxidation, *Rickettsia* species were shown to possess an ATP/ADP symporter, termed nucleotide translocase (Tlc1), which exchanges host ATP for bacterial ADP without a change in the total adenylate pool (38). Rickettsial membranes were also shown to be permeable to NAD^+^, another host energy source (39). Uptake of UDP-glucose was characterized and suggested to provide the main sugar source for the synthesis of the slime layer, lipopolysaccharide (LPS), and peptidoglycan (PGN) (21). Studies of nucleotide transport and metabolism revealed the import of AMP, GMP, and UMP, indicating that host-acquired ribonucleotides likely serve as building blocks for RNA synthesis and as precursors for deoxyribonucleotide production (40–42). Unlike ATP, the specific transport systems for NAD^+^, UDP-glucose, and nucleoside monophosphates were not identified; however, the emerging consensus was that rickettsiae contain an elaborate assemblage of transport systems for the acquisition of host metabolites (43).

The first reported genome sequence of a *Rickettsia* species, *Rickettsia prowazekii*, confirmed the limited metabolic capacity of rickettsiae, with genes underpinning glycolysis and biosynthesis of pentose phosphates, amino acids, and nucleotides largely absent (44). An abundance of pseudogenes pointed to “reductive genome evolution” (45–47), a characteristic shared by all subsequently sequenced *Rickettsia* genomes (1). Phylogenomics studies observed additional depleted metabolic pathways, particularly those for B vitamins, many cofactors, and pentose phosphates (48–50). Efforts in the postgenomics era identified rickettsial import of the glycerophospholipid precursors dihydroxyacetone phosphate (DHAP) and *sn*-glycerol 3-phosphate (G3P) (51, 52) and also the cosubstrate *S*-adenosylmethionine (SAM), for which a novel transporter (EamA) was characterized (53). Additionally, substrate ranges of four Tlc1 paralogs (Tlc2 to Tlc5) were investigated, revealing that none function in energy exchange, yet two import host ribonucleotides (Tlc4 [CTP, UTP, and GDP] and Tlc5 [GTP and GDP]) (54). Collectively, these genome-driven studies further defined rickettsial metabolic parasitism, illustrating the dependence of rickettsiae on host metabolites.

Although they possess limited metabolic activity, isolated rickettsiae are unable to grow extracellularly. Despite tremendous efforts in rickettsial genetic manipulation over the last 2 decades (55), the lack of an axenic medium continues to impede progress. Knowledge of the range of essential metabolites would greatly facilitate the establishment of an axenic culture, as has previously been shown for another obligate intracellular bacterium, *Coxiella burnetii* (56, 57). In the present study, we used 84 rickettsial genomes to reconstruct the *Rickettsia* metabolic and associated transport networks. Our results indicate that 51 host metabolites are required to compensate for the patchwork *Rickettsia* metabolic pathways and that the majority of cognate transporters for these metabolites are unknown. Our analysis also reveals several pathways that contain isolated holes (missing enzymes); similar patterns across a range of taxonomically diverse taxa imply alternative metabolic strategies shared by divergent bacteria. The comprehensive metabolic blueprint developed here illuminates what a successful *Rickettsia* axenic medium might entail. Furthermore, our work elucidates the parasitic nature by which rickettsiae pilfer host metabolites to counterbalance the many biosynthesis pathways that have disintegrated through evolution within the eukaryotic cell.

## RESULTS AND DISCUSSION

Our metabolic network reconstruction entailed the analysis of 84 *Rickettsia* genomes, with 74 genomes comprising the three well-established rickettsial lineages (48, 58, 59): the transitional group (*n* = 10), the typhus group (*n* = 15), and the spotted fever group (*n* = 49). Ten additional genomes are from basal lineages that do not form a monophyletic group. When considering only closed genomes (*n* = 54), *Rickettsia* genomes range in size from 1.5 Mb (*Rickettsia bellii* strain RML369-C, with 1,429 coding sequences [CDS]) to 1.1 Mb (*Rickettsia typhi* strain Wilmington, with 838 CDS), with a core genome including 621 protein-encoding genes. Variation in accessory genomes is dominated by pseudogenization events, but for some species (e.g., *Rickettsia buchneri* [60] and *Rickettsia peacockii* [61]), it can also consist of extensive mobile genetic elements. The genes encoding components of the metabolic and transport networks described below make up 25% of the core genome, indicating a highly conserved strategy for parasitizing the eukaryotic cytoplasm.

### Synthesis of cell envelope glycans requires host precursors.

**FIG 1:**
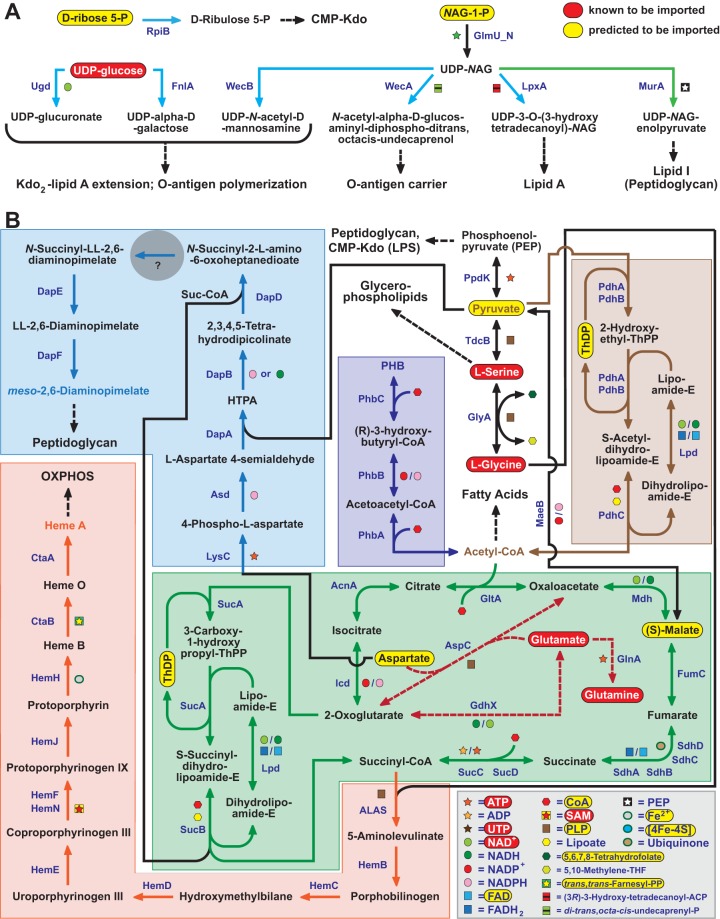
*Rickettsia* species synthesize cell envelope glycoconjugates from imported host sugars and fuel the TCA cycle with a range of host-acquired metabolites. (A) Previously shown to be imported, UDP-glucose is predicted to yield UDP-glucuronate and UDP-α-d-galactose, sugars likely to be used in LPS synthesis (light blue pathway lines). Synthesis of UDP-*N*-acetyl-d-mannosamine, another sugar likely incorporated into LPS, as well as pathways for O-antigen, lipid A, and lipid I of PGN (green pathway line), initiates with UDP-NAG. Without glycolysis enzymes, rickettsiae are predicted to import host NAG-1-P and convert this charged sugar to UDP-NAG via the uridyltransferase GlmU. d-Ribose 5-P, which is required to initiate CMP-Kdo synthesis, is also predicted to be imported from the host, provided that *Rickettsia* species lack enzymes of the pentose phosphate pathway. (B) *Rickettsia* species must acquire pyruvate for generation of PEP and acetyl-CoA. Pyruvate interconversions with Ser, Gly (via Ser), and malate are likely mediated by additional import of these molecules, ensuring that enough pyruvate enters the PDC to yield acetyl-CoA (brown). Aside from entering the TCA cycle (green), acetyl-CoA is also used in fatty acid biosynthesis and production of PHB (dark blue), a storage molecule that is metabolized when host energy sources are unavailable. Imported malate, glutamine (Gln), and glutamate (Glu) likely regulate the flow of acetyl-CoA into the TCA cycle, with Gln/Glu interconversions with 2-oxaloglutarate and oxaloacetate providing additional energy (dashed burgundy pathway lines). Generated aspartate is essential for initiation of the synthesis of DAP, which is used in PGN biosynthesis (light blue), a pathway nearly conserved except for a central hole (gray circle). DAP synthesis also requires generated succinyl-CoA, which is also used to synthesize porphyrins (orange). HTPA, (2S,4S)-4-hydroxy-2,3,4,5-tetrahydrodipicolinate.

Without the products of glycolysis and the pentose phosphate pathway, rickettsiae must acquire host metabolites for the synthesis of PGN and LPS (Fig. 1A; see Fig. S1A in the supplemental material). The first amino sugar able to be synthesized in the stem pathway leading to PGN/LPS biosynthesis is UDP-*N*-acetyl-α-d-glucosamine (UDP-NAG), which is typically synthesized from glucosamine-1-P (GlcN-1-P) in bacteria by the bifunctional protein GlmU. The GlmU C-terminal acetyltransferase domain generates *N*-acetylglucosamine-1-P (NAG-1-P) from GlcN-1-P, while the N-terminal uridyltransferase domain converts NAG-1-P to UDP-NAG (62) (see Fig. S1B). However, the predominant eukaryotic pathway (including vertebrates and arthropods) generates NAG-1-P from NAG-6-P, not GlcN-1-P (see Fig. S1C), suggesting that rickettsiae acquire NAG-1-P from the host. Remarkably, the rickettsial GlmU proteins lack the entire C-terminal acetyltransferase domain (see Fig. S1D) and thus are streamlined for the conversion of host-acquired NAG-1-P to UDP-NAG. Generated UDP-NAG then enters pathways for PGN and LPS (both lipid A and O-antigen) biosynthesis (see Fig. S1E; Fig. S1F, respectively).

10.1128/mBio.00859-17.1 FIG S1  *Rickettsia* species import six host metabolites that are required for the synthesis of cell envelope glycoconjugates and glycerophospholipids. Download FIG S1, PDF file, 2 MB. Copyright © 2017 Driscoll et al. 2017 Driscoll et al. This content is distributed under the terms of the Creative Commons Attribution 4.0 International license.

UDP-NAG is also a source for the generation of UDP-*N*-acetyl-d-mannosamine, one of three sugars predicted to make up the precursors for extension of the Kdo_2_ (two 3-deoxy-d-manno-octulosonic acid residues)-lipid A acceptor and O-antigen polymerization (see Fig. S1F). The other two sugars, UDP-glucuronate and UDP-α-d-galactose, must derive from host-acquired UDP-glucose (21), which is a highly abundant amino sugar in vertebrate cells (63). The complete synthesis of lipid A also requires the import of host d-ribose 5-P, an essential precursor of CMP-Kdo synthesis. CMP-Kdo, which is added to lipid IV(A) in sequential steps (64), is typically synthesized from d-ribose 5-P using five enzymes (see Fig. S1F). The rickettsial CMP-Kdo biosynthesis pathway is complete, except for a single pathway hole (see Materials and Methods for a more complete definition of a pathway hole); it lacks the phosphatase KdsC (see Fig. S1G to I). Similarly, the rickettsial biosynthesis of diaminopimelate (DAP), a component of the PGN stem peptide, also contains a pathway hole; it lacks the *N*-succinyldiaminopimelate aminotransferase DapC or related enzymes (Fig. 1B; see Fig. S2). Otherwise, pathways for PGN and LPS biosynthesis are highly conserved in rickettsial genomes, indicating that acquisition of host NAG-1-P, UDP-glucose, and d-ribose 5-P suffices for initiation of the metabolism of cell envelope glycans (Fig. 1A).

10.1128/mBio.00859-17.2 FIG S2  The *Rickettsia* DAP biosynthesis pathway contains a hole for the conversion of *N*-succinyl-l-2-amino-6-oxopimelate to *N*-succinyl-ll-2,6-DAP. Download FIG S2, PDF file, 0.4 MB. Copyright © 2017 Driscoll et al. 2017 Driscoll et al. This content is distributed under the terms of the Creative Commons Attribution 4.0 International license.

10.1128/mBio.00859-17.3 FIG S3  The *Rickettsia* glycerophospholipid biosynthesis pathway contains a hole for the conversion of G3P to LPA. Download FIG S3, PDF file, 0.4 MB. Copyright © 2017 Driscoll et al. 2017 Driscoll et al. This content is distributed under the terms of the Creative Commons Attribution 4.0 International license.

### A range of imported metabolites fuels the TCA cycle.

The TCA cycle is a key metabolic pathway that uses the oxidation of acetyl coenzyme A (acetyl-CoA) to generate energy, as well as important substrates for other biosynthetic processes. The principal source of acetyl-CoA is pyruvate, generated by the breakdown of sugars via glycolysis—a pathway that rickettsiae do not possess. Rickettsiae must obtain pyruvate for a variety of reasons, including use in DAP biosynthesis (leading to PGN) and for the generation of phosphoenolpyruvate (PEP), a cofactor in the biosynthesis of both PGN and LPS (Fig. 1B). Pyruvate interconversions with PEP, Ser, Gly (via Ser), and also malate from the TCA cycle indicate an intricate network for the regulation of pyruvate entry into the PDC, which generates acetyl-CoA. Rickettsiae have been shown previously to uptake Ser and Gly (34, 35), and our analysis suggests the need, and potentially the capacity, to import pyruvate and/or malate from host cells as well: all 84 rickettsial genomes encode Auxin Efflux Carrier (AEC) transporters (YfdV), which are known to transport small dicarboxylates including malate (65, 66), and almost all encode on an adjacent locus the gene for MaeB, which interconverts malate and pyruvate.

PDC-generated acetyl-CoA is used primarily for the TCA cycle and fatty acid biosynthesis (Fig. 1B). A pathway for the conversion of acetyl-CoA to polyhydroxybutyrate (PHB), a storage molecule that is metabolized when host energy sources are unavailable (67), is also found in most rickettsial genomes, though it is noticeably less conserved in spotted fever group species (see Fig. S10). This is consistent with the variability of observed intracytoplasmic vacuoles across different *Rickettsia* species (68), as these distinct structures are known to house accumulated polyhydroxyalkanoates such as PHB (69, 70).

Interestingly, a pathway for acetyl-CoA generation from acetate, employing acetate kinase and phosphate acetyltransferase, is highly conserved in typhus and transitional group rickettsiae but diminished in most other rickettsial lineages (data not shown). Like the PDC, this pathway requires CoA, a cofactor that rickettsiae must acquire from the host (discussed below), as well as acetate, which was previously shown to be imported by typhus group rickettsiae (71). Remarkably, only typhus group rickettsiae contain WecH, an enzyme used by *Escherichia coli* to acetylate O-antigen (72), indicating that these rickettsiae alone can incorporate acetate into LPS.

In addition to host-acquired malate and *Rickettsia*-generated acetyl-CoA, imported Gln and Glu also regulate the flow of acetyl-CoA into the TCA cycle (Fig. 1B). The presence in all rickettsiae of three enzymes underpinning these conversions (AspC, GdhX, and GlnA) is consistent with the essentiality of Glu/Gln uptake and the fact that Gln is the most abundant free amino acid in human blood and other tissues (73). Asp generated via these processes is essential for initiation of the synthesis of DAP, which was previously shown to be a component of the rickettsial PGN stem peptide (74). Succinyl-CoA produced by the TCA cycle is also required for DAP synthesis, as well as the synthesis of porphyrins important to electron transport. Like nonphotosynthetic eukaryotes and other members of the class *Alphaproteobacteria*, rickettsieae initiate porphyrin biosynthesis by forming δ-aminolevulinic acid from Gly and succinyl-CoA. Ten conserved enzymes, including the alternative protoporphyrinogen IX oxidase HemJ (75), are used to generate heme A, the prosthetic group of cytochromes associated with cytochrome *c* oxidase of the electron transport chain. Collectively, a wide array of characterized (Ser, Glyc, Glu, Gln) and predicted (pyruvate, malate) host metabolites, as well as numerous host cofactors (see “Rickettsieae cannot synthesize B vitamins and most other cofactors” below) are used by rickettsiae to drive the TCA cycle, which in turn feeds the pathways for the generation of DAP and heme A for PGN and oxidative phosphorylation, respectively.

### Host metabolites and a noncanonical carboxylation complex are required to initiate fatty acid and glycerophospholipid biosynthesis.

**FIG 2:**
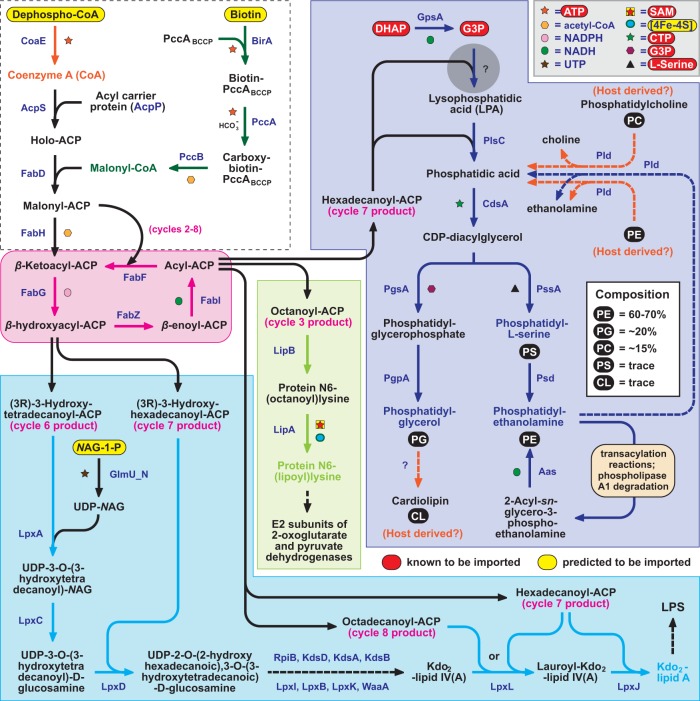
*Rickettsia* species synthesize fatty acids and glycerophospholipids from host precursors. (Dashed box) Predicted imported substrates dephospho-CoA and biotin are required for holo-ACP synthesis and loading of the biotin carboxyl carrier protein, respectively, which collectively lead to the formation of malonyl-ACP. As *Rickettsia* species lack ACC, a conserved PCC complex is predicted to generate malonyl-CoA (green). Type II fatty acid synthesis (pink) is utilized by *Rickettsia* species to generate octanoyl-ACP for lipoate synthesis (light green), β-hydroxyacyl-ACPs (14C and 16C) and acyl-ACPs (16C and 18C) for Kdo_2_-lipid A synthesis (light blue), and hexadecanoyl-ACP for glycerophospholipid synthesis (dark blue). Acyl chain incorporation into lipid A follows the structure deduced for *R. typhi* (171). While both DHAP and G3P are known to be imported from the host (51, 52), *Rickettsia* species lack enzymes to generate LPA via the first incorporation of hexadecanoyl-ACP (the gray circle represents this pathway hole). All enzymes subsequent to this step are highly conserved (see Fig. S10), generating the predominant glycerophospholipids characterized in *Rickettsia* membranes (inset at upper right) (81). Dashed lines illustrate possible Pld-mediated salvage pathways for bacterial PE, as well as host PE and PC (orange). If PC (82) and cardiolipin (81) are incorporated into *Rickettsia* membranes, both must be acquired from the host (orange).

Bacterial fatty acid biosynthesis initiates with malonyl acyl carrier protein (malonyl-ACP), which is formed from holo-ACP and malonyl-CoA (Fig. 2). Rickettsial ACP synthesis requires CoA, which our analysis suggests is likely generated from host-acquired dephospho-CoA, since the CoA synthesis pathway contains only the terminal enzyme (dephospho-CoA kinase). Malonyl-CoA synthesis in bacteria occurs predominantly via the carboxylation of acetyl-CoA by acetyl-CoA carboxylase (ACC), a multisubunit complex consisting of a biotin carboxyl carrier protein (AccB), a biotin carboxylase (AccC), and a carboxyltransferase (AccA/AccD). Rickettsiae lack the genes that encode any of the ACC subunits; however, our analysis has identified all of the functional domains required for the carboxylation of acetyl-CoA in two conserved rickettsial proteins originally annotated as subunits of propionyl-CoA carboxylase (PCC). Rickettsiae have no other enzymes associated with propanoate metabolism; consequently, we propose that *Rickettsia* PCC substitutes for bacterial ACC in the generation of malonyl-CoA. Other lines of evidence support this proposition: (i) PCCs are included within a large family of diverse biotin-dependent carboxylases with highly variable substrate specificities (76); (ii) several archaeal biotin-dependent carboxylases are bifunctional, metabolizing both acetyl-CoA and propionyl-CoA (77, 78); and (iii) *Wolbachia* PCC can complement an *E. coli* ACC mutant (79). Finally, rickettsiae must still import host biotin to initiate acetyl-CoA carboxylation (60), as they are equipped with biotin ligase and the BioY transporter but lack the enzymes for *de novo* biotin synthesis (although see “In light of metabolic parasitism, what, exactly, is a *Rickettsia* endosymbiont?” below for a rare exception).

Major offshoots of the fatty acid cycle used by rickettsiae include octanoyl-ACP for lipoate synthesis, β-hydroxyacyl-ACPs (14C and 16C) and acyl-ACPs (16C and 18C) for Kdo_2_-lipid A synthesis, and hexadecanoyl-ACP for glycerophospholipid synthesis (Fig. 2). For the latter pathway, hexadecanoyl-ACP is used in the conversion of G3P to lysophosphatidic acid (LPA). Previous studies have shown that rickettsiae can either directly import G3P or import DHAP and subsequently generate G3P using GpsA (51, 52). Although our analysis failed to identify a dedicated acyltransferase for the actual generation of LPA (i.e., PlsB or PlsX/Y), enzymes are present for the conversion of LPA to most of the glycerophospholipids previously characterized from rickettsial membranes, including phosphatidylethanolamine (PE), phosphatidylglycerol, and phosphatidyl-l-serine (80, 81). The implications for this pathway hole are discussed in more detail below (“Whole pathways or pathway holes?”).

Despite prior reports of their presence in rickettsial membrane extracts, rickettsiae cannot synthesize cardiolipin or phosphatidylcholine (PC). This indicates that either these previous studies failed to completely eliminate host glycerophospholipids from isolated rickettsial membranes, or host cardiolipin and/or PC are incorporated into rickettsial membranes in trace amounts. Rickettsiae have been shown to hydrolyze PC during infection (82), and all rickettsial genomes do encode phospholipase D (Pld) (83), which was shown to release choline from PC *in vitro* (84). *Rickettsia* genomes also encode one or more choline transferases (LicD) that are known to ligate surface molecules with phosphorylcholine, a process often associated with modulation of host immunity (85). It is conceivable that Pld-mediated host membranolysis provides rickettsiae with choline. In theory, this process could also provide rickettsiae with a direct source of phosphatidic acid for the generation of glycerophospholipids, as would Pld-mediated recycling of PE (Fig. 2), intriguing concepts to consider in light of a missing G3P acyltransferase.

### *Rickettsia* spp. must acquire host isoprenes and terpenoids to synthesize ubiquinone and the lipid carriers for PGN and LPS.

Isoprenes are the general precursors of all terpenoids, a diverse family of compounds involved in a variety of cell functions, including electron transport and membrane synthesis. Bacteria employ the mevalonate or nonmevalonate (MEP/DOXP) pathway to synthesize isoprenes, namely, isopentenyl diphosphate (IPP) and dimethylallyl diphosphate (DMAPP). DMAPP is the source of the dimethylallyl phosphate used in the first step of the ubiquinone (CoQ_8_) pathway. It is also combined with IPP to generate geranyl diphosphate (GPP), which in turn combines with IPP to yield *trans*,*trans*-farnesyl diphosphate (FPP), the precursor of terpenoid backbone synthesis. FPP combined with eight IPP molecules generates di-*trans*,poly-*cis*-undecaprenyl diphosphate (UPP), which is subsequently dephosphorylated to yield di-*trans*,poly-*cis*-undecaprenyl phosphate, the lipid carrier for lipid I of PGN (see Fig. S1E) and O-antigen of LPS (see Fig. S1F). Alternatively, FPP combined with five IPP molecules generates all-*trans*-octaprenyl diphosphate (ODP), a precursor of CoQ_8_ synthesis.

**FIG 3:**
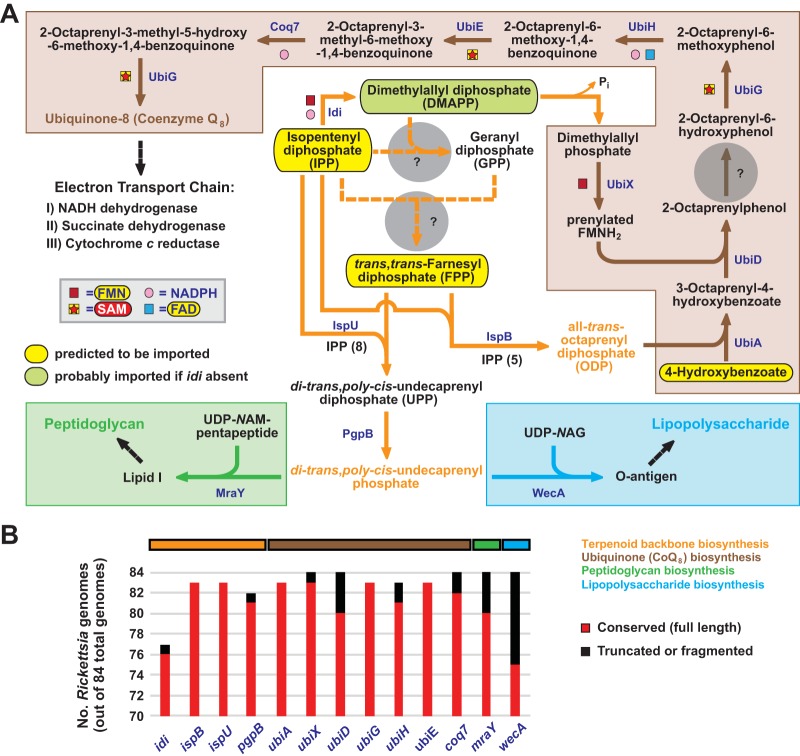
*Rickettsia* species must import host isoprenes and terpenoids for the synthesis of ubiquinone and the lipid carrier of lipid I and O-antigen. (A) In the absence of a mevalonate or MEP/DOXP pathway for terpenoid synthesis, *Rickettsia* species must import IPP from the host. If Idi is present, as it is in some species, DMAPP can be synthesized to provide dimethylallyl phosphate for the ubiquinone (CoQ_8_) pathway. Otherwise, DMAPP must also be imported from the host. Dashed orange lines indicate that no enzymes are present to use IPP and DMAPP for GPP generation, and thus, FPP must also be acquired from the host. Gray circles depict holes in the pathways for the generation of both GPP and FPP. Host-acquired IPP and FPP can then be used by undecaprenyl diphosphate synthase (IspU) and octaprenyl-diphosphate synthase (IspB) to generate terpenoid backbones UPP and ODP, respectively. Via phosphatidylglycerophosphatase B (PgpB), UPP is then converted to di-*trans*,poly-*cis*-undecaprenyl phosphate, the lipid carrier for lipid I (green) and O-antigen (light blue). OPP and PHBA are used by PHBA polyprenyltransferase (UbiA) to initiate CoQ_8_ synthesis (brown). The lack of enzymes to either synthesize chorismate or convert it to PHBA indicates that rickettsiae must import host PHBA, which is an essential host metabolite provided by diet and/or the microbiome. The gray circle indicates a hole (UbiC) in the CoQ_8_ synthesis pathway. Note that all rickettsial genomes encode UbiB, a putative kinase with an unknown role in CoQ_8_ biosynthesis (172). (B) Genes involved in terpenoid backbone and CoQ_8_ biosynthesis are largely conserved. The complete distributions of these genes in 84 *Rickettsia* genomes (see Fig. S4G) indicate that the most basal lineage of spotted fever group rickettsiae (*R. tamurae*, *R. monacensis*, REIP, and *R. buchneri* strains) lacks *idi* and thus must also acquire DMAPP from the host.

Rickettsiae lack enzymes of either the mevalonate or the MEP/DOXP pathway and thus must acquire isoprenes from the host (Fig. 3A). Most rickettsial species can generate DMAPP from IPP using IPP δ-isomerase (Idi); however, a relative lack of *idi* conservation (Fig. 3B) and the similar molecular weights of DMAPP and IPP isomers suggest the possibility, in some species, of a dual transport mechanism for these isoprenes, similar to that observed for DHAP and G3P (52). Our analysis revealed strong conservation of undecaprenyl-PP synthase (IspU) and octaprenyl-PP synthase (IspB), which synthesize UPP and ODP, respectively, but a significant pathway hole (absence of IspA; see Fig. S4A to C) that prevents the synthesis of either GPP or FPP. Consequently, rickettsiae need to import host FPP to synthesize these lipid carriers.

10.1128/mBio.00859-17.4 FIG S4  Pathways for *Rickettsia* terpenoid and ubiquinone biosynthesis contain holes that are not common in other bacterial genomes. Download FIG S4, PDF file, 1.4 MB. Copyright © 2017 Driscoll et al. 2017 Driscoll et al. This content is distributed under the terms of the Creative Commons Attribution 4.0 International license.

Synthesis of CoQ_8_ begins with the conversion of ODP and 4-hydroxybenzoate (PHBA) into 3-octaprenyl-PHBA, a reaction carried out by PHBA polyprenyltransferase (UbiA). Many bacteria synthesize PHBA from chorismate, a precursor of aromatic amino acid synthesis. Although they possess UbiA, rickettsiae lack the enzymes to either synthesize chorismate or convert it to PHBA, indicating that PHBA must be imported from the host (Fig. 3A). The remaining steps in CoQ_8_ synthesis are fairly well conserved, with the exception of a pathway hole at the hydroxylation of 2-octaprenylphenol (OPP) (see Fig. S4D to F). In *E. coli*, this reaction is catalyzed by the dedicated monooxygenase UbiI, while a Δ*ubiI* mutant strain produces a low level of CoQ_8_ and a compound atypical of the CoQ_8_ pathway (86). A different enzyme is responsible for C-5 hydroxylation of OPP under anaerobic conditions (86), however, indicating that alternative ways to carry out this reaction exist. Taken together, the conservation of the CoQ_8_ pathway (Fig. 3B; see Fig. S4G), the import of PHBA, and the synthesis of ODP from host-acquired isoprenes and terpenoids indicate that rickettsiae can effectively supply the electron transport chain with canonical CoQ_8_.

### Rickettsiae cannot synthesize B vitamins and most other cofactors.

In contrast to the conserved pathways for the synthesis of porphyrins, lipoate, and CoQ_8_ by rickettsiae, pathways for the synthesis of B vitamins and other cofactors are incomplete or entirely lacking. Our analysis indicates that at least 11 such molecules must be acquired directly from the host—and possibly more when derivatives are considered—on the basis of the pathways that do function in rickettsiae. Thiamine cannot be synthesized, and rickettsiae do not encode any enzymes that generate thiamine’s five phosphate derivatives; at a minimum, rickettsiae must import thiamine diphosphate (ThDP), as this cofactor is essential for the PDC and the oxoglutarate dehydrogenase complex of the TCA cycle (Fig. 1B). No enzymes are present for the generation of riboflavin from GTP and ribulose-5P or the conversion of riboflavin to flavin mononucleotide (FMN); consequently, rickettsiae must acquire FMN from the host. Flavin adenine dinucleotide (FAD) must also be imported, given that riboflavin kinase/FAD synthetase, which converts FMN to FAD, is absent. Pyridoxal phosphate (PLP) is another B vitamin that rickettsiae cannot synthesize, and given that animals are auxotrophic for PLP, rickettsiae must compete with the host for this substrate. This also applies to biotin and glutathione: rickettsiae contain the enzymes that ligate these substrates to their cellular targets—bifunctional ligase/repressor (BirA) and glutathione *S*-transferase (GstA), respectively—but not the capacity to synthesize the cofactors.

Rickettsiae can synthesize several other B vitamins and cofactors; however, even these pathways are dependent on precursors that they obtain from the host. The synthesis of pantothenate, the precursor of CoA, has likely been replaced by the ability to generate CoA from host-acquired dephospho-CoA as discussed above. Imported NAD^+^ (39) serves as a source for the generation of NADP^+^ via inorganic polyphosphate/ATP-NAD^+^ kinase, with NAD(P)^+^ transhydrogenase providing NADH and NADPH. Rickettsiae acquire SAM directly from the host via the highly conserved EamA transporter (see Fig. S10). Interestingly, *R. prowazekii* can import SAM (53) yet also generate it from host-acquired ATP and l-Met using the SAM synthase MetK (87). This appears to be the exception rather than the rule, however, as MetK-encoding genes are pseudogenized in most other *Rickettsia* species (data not shown). Our observations are in line with the proposition that the acquisition of metabolite transport systems facilitates the degradation of apposite biosynthetic pathways in rickettsiae (13). Remarkably, the EamA transporters were found piggybacking on integrative conjugative elements that appear to seed *Rickettsia* genomes with genes associated with intracellular invasion and survival (60); lateral transfer of such elements has occurred with obligate intracellular members of the phylum *Bacteroidetes* (88).

**FIG 4:**
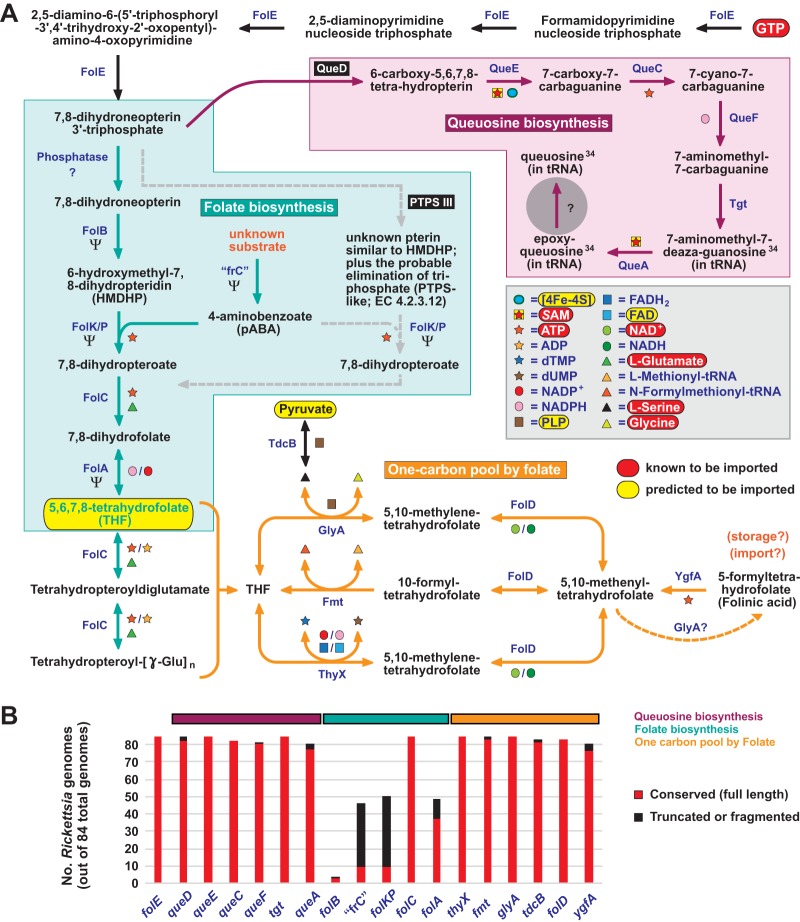
*Rickettsia* species lack the capability for *de novo* folate biosynthesis. (A) Rickettsiae use GTP cyclohydrolase I (FolE) to convert host-acquired GTP (red) to DHN-P_3_, a precursor of both queuosine (purple) and folate (aquamarine) biosynthesis. The classical THF synthesis pathway (aquamarine arrows), wherein DHN-P_3_ is dephosphorylated and subsequently converted to HMDHP by dihydropteridin aldolase (FolB), is disintegrating from *Rickettsia* genomes (Ψ denotes pseudogenization in over 50% of genomes). In the FolB bypass proposed by Hunter et al. (89) (gray dashed arrows), DHN-P_3_ is directly converted to HMDHP or a structurally similar molecule via PTPS-III (black box). Our analysis instead suggests that this enzyme is QueD (black box), which performs the first committed step in queuosine biosynthesis (see the text for further details). The one-carbon pool by folate (orange arrows) illustrates the role of host-acquired THF and several intermediates in the essential one-carbon transfer reactions that yield pyrimidine deoxynucleoside triphosphates, *N*-formylmethionyl-tRNA, and Ser/Gly. “frC,” fol_rel_CADD domain-containing protein (TIGR04305). The gray circle represents a hole in the pathway for queuosine synthesis (see Fig. S6). (B) Conservation of 18 genes involved in queuosine and THF biosynthesis and reactions within the one-carbon pool by folate. The complete distributions of these genes in 84 *Rickettsia* genomes reveals that no single *Rickettsia* species is capable of *de novo* folate biosynthesis, while the queuosine biosynthesis and one-carbon pool by folate pathways are highly conserved (see Fig. S5D).

Regarding vitamin B_9_ (folate), comparative genomics originally indicated a degraded biosynthesis pathway in *Rickettsia* genomes (i.e., one or more genes encoding FolB, FolK/P, and FolA were missing) and suggested that folate derivatives must be imported from the host (7, 48). More recently, Hunter et al. (89) proposed that the *Rickettsia* endosymbiont of *Ixodes pacificus* (REIP) can synthesize the active form of the vitamin, tetrahydrofolate (THF), and provision it to its arthropod host. REIP is closely related to *R. buchneri*, a species found exclusively in *Ixodes scapularis*, the tick vector of Lyme disease (90). REIP and *R. buchneri* occur in high frequency in ticks and are not known to cause disease in vertebrates, collective factors reflecting a possible symbiosis between the host and microbe (90–94). The proposal for REIP provisioning THF to its tick host was based on the identification of a putative PTPS-III enzyme, a 6-pyruvoyltetrahydropterin synthase (PTPS) with an atypical active site, that can functionally replace FolB (95) (Fig. 4A). This “FolB bypass” is a strategy employed by some microbes wherein PTPS-III can directly convert 7,8-dihydroneopterin 3′-triphosphate (DHN-P_3_) to 6-hydroxymethyl-7,8-dihydropteridin (HMDHP), a function mediated by Glu replacing or accompanying the usual Cys in the PTPS active site (96). This subtle active-site modification alters the typical functions of PTPS enzymes, which include the synthesis of biopterin (97, 98) and queuosine (99, 100). Queuosine is a modified nucleoside that can occupy the first anticodon position of tRNAs for His, Asp, Asn, and Tyr (101), and its synthesis involves the conversion of DHN-P_3_ to 6-carboxy-5,6,7,8-tetra-hydropterin by the (canonical) PTPS enzyme QueD (Fig. 4A).

In the present study, we present several lines of evidence that argue against *de novo* THF synthesis in rickettsiae. First, the REIP enzyme proposed by Hunter et al. to be PTPS-III (QueD in our results) actually contains the canonical PTPS active site (see Fig. S5A). Our analysis further reveals that rickettsial PTPS enzymes are highly conserved across *Rickettsia* genomes, and none of them contain the active-site modification associated with FolB bypass functionality (see Fig. S5B). Second, QueD catalyzes the first committed step in queuosine biosynthesis (Fig. 4A); surprisingly, this pathway is extremely conserved across the rickettsiae (including REIP), with the exception of a pathway hole at the terminal step converting epoxyqueuosine to queuosine (see Fig. S6). It seems unlikely that QueD would function in the synthesis of both 6-carboxy-5,6,7,8-tetrahydropterin (queuosine pathway) and HMDHP (folate pathway) from the same initial substrate (DHN-P_3_). Third, we show that it is unlikely that rickettsiae are unable to synthesize the 4-aminobenzoate (pABA) that is incorporated into the pterin ring of HMDHP, as the enzymes that would normally generate pABA from chorismate (namely, PabA/B and PabC) are not present, and chorismate itself is not synthesized and probably not needed by rickettsiae (discussed above for PHBA metabolism). Some genomes do encode “frC,” an atypical fol_rel_CADD domain-containing enzyme (TIGR04305) (102) that may provide a path to pABA generation from an unknown precursor (Fig. 4B; see Fig. S5D); however, the “frC” locus is degraded in almost all rickettsiae (including REIP) and is not likely to be functional. Fourth, phylogeny estimation indicates that rickettsiae once contained the canonical THF synthesis pathway that is complete in most other lineages of *Rickettsiales* (see Fig. S5C). Fifth, our analysis has found that every *Rickettsia* genome harbors one or more Fol-encoding pseudogenes (Fig. 4B; see Fig. S5D). While we did not explore the possibility that certain *Rickettsia* lineages have reacquired folate synthesis functionality via lateral transfer, collectively, our findings indicate that rickettsial genomes are characterized by an eroding folate biosynthesis pathway, with retention of only the enzymes necessary for the initiation of queuosine biosynthesis (FolE, QueD) and carrying out glutamylation of host-acquired THF (FolC). Thus, we predict that rickettsiae import host THF (and/or its derivatives, including folinic acid) to carry out the critical one-carbon transfer reactions involved in amino acid interconversion, pyrimidine synthesis, and translation.

10.1128/mBio.00859-17.5 FIG S5  *Rickettsia* species use DHN-P_3_ for queuosine biosynthesis and import host THF for one-carbon transfer reactions by folate. Download FIG S5, PDF file, 1.3 MB. Copyright © 2017 Driscoll et al. 2017 Driscoll et al. This content is distributed under the terms of the Creative Commons Attribution 4.0 International license.

10.1128/mBio.00859-17.6 FIG S6  The *Rickettsia* queuosine biosynthesis pathway contains a hole for the reduction of epoxyqueuosine to queuosine. Download FIG S6, PDF file, 0.4 MB. Copyright © 2017 Driscoll et al. 2017 Driscoll et al. This content is distributed under the terms of the Creative Commons Attribution 4.0 International license.

### Whole pathways or pathway holes?

**FIG 5:**
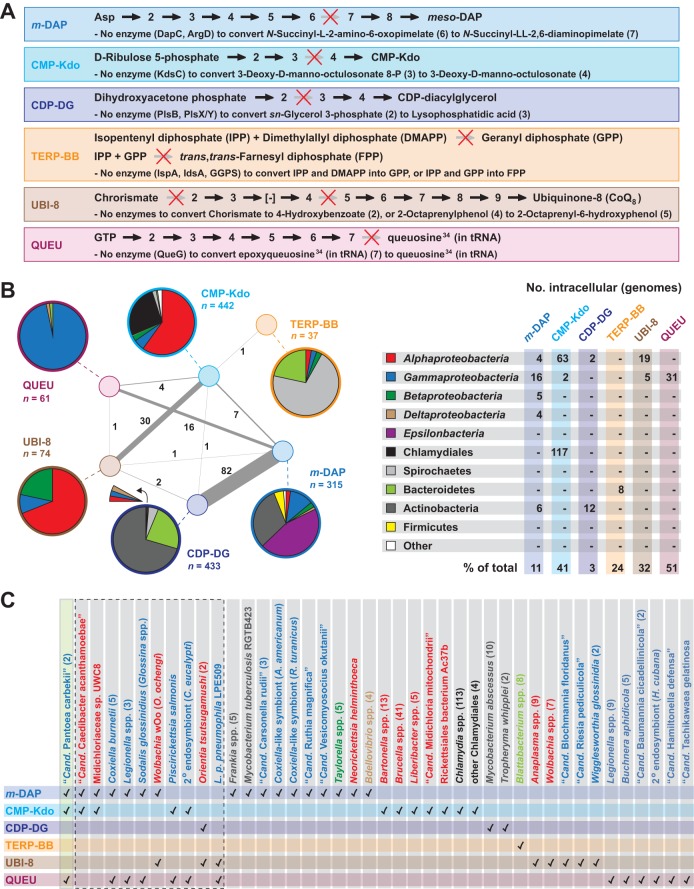
Comparative analysis of six biosynthetic pathways containing holes. The reconstructed *Rickettsia* metabolic network revealed holes in six biosynthetic pathways, DAP (*m*-DAP), CMP-Kdo, CDP-diacylglycerol (CDP-DG), terpenoid backbones (TERP-BB), ubiquinone-8 (UBI-8), and queuosine (QUEU). The distribution of these pathways across other prokaryotic genomes was determined via comparative metabolic pathway analyses. (A) Illustration and description of each hole-containing biosynthetic pathway. A red X indicates the missing enzyme(s) within each pathway, compared to well-characterized biosynthetic pathways for other prokaryotes. (B) Distribution of *Rickettsia*-like biosynthetic pathways across other prokaryotic genomes. The pie charts at the left indicate the taxonomic breakdown of genomes per biosynthetic pathway, with connections between pathways illustrating the number of genomes containing multiple *Rickettsia*-like pathways. Note that *Rickettsia* genomes were excluded from these analyses. At the right, the taxonomic color scheme is shown, with the number of genomes from intracellular species provided. (C) Intracellular species containing one or more *Rickettsia*-like biosynthetic pathways. The green box depicts the only genome found to contain three *Rickettsia*-like pathways, that of “*Ca.* Pantoea carbekii”) (117), which is an extracellular primary symbiont of the brown marmorated stink bug, where it is found in the gastric cecal lumina (see the text for further details). The dashed box indicates genomes containing two *Rickettsia*-like pathways. Taxa are colored in accordance with the color scheme in panel B.

In total, our *Rickettsia* metabolic reconstruction identified six holes occurring in otherwise conserved pathways for the synthesis of DAP, CMP-Kdo, CDP-diacylglycerol (CDP-DG), terpenoid backbones, CoQ_8_, and queuosine (Fig. 5A). Evaluation of the occurrence of these pathway holes across other bacterial genomes revealed that rickettsiae are not unique in lacking homologs of the enzymes that typically carry out these reactions (Fig. 5B). Surprisingly, the “*Rickettsia*-like” pathways for DAP, CMP-Kdo, and CDP-DG biosynthesis were found in a large number of bacterial genomes. For DAP biosynthesis, several alternative routes for the conversion of 2,3,4,5-tetrahydrodipicolinate to DAP have been characterized (103–105), though rickettsiae do not encode any of the enzymes in these other pathways (see Fig. S2D). The large number (*n* = 360) and diversity of bacterial genomes simply lacking DapC within the predominant DAP pathway likely indicate the existence of an alternative aminotransferase that awaits experimental characterization. Similarly, within the CMP-Kdo biosynthesis pathway, the lack of KdsC homologs across divergent bacteria indicates that these species may use a different, possibly nonspecific, phosphatase to convert 3-deoxy-d-manno-octulosonate 8-P to 3-deoxy-d-manno-octulosonate. In support of this, KdsC is not essential in *E. coli* (106). Importantly, *Rickettsia* genomes encode multiple haloacid dehalogenase-like hydrolases (InterPro domain IPR023214) (60) that may provide functions similar to those of KdsC, which also possesses this domain.

For CDP-DG synthesis, the lack of a G3P acyltransferase that converts G3P to LPA early in the synthesis of glycerophospholipids is puzzling. The ability to import G3P or import DHAP and convert it to G3P (51, 52), combined with the lack of other metabolic pathways that would use G3P directly, indicates that rickettsiae need to acquire G3P primarily for glycerophospholipid biosynthesis. For acylation of the G3P 1-position, bacteria typically use either PlsB, which primarily uses acyl-ACP as the fatty acyl donor, or PlsY, which uses acyl-PO_4_ produced from acyl-ACP by PlsX (107) (see Fig. S3A). In addition to rickettsiae, our analysis identified 433 other bacterial genomes that lack the genes encoding PlsB, PlsX, and PlsY (Fig. 5B), indicating that additional G3P acyltransferases remain to be characterized. The presence in 190 bacterial genomes of PlsX without either acyltransferase further supports this determination (see Fig. S3B) and may indicate that as-yet-unidentified G3P acyltransferases could use acyl-PO_4_ similar to PlsY. The 433 *Rickettsia*-like genomes that lack all three enzymes are found predominantly in species of *Actinobacteria* and *Bacteroidetes* (see Fig. S3C); comparative studies that include rickettsiae and these genomes may prove fruitful for identifying novel G3P acyltransferases.

The *Rickettsia*-like pathways for the synthesis of terpenoid backbones, CoQ_8_, and queuosine are far less common in other bacteria (Fig. 5B). For terpenoid backbone synthesis, 37 genomes were found that contained only genes encoding Idi, IspB, and IspU (see Fig. S4A and B), indicating that it is rare for bacteria to generate terpenoid backbones without the ability to synthesize isoprene (IPP, GPP) and terpenoid (FPP) precursors. Regarding CoQ_8_ synthesis, *ubiI* and *ubiC* occur far less frequently than other *ubi* genes in bacterial genomes (see Fig. S4D). Aside from the nonessentiality of UbiI in *E. coli* (discussed above), a very recent report proposed reannotation of the various proteobacterial CoQ_8_ flavin monooxygenases (UbiI, UbiH, UbiF, Coq7) into two novel groups named UbiM and UbiL (108). For UbiL (strictly *Alphaproteobacteria* proteins previously annotated as UbiH, such as all rickettsial UbiH proteins), the enzyme from *Rhodospirillum rubrum* was shown to complement Δ*ubiI* and Δ*ubiH* mutant *E. coli* strains, suggesting that the missing UbiI functionality may be carried out by UbiH in rickettsiae. Regarding UbiC (chorismate lyase), an alternative means to convert chorismate to PHBA was previously discovered for *Xanthomonas* species (109), indicating that other strategies may yet be uncovered to explain the lack of *ubiC* in so many bacterial genomes. Still, for rickettsiae and other intracellular bacteria that not only lack the shikimate pathway for the generation of chorismate but are devoid of the enzymes for the generation of aromatic amino acids from chorismate, transport systems must exist for PHBA acquisition from the host. Pathways that lack UbiC in other bacterial genomes may facilitate the identification of alternative mechanisms for the generation (or acquisition) of PHBA to fuel CoQ_8_ synthesis (see Fig. S4D and E).

The lack of the terminal enzyme (QueG) in the *Rickettsia* queuosine synthesis pathway implies either that the penultimate product, epoxyqueuosine, occupies position 34 (anticodon wobble position) in tRNAs with GU_N_ anticodons or that another epoxyqueuosine reductase unrelated to QueG is found in rickettsial genomes. The latter scenario seems more plausible on the basis of two lines of evidence. First, QueG requires the cofactor cobalamin (vitamin B_12_) (110, 111), a large modified tetrapyrrole synthesized by dozens of enzymes (112). Rickettsiae cannot synthesize cobalamin, and no cobalamin-dependent enzymes are encoded within rickettsial genomes (data not shown), indicating that this cofactor is likely not imported. Thus, the absence of QueG correlates with the inability to provide its cofactor, cobalamin. In support of this, none of the 61 bacterial genomes that contain the *Rickettsia*-like queuosine pathway encode enzymes needed to synthesize cobalamin (data not shown). Second, 45 of these genomes that lack QueG, as well as all rickettsial genomes, encode DUF208-containing proteins (data not shown), which have very recently been characterized as cobalamin-independent epoxyqueuosine reductases unrelated to QueG (113). Genes encoding these proteins (renamed QueH) are often clustered with Que-encoding genes in bacterial genomes. In rickettsial genomes, *queH* is not physically linked to other Que-encoding genes, though it flanks a gene encoding another tRNA-associated protein, the glycine-tRNA ligase alpha subunit. Regardless, it seems reasonable to postulate that rickettsiae may use QueH to convert epoxyqueuosine to queuosine, although this awaits experimental confirmation.

We analyzed bacteria containing these six *Rickettsia*-like pathway holes on the basis of their lifestyle (i.e., extracellular versus intracellular) to explore whether reductive genome evolution as a consequence of an intracellular lifestyle might be a driving factor for gene decay within these pathways (Fig. 5B). In most cases, intracellular bacteria from only one or two phylogenetic groups contained *Rickettsia*-like pathways; for example, the *Rickettsia*-like queuosine pathway appeared solely in *Gammaproteobacteria*, while the terpenoid pathway was found only in *Bacteroidetes*. The exception is DAP, for which the *Rickettsia*-like pathway was distributed across intracellular species from five groups. The majority of intracellular bacteria that harbor *Rickettsia*-like pathways contain only one, with 10 taxa containing two (Fig. 5C). It is intriguing to consider that the first genes to decay within obsolete metabolic pathways might hinder but not entirely arrest metabolite biosynthesis, such that the microbe slowly becomes chemically addicted to a host-derived substrate. Suboptimal metabolic pathways may contribute to a low growth rate, a hallmark of rickettsiae (114, 115), which in turn may be advantageous for pilfering metabolites from the eukaryotic cytoplasm while preventing destruction of the host cell.

Nearly all extracellular bacteria that harbor *Rickettsia*-like pathway holes contain only one, with the remarkable exception of “*Candidatus* Pantoea carbekii,” which contains three (DAP, CMP-Kdo, and queuosine). Despite an extracellular lifestyle, this gammaproteobacterial species is a primary symbiont of the brown marmorated stink bug, where it resides primarily in the gastric cecal lumina (116). The “*Ca.* Pantoea carbekii” genome is minimal in size (1.2 Mb), and its gene repertoire contains characteristics of both highly specialized obligate mutualists and facultative species (117). Interestingly, we also identified a potential pseudogene for PlsB in the “*Ca.* Pantoea carbekii” glycerophospholipid pathway, suggesting a fourth possible *Rickettsia*-like pathway hole in this symbiont. This stunning degree of convergent evolution in metabolic pathways between rickettsieae and “*Ca.* Pantoea carbekii” may reflect common modes for pathway modification in the presence of host-acquired metabolites. Altogether, our work illuminates common and rare deviations from conserved metabolic pathways, with similar pathways in unrelated bacteria indicating convergence and possible novel enzymes awaiting characterization.

### Black hole son: reductive evolution from mother *Rickettsia*.

To better understand the evolutionary trajectory of the six *Rickettsia* pathway holes, we analyzed their composition in the genomes of other taxa in the orders *Rickettsiales* and *Holosporales* (see Fig. S7A and B). Genes encoding KdsC, PlsB, UbiC, and UbiI were not detected in any other *Rickettsiales*/*Holosporales* genomes, indicating that rickettsial pathways for CMP-Kdo, CDP-DG, and CoQ_8_ likely never included these enzymes. In contrast, nearly all members of the orders *Rickettsiales* and *Holosporales* possess DapC, PlsX/Y, and IspA, with phylogeny estimation supporting their vertical inheritance from a proteobacterial ancestor (see Fig. S7C to F). Thus, it can be parsimoniously inferred that the pathway holes found in *Rickettsia* DAP, CDP-DG, and terpenoid backbone synthesis pathways reflect reductive evolution that has occurred since divergence from the *Rickettsiales*/*Holosporales* ancestor.

10.1128/mBio.00859-17.7 FIG S7  The evolutionary trajectory of six *Rickettsia* biosynthetic pathways that contain holes, or “missing enzymes.” Download FIG S7, PDF file, 2.2 MB. Copyright © 2017 Driscoll et al. 2017 Driscoll et al. This content is distributed under the terms of the Creative Commons Attribution 4.0 International license.

The reduction in DAP, CDP-DG, and terpenoid backbone synthesis pathways is seen early within the family *Rickettsiaceae*, where “*Candidatus* Arcanobacter lacustris” (118) lacks PlsX/Y. DapC, PlsX/Y, and IspA are also missing from the *Rickettsia* sister lineage, which includes the scrub typhus agent (*Orientia tsutsugamushi*), *Orientia chuto* (119), and “*Candidatus* Occidentia massiliensis” (120). These results are not surprising, given that *O. tsutsugamushi* genomes are laden with proliferative/degradative mobile genetic elements (9, 121) and have more missing metabolic enzymes than *Rickettsia* species do (5, 8, 49). This *Rickettsia* sister clade lacks *idi* and must therefore acquire both IPP and DMAPP (in addition to FPP) from the host to initiate the CoQ_8_ and terpenoid backbone synthesis pathways. All other *Rickettsiales*/*Holosporales* genomes likewise lack *idi*, though they do encode enzymes of the MEP/DOXP pathway for the synthesis of isoprenes, indicating that IPP-DMAPP interconversion is unique to *Rickettsia* species. Remarkably, phylogeny estimation suggests that rickettsiae likely acquired *idi* from non-*Alphaproteobacteria* sources (Fig. S8A).

10.1128/mBio.00859-17.8 FIG S8  Phylogeny estimation of Idi and queuosine biosynthesis proteins. Download FIG S8, PDF file, 0.6 MB. Copyright © 2017 Driscoll et al. 2017 Driscoll et al. This content is distributed under the terms of the Creative Commons Attribution 4.0 International license.

Epoxyqueuosine reductases (QueG or QueH) were not identified in any other *Rickettsiales*/*Holosporales* genome besides that of *Rickettsia* (see Fig. S7A and B). Unexpectedly, only “*Candidatus* M. mitochondrii” was found to contain the other Que enzymes (QueD, QueE, QueC, QueF, and QueA but not QueH). The strict conservation of Tgt in all genomes suggests that species lacking Que-encoding genes incorporate host-acquired queuine directly into tRNAs with GU_N_ anticodons, similar to the eukaryotic salvage mechanism (122). Phylogeny estimation indicates two origins of Tgt in *Rickettsiales*, with one clade consisting entirely of taxa that lack the Que-encoding genes (*Anaplasmataceae* and *Holosporales*) (Fig. S8B). The origins of the Que-encoding genes themselves are difficult to estimate with confidence (Fig. S8C to H); thus, it cannot be determined if they were present in the *Rickettsiales* ancestor and subsequently lost by all lineages except rickettsiae and “*Ca.* M. mitochondrii” or if these two lineages independently acquired them. Why rickettsiae and “*Ca.* M. mitochondrii” would generate queuosine from GTP rather than use less complex salvage mechanisms found in other members of the orders *Rickettsiales* and *Holosporales* is difficult to understand. Regardless, in conjunction with the estimated nonalphaproteobacterial origin of rickettsial *idi*, this analysis implies that rickettsiae can acquire metabolic enzymes throughout evolution to offset gene decay.

### So many imported metabolites, so few characterized transporters.

**FIG 6:**
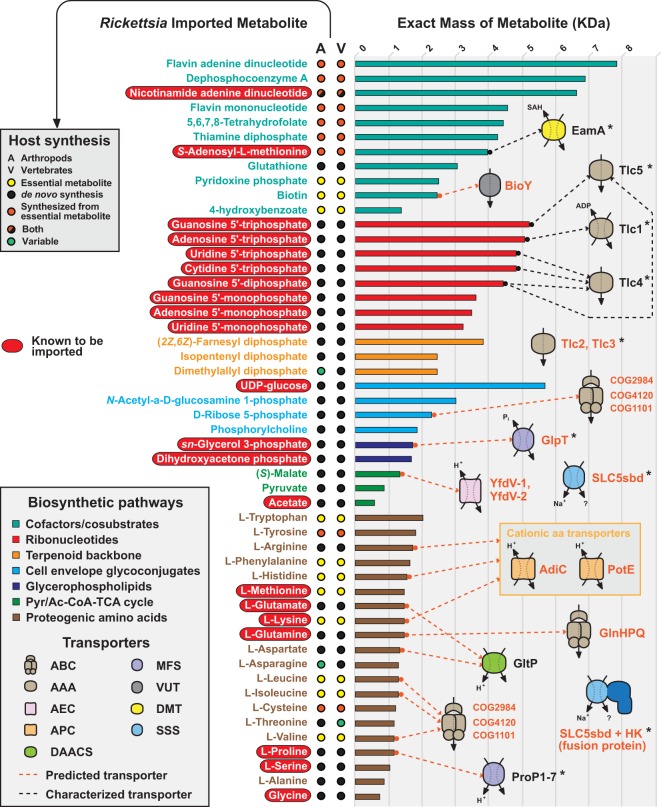
Synopsis of known and predicted metabolites imported from the eukaryotic cytoplasm by rickettsiae. On the left, metabolites are grouped into biosynthetic pathways (colors are described in the inset at the bottom left), with red ellipses depicting 21 metabolites previously shown to be imported. The remaining 30 metabolites are predicted to be imported on the basis of the metabolic network reconstruction presented in this report. In the center are the biosynthesis capabilities of the metabolites in arthropod and vertebrate genomes (further described in the inset at the top left). Information was obtained from KEGG pathways for arthropods and vertebrates. On the right, within each group, metabolites are ranked by exact mass. Dashed lines connect metabolites with their known (black) or predicted (orange) transport systems (transporter families are listed in the inset at the bottom left). One ABC transporter (COG1101/COG4120/COG2984) is shown twice, as annotations indicate uptake of branched-chain amino acids, as well as monosaccharides (including ribose, galactose, and arabinose). SLC5sbd and SLC5sbd+HK (fusion protein with His kinase domain) transporters are not linked with specific metabolites because of their known broad range of substrates (e.g., sugars, amino acids, organo-cations such as choline, nucleosides, inositols, vitamins, urea, or anions). Asterisks indicate transporters previously shown to be associated with mobile genetic elements and/or predicted to be spread by lateral gene transfer across diverse intracellular bacteria (60, 88, 126, 127). Transporter names and family identifications (123) are as follows: ABC, ATP-binding cassette (3.A.1); AAA, ATP:ADP antiporter (2.A.12); DMT, drug/metabolite transporter (2.A.7); VUT, vitamin uptake transporter (2.A.88); AEC (2.A.69); MFS, major facilitator superfamily (2.A.1); APC, amino acid polyamine organo-cation (2.A.3); DAACS, dicarboxylate/amino acid:cation (Na^+^ or H^+^) symporter (2.A.23); SSS, solute:sodium symporter (2.A.21). Phylogenomics analysis indicates that these transporters are highly conserved in rickettsial genomes (see Fig. S10).

In total, our reconstructed metabolic network predicts that rickettsiae must import 51 host metabolites to supplement their patchwork metabolic pathways (Fig. 6). This adds 30 metabolites to the 21 previously shown to be imported by rickettsiae in either *in vivo* or *in vitro* assays. Roughly 80% of these metabolites are synthesized by both vertebrates and arthropods, with the other metabolites essential for both the host and the microbe (Fig. 6, circles at center). Importantly, the metabolites targeted by rickettsiae all belong to highly conserved eukaryotic pathways, which explains their ability to grow in a wide range of cell types. With such a high degree of metabolite thievery, rickettsiae must have in place an arsenal of transport systems to ensure that all of the essential metabolites are imported from the eukaryotic cytoplasm in sufficient quantities to support their growth. This also implies a remarkable level of regulatory control over these systems to avoid starving the host cell of its own essential metabolites too quickly.

Based on a combination of previously characterized transporters and *in silico* transporter prediction (123), we compiled a modest set (*n* = 24) of transporters that are highly conserved across *Rickettsia* genomes (Fig. 6; see Fig. S10). Some transport systems (i.e., those involved in drug efflux, PGN recycling, phospholipid maintenance, etc.) were not considered. Alignment of this transporter set with our set of 51 imported metabolites illustrates that ribonucleotides have the most characterized transport pathways (38, 40–42, 54). Despite unknown transporters for the ribonucleotide monophosphates, our reconstruction shows that the eight ribonucleotides known to be imported can be used by rickettsiae to synthesize the remaining ribonucleotides and all of the deoxyribonucleotides required for replication, transcription, and regulation of the stringent response (see Fig. S9). The only other characterized transporter in our set is EamA, the SAM antiporter (53). Transporters for proteogenic amino acids have not been characterized, despite evidence for rickettsial import of Met, Glu, Lys, Gln, Pro, Ser, and Gly (22–24, 30–37). Predicted transporters of charged amino acids (AdiC, PotE, GltP), Gln (GlnHPQ), and branched-chain amino acids (COG2984/COG4120/COG1101) account for only half of the amino acids that rickettsiae need to import. The substrate ranges of seven pro/betaine symporters (ProP), which typically exchange proline and other osmolytes during osmoregulation (124), might account for the remaining amino acids, given that conserved ProP groups are highly divergent from one another (60). These and/or other transporters must be operational to counterbalance the absence of nearly all amino acid biosynthesis pathways.

10.1128/mBio.00859-17.9 FIG S9  *Rickettsia* transport and metabolism of host-acquired ribonucleotides. Download FIG S9, PDF file, 0.5 MB. Copyright © 2017 Driscoll et al. 2017 Driscoll et al. This content is distributed under the terms of the Creative Commons Attribution 4.0 International license.

Very few transporters could be predicted for other characterized (NAD, UDP-glucose, DHAP, G3P, and acetate) or putative (all others) imported metabolites. Both branched-chain amino acids and d-ribose 5-P are considered possible substrates of the ABC transporter COG2984/COG4120/COG1101. G3P is possibly transported by the known ATP-independent G3P transporter GlpT, which is highly conserved in rickettsiae; however, G3P import by *R. prowazekii* was shown to require ATP, indicating the likelihood of another transporter (52). As discussed above, duplicate YfdV transporters are predicted to import malate and possibly pyruvate. A single component of the BioMNY transporter (125), BioY, is conserved in rickettsial genomes and likely imports biotin. Finally, all rickettsial genomes encode at least one SLC5sbd transporter, which is a member of the solute:sodium symporter family. These transporters are widespread across prokaryotes and eukaryotes and work on a wide range of characterized substrates, including sugars, amino acids, organo-cations such as choline, nucleosides, inositols, vitamins, urea, and anions. Some rickettsial SLC5sbd transporters are fused with a BaeS-like, two-component signal transduction histidine kinase (HK) domain. Both SLC5sbd and SLC5sbd+HK proteins are highly proliferated in *Orientia* and *Occidentia* genomes (data not shown). While the substrates of these transporters are unknown, their importance in obligate intracellular living is evident, given their spread by lateral transfer across rickettsiae and unrelated obligate intracellular bacteria such as “*Candidatus* Amoebophilus asiaticus” and *Cardinium* endosymbionts of arthropods (60, 126, 127).

The requirement for several large metabolites (FAD, dephospho-CoA, NAD, FMN, THF, ThDP, FPP, UDP-glucose) implies that rickettsiae must have transport systems to import these molecules. We did not identify any of the energy-coupling factor transporters (128–130) typically associated with the uptake of large vitamins (or their derivatives) such as folate (131), riboflavin (132), and thiamine (133, 134). Despite demonstration that a TLC protein of environmental chlamydiae can transport NAD^+^ (135), there is little sequence similarity between this transporter and rickettsial TLC proteins (60). The *Rickettsia* SLC5sbd transporter shows mild similarity (~23% identical) to the *E. coli* sodium/pantothenate symporter PanF; however, as discussed above, rickettsiae most likely import dephospho-CoA and not pantothenate, since they cannot metabolize the latter. Finally, we did not identify any transporters for other carbohydrates, isporenes, or terpenoids.

The paucity of transporters relative to the high number of imported metabolites suggests that either the characterized and predicted transporters are not substrate specific or there are other transport systems that remain unidentified. It is worth considering as well that host transport systems may be hijacked by rickettsiae to ensure the delivery of metabolites that are not known to be transported by bacteria (e.g., FAD, UDP-glucose, dephospho-CoA, FPP). Given the possible shared ancestry of rickettsiae and the mitochondrial progenitor (136), a reasonable host transporter to be used by rickettsiae might be the mitochondrial porin, which consists of the voltage-dependent anion channel (VDAC). Involved in the regulation of metabolic and energetic flux across the mitochondrial outer membrane (137), VDAC is already known to transport most of the molecules that rickettsiae need to complement their patchwork metabolic network. Curiously, VDAC was previously found to associate with the rickettsial cell envelope (138, 139), though the functional significance remains unclear. Given that recombinant VDAC can assemble in the outer membrane of *E. coli* (140), it is possible that VDAC forms functional porins on rickettsial cells. Determining if VDAC or other host transporters are co-opted during rickettsial acquisition of host metabolites is a fascinating area for future research.

### In light of metabolic parasitism, what, exactly, is a *Rickettsia* endosymbiont?

**FIG 7:**
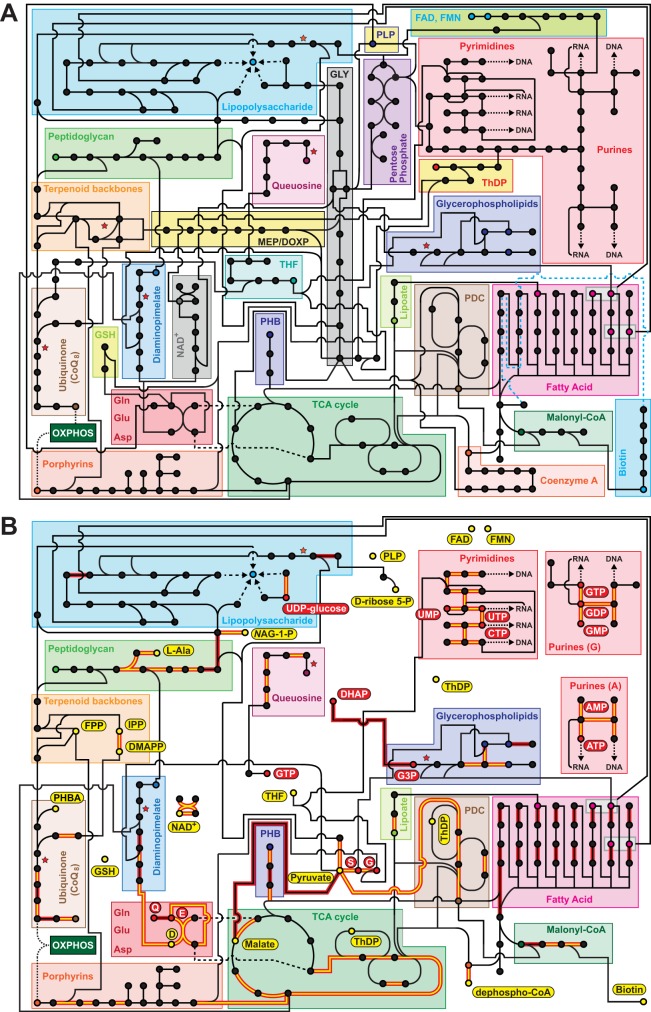
*Rickettsia* metabolic network reconstruction highlights reductive genome evolution and addiction to host cell metabolites. The network focuses on the biosynthesis pathways discussed in the text. For brevity, pathways for most amino acids are not shown. Red stars indicate six pathway holes (see Fig. 5). GLY, glycolysis; MEP/DOXP, nonmevalonate terpenoid biosynthesis; THF, 5,6,7,8-THF; GSH, glutathione. (A) Theoretical *Rickettsia* metabolic network in the absence of imported metabolites. *Rickettsia* metabolic pathways are supplemented with typical Gram-negative biosynthetic pathways to create a complete metabolic network. (B) Reconstructed *Rickettsia* metabolic network, including imported metabolites. Pathways removed from panel A have been purged from *Rickettsia* genomes throughout evolution, a consequence of pilfering of metabolites from the eukaryotic host. Red ellipses, metabolites known to be imported by *Rickettsia* species; yellow ellipses, metabolites predicted to be imported on the basis of metabolic network reconstruction. The import of *S*-adenosyl-l-methionine, phosphorylcholine, and the majority of amino acids is not included in the reconstruction. Pathway lines are highlighted in red to indicate cofactors that are synthesized directly from imported metabolites. Additionally, if the cofactor is directly imported from the host, the pathway line is yellow. The network is based on a phylogenomics analyses of 84 *Rickettsia* genomes (see Fig. S10).

Our metabolic reconstruction illustrates the few biosynthesis pathways that remain in rickettsiae. In most free-living and facultative intracellular bacteria, these pathways are interconnected primarily by glycolysis and the pentose phosphate pathway, two of the most conserved metabolic processes across life’s three domains (Fig. 7A). Rickettsiae have replaced these core biosynthesis hubs by highly elaborate thievery of host metabolites to complement its patchwork metabolic network (Fig. 7B). Imported metabolites are used to initiate many other highly conserved biosynthetic pathways (e.g., PDC, TCA cycle, porphyrins, glycerophospholipids, nucleotides), as well as pathways unique to bacteria (e.g., LPS, PGN, DAP, PHB, CoQ_8_, queuosine, type II fatty acid synthesis). To this end, the *Rickettsia* metabolic network may approach the minimal metabolic unit for a bacterial parasite of the eukaryotic cytoplasm.

10.1128/mBio.00859-17.10 FIG S10  Phylogenomics analysis of *Rickettsia* metabolic pathways and metabolite transporters. Download FIG S10, XLSX file, 0.4 MB. Copyright © 2017 Driscoll et al. 2017 Driscoll et al. This content is distributed under the terms of the Creative Commons Attribution 4.0 International license.

Most reactions within the remnant pathways rely on vitamins and cofactors that are also pilfered from the host (Fig. 7B). While rickettsiae were previously considered facultative energy parasites because of their ability to not only steal host ATP but also generate their own via oxidative phosphorylation, their reliance on numerous host metabolites to drive ATP production accentuates the obligate parasitic nature of rickettsiae. Prior comparative genomics studies of diverse rickettsiae have identified differences between vertebrate pathogens and species/strains with little or no documented pathogenicity, with loss of virulence often attributed to the expansion of mobile genetic elements that have destroyed genes implicated in vertebrate cell colonization (60, 61). Furthermore, the genomes of attenuated pathogens, e.g., *R. prowazekii* strain Madrid E (141) and *R. rickettsii* strain Iowa (142, 143), contain defects in some genes implicated in vertebrate pathogenicity. Importantly, our metabolic network reconstruction included 84 *Rickettsia* genomes encompassing the full range from deadly pathogens to purported endosymbionts, including species from basal lineages associated with nonhematophagous arthropods (the ladybird beetle *Adalia bipunctata* [59, 144]) and a parasitic ciliate (*Ichthyophthirius multifiliis* [145, 146]), as well as a recently identified spotted fever group species discovered in an unlikely vector, the seal fur louse *Proechinophthirus fluctus* (147). Although complicated by some genome assemblies of poorer quality, our reconstructed metabolic network is highly conserved across all of these species and strains, indicating that, despite various impacts on host fitness, all sequenced rickettsiae fit the profile of a quintessential and comprehensive metabolic parasite of the eukaryotic cytoplasm.

Like rickettsiae, other obligate intracellular bacteria have reductive genomes, yet genuine endosymbionts tend to retain pathways for the synthesis of amino acids, vitamins, and cofactors (148)—even some endosymbionts of blood-feeding arthropods (149, 150). These metabolites are often provisioned to the host, with the host usually harboring the bacteria in a protective niche (e.g., a bacteriocyte). *Rickettsia* species associated with the silverleaf whitefly (*Bemisia tabaci* [151]) and the booklouse *Liposcelis bostrychophila* (152), both nonhematophagous arthropods, have been observed in bacteriocytes; however, only the *L. bostrychophila* system is considered a primary endosymbiosis, as its *Rickettsia* species is always present in parthenogenetic females (153). The booklouse *Rickettsia* was determined to be a strain of *Rickettsia felis*, a species that is usually associated with blood-feeding arthropods and may be an occasional human pathogen (154). Our previous phylogenomics study revealed that *L. bostrychophila*-associated *R. felis* differs from other species by the presence of a novel plasmid, pLbAR, that encodes several factors not seen in other sequenced rickettsiae (155). It remains to be determined whether pLbAR contributes to the maintenance of the *L. bostrychophila*-*R. felis* symbiosis.

To our knowledge, no *Rickettsia* species has been shown to provision its host(s) with metabolites; indeed, rickettsiae seem to be in competition with arthropod and vertebrate cells for at least 20 metabolites that are either essential or synthesized from essential nutrients (Fig. 6). For strains of *R. buchneri*, our earlier discovery of a plasmid harboring two complete biotin synthesis operons raised the possibility that biotin could be provisioned to *I. scapularis* as part of a potential mutualism (60, 90). This unique biotin operon has since been discovered in other diverse obligate intracellular bacteria, including the *Cardinium* endosymbiont of the parasitic wasp *Encarsia pergandiella* (156) and the *Wolbachia* endosymbiont of the bedbug *Cimex lectularius* (*w*Cle) (157). Remarkably, *w*Cle-cured bedbugs that were fed a biotin-supplemented blood meal showed no marked differences in fitness relative to *w*Cle-infected bedbugs; together with more recent evidence that *w*Cle provisions riboflavin to its host (158), this indicates that evolutionary transitions from facultative parasitism to obligate mutualism have occurred in *Wolbachia* species, and they have been mediated by metabolic interdependence across host and microbe. It remains to be determined whether or not *R. buchneri* provisions biotin to *I. scapularis* or if any of the numerous *Rickettsia* species identified in hosts from most eukaryotic lineages (1) have entangled metabolic networks with their hosts. Barring some other benefit afforded to their host outside metabolism (i.e., competitive exclusion of pathogens [159, 160]) and given their metabolic profile as quintessential parasites of the eukaryotic cytoplasm, we stress the use of “endoparasite” over “endosymbiont” for these nonpathogenic rickettsiae.

### Conclusion.

Ancestral members of the order *Rickettsiales* were likely facultative intracellular species of protoeukaryotes, with a symbiosis established by one lineage that arguably facilitated eukaryogenesis (161). The remaining rickettsial lineages avoided spiraling into organelles or minimalist bacteria, instead relying on general features of the eukaryotic cell (including mitochondria) for growth. While many of the pioneering studies of *Rickettsia* metabolism analyzed bacteria purified away from host cells, it became clear that numerous essential host factors are required to adequately fuel the metabolic processes that support rickettsial growth. The advent of genome sequencing identified patchwork metabolic pathways and supported the notion that rickettsiae have become chemically addicted to the eukaryotic cell. To advance our knowledge beyond these observations, we reconstructed a surprisingly conserved metabolic core for rickettsiae and supplemented these patchwork metabolic pathways with metabolites predicted to be imported from the eukaryotic cell. As with any model, our reconstruction may have failed to predict some rickettsial metabolic potential or underestimated the number of metabolites acquired from the host; nevertheless, it provides an important framework upon which to structure future investigations. Some of the questions that arise from our model have broader application beyond rickettsiology as well, e.g., the potential discovery of novel transport systems or the identification of nonorthologous enzymes that function within canonical metabolic pathways.

Our study illustrates how little is known regarding rickettsial acquisition of host metabolites; in particular, it indicates that rickettsiae must use transport systems (possibly from the host) to import many large metabolites. Thus, future studies that characterize transporters in heterologous expression systems will continue to be invaluable tools in elucidating how rickettsiae pilfer host metabolites. We anticipate that careful evaluation of large-scale transcriptomic and proteomic data sets will reveal clues about how the host metabolism is hijacked throughout the course of rickettsial infection. Ultimately, establishing an axenic culture for rickettsiae will greatly facilitate genetic studies correlating transporters with imported substrates. Such an effort is conceptually appealing yet practically difficult. The present study represents an important contribution to that effort; it may be that differences between our estimated 51 essential metabolites and the complex media used in early metabolic assays (e.g., the recipe used by Bovarnick [71]) provide the “missing ingredients” necessary to support cell-free rickettsial growth.

Reductive genome evolution in rickettsiae has been interpreted by some as an ongoing process that, in its severest cases, correlates with an increase in vertebrate pathogenicity (162). We now understand that *Rickettsia* genomes continually acquire genes throughout their evolution and that genes encoding metabolic enzymes, metabolite transporters, and stringent response regulators are often components of mobile genetic elements (2, 60, 61, 155). This implies that there is strong selection on maintenance of the conserved rickettsial metabolome, which has evolved to allow rickettsiae to delicately parasitize the host cytoplasm, ensuring that large numbers of bacteria can persist before host cell destruction. This mode of parasitism allows for propagation throughout numerous arthropod tissues and, for some species, dissemination to vertebrates by blood feeding or fecal inoculation. Our work here provides novel insights into many aspects of rickettsial host-dependent metabolism. Metabolomics remains a poorly studied aspect of rickettsiology yet one where advancements would serve the fields of drug and vaccine design markedly well, particularly if certain host metabolites are scavenged using unique bacterial transport systems.

## MATERIALS AND METHODS

### Phylogenomics analysis. (i) Genus-level phylogeny estimation.

Protein sequences (*n* = 112,870) from 86 sequenced genomes were used to estimate a genus-level *Rickettsia* phylogeny. *Rickettsia* genomes were either retrieved from the NCBI Assembly database (*n* = 82) or obtained via personal communication (*n* = 2; Lucy Weinert, University of Cambridge) (see Fig. S10). Two non-*Rickettsia* genomes (*O. tsutsugamushi* strain Ikeda [accession no. GCF_000010205.1]; *Rickettsiales* bacterium Ac37b [GCF_000746585.1]) were included as outgroups. The RAST v 2.0 server (163) was used to annotate 16 *Rickettsia* assemblies that were not previously annotated. A total of 3,772 orthologous gene families were constructed from this data set using FastOrtho, a modified version of OrthoMCL (164), at an inflation of 1.5 and an identity threshold of 40%. A subset of 149 single-copy families conserved across all 86 taxa was independently aligned with MUSCLE v3.8.31 (165) using default parameters, and regions of poor alignment were masked using Gblocks (166). All modified alignments were concatenated into a single data set (41,975 positions) for phylogeny estimation using Phylobayes v4.1 (167), with the CAT model of substitution used as described previously (155). A phylogeny was also estimated under maximum likelihood with RAxML v8.2.4 (168) using a gamma model of rate heterogeneity and estimation of the proportion of invariable sites. Branch support was assessed with 1,000 pseudoreplications.

### (ii) Order-level phylogeny estimation.

Protein sequences (*n* = 86,029) from 56 sequenced genomes were used to estimate an order-level phylogeny of *Rickettsiales*/*Holosporales*, sampling the groups *Holosporales* (*n* = 9), *Anaplasmataceae* (*n* = 22), *Midichloriaceae* (*n* = 4), and *Rickettsiaceae* (*n* = 18), as well as three outgroup genomes (*Alphaproteobacteria* strain BAL199, *Azospirillum* sp. strain B510, and *Rhodospirillum rubrum* ATCC 11170). Genomes were retrieved from NCBI (25) and PATRIC (28) (see Fig. S7). A total of 8,437 orthologous families were constructed using FastOrtho (inflation of 1.2), and a subset of 105 single-copy families conserved across at least 52 taxa (95%) was aligned, masked, and concatenated (23,990 positions) as described above. Phylogeny estimation was carried out with PhyloBayes and RAxML as described above.

### Metabolic reconstruction.

For metabolic pathway analyses, the 84 *Rickettsia* genomes from our genus-level phylogeny estimation (outgroup genomes removed) were used to construct 3,576 orthologous gene families as described above. These families were subsequently assigned Kyoto Encyclopedia of Genes and Genomes (KEGG) K numbers by submitting all 109,191 individual protein sequences to the KEGG BlastKOALA service (169). K number assignments, as well as broader metabolic classifications, were extracted from the resulting HTML files (kcompile), transferred across orthologous families (ktransfer), and used for metabolic pathway reconstruction (kreconstruct). All of the software packages developed for this study are freely available in the keggerator repository on github.

### Identification of pathway holes.

A pathway hole is simply defined here as a missing gene in an otherwise complete metabolic pathway. Pathways that are absent or highly fragmented are not considered. Candidate pathway holes were initially identified during inspection of the metabolic reconstruction results for *Rickettsia* species. To account for possible errors in our K number annotation, gene absences were confirmed by searching (blastp) our data set with orthologous sequences from *E. coli*. The taxonomic distribution of confirmed *Rickettsia* pathway holes was assessed by converting each pathway into a binary feature vector, where each position in the vector represents a pathway step containing either a 1 (gene present) or a 0 (gene absent). Pathway data for all KEGG genomes were subsequently analyzed (khole) for genomes that possess *Rickettsia*-like feature vectors.

### Targeted phylogeny estimation.

To determine the evolutionary history of specific genes (e.g., those encoding Idi, enzymes of the folate and queuosine pathways, and *Rickettsiales* enzymes corresponding to *Rickettsia* pathway holes), individual protein phylogenies were estimated. To construct data sets for each protein, the rickettsial protein was used in blastp queries against several taxon-specific databases, i.e., (i) “*Rickettsiales*,” (ii) “*Holosporales*,” (iii) “*Alphaproteobacteria* (minus Rickettsiales and *Holosporales*),” (iv) “*Proteobacteria* (minus *Alphaproteobacteria*),” (v) “Bacteria (minus *Proteobacteria*),” and (vi) “minus Bacteria.” The top 5 to 10 (query-dependent) subjects from each search resulting in significant (>40 bits) alignments were all compiled and aligned with MUSCLE v3.8.31 using default parameters. Protein phylogenies were estimated under maximum likelihood with RAxML v8.2.4 using a gamma model of rate heterogeneity and estimation of the proportion of invariant sites. Both the Le and Gascuel (LG) and blocks of amino acid substitution matrix (BLOSUM62) models of amino acid substitution were used, and branch support was assessed using 1,000 pseudoreplications.

### Transporter prediction.

Diverse transport systems were assembled and annotated in an iterative process involving bioinformatics predictions, manual evaluation, and comparative genomics analyses. For the *R. typhi* strain Wilmington genome, 96 transporters were predicted using TransportDB (170). The breakdown of these predictions was ATP-dependent systems (*n* = 38), ion channels (*n* = 3), secondary transporters (*n* = 48), and unclassified transporters (*n* = 7). Several *Rickettsia* genomes were also evaluated on the Transporter Classification Database (TCDB) (123). Transporter names and family identifications were assigned after comparison across TransportDB and TCDB and, in some cases, blastp searches against genomes with well-characterized transporters. Subsequent manual evaluation eliminated erroneous predictions, components of protein translocation pathways, and other transport systems that were deemed not likely to import the host metabolites under consideration. Additionally, for tripartite ABC transporters, missing components were manually added on the basis of genomic proximity to other components likely to be part of the same transporter. Putative substrates for each transport system were conservatively assigned on the basis of TCDB or close homology of the rickettsial transporter to transporters with well-characterized substrates.
