# Supplementary material for: Wholly Rickettsia! Reconstructed Metabolic Profile of the Quintessential Bacterial Parasite of Eukaryotic Cells
Source: mBio. 2017 Sep 26;8(5):e00859-17. doi: 10.1128/mBio.00859-17 (PMC5615194; doi:10.1128/mBio.00859-17)
Supplement: FIG S1 [file mbo005173495sf1.pdf]

FIG S1. *Rickettsia* species import six host metabolites that are required for the synthesis of cell envelope glycoconjugates and glycerophospholipids.

(A) Consensus metazoan pathway for the generation of metabolites (UDP-glucose, *N*-acetyl-alpha-D-glucosamine 1-P (*N*AG-1-P), D-ribose 5-P, dihydroxyacetone P (DHAP), *sn*-glycerol 3-P (G3P), and pyruvate) that *Rickettsia* species must acquire to initiate biosynthesis of peptidoglycan (PGN), lipopolysaccharide (LPS) and glycerophospholipids. Red ellipses, metabolites previously shown to be imported; yellow ellipses, metabolites predicted to be imported based on metabolic network reconstruction. Gray shading depicts each point of the host metabolic network where *Rickettsia* species acquire metabolites, with blue text describing how rickettsiae utilize these metabolites. For biosynthesis of glycerophospholipids, *Rickettsia* species import G3P, but also can convert G3P from DHAP if *gpsA* (encoding glycerol-3-phosphate dehydrogenase) is present (shown in green) (1, 2).

(B) Schema and description of the bifunctional GlmU protein of *E. coli* (WP_000933736) (3).

(C) Eukaryotic (*Homo sapiens*, green) and bacterial (*Escherichia coli*, purple) pathways for conversion of D-glucosamine 6-P to *N*AG-1-P involve different enzymes and intermediates.

(D) Comparison of *E. coli* GlmU with *Rickettsia typhi* GlmU (AAU03917) illustrates that rickettsial GlmU proteins have lost the C-terminal acetyltransferase domain. Sequences were obtained from GenBank and aligned with MUSCLE (default parameters) (4). Domain colors above alignment correspond to description in panel B.

(E) PGN biosynthesis by *Rickettsia* spp. requires import of *N*AG-1-P, L-alanine (L-Ala) and *trans*,*trans*-farnesyl-PP (FPP) from the host. *N*AG-1-P is converted to UDP-*N*-acetyl-alpha-D-glucosamine (UDP-*N*AG) via the uridyltransferase GlmU_N, with the subsequent pathway to Lipid II (green arrows) highly conserved in *Rickettsia* genomes (see Fig. S10). Via alanine racemase (Alr), imported L-Ala is converted to D-Ala, which is incorporated into the stem peptide by D-alanine--D-alanine ligase A (DdlA). Imported FPP is converted via IspU and PgpB in to *di-trans*,*poly-cis*-undecaprenyl-P (orange arrows), which serves as the lipid carrier for Lipid I and

Lipid II (see Fig. 3 of the manuscript for more details). At least nine conserved enzymes participate in incorporation of PGN into the murine sacculus (red). The pathway for PGN recycling (purple arrows) initiates with subunit excision via lytic transglycosylases (Slt and RvhB1). Individual subunits are imported back to the cytoplasm via AmpG transporters, which are present in multiple divergent copies in each *Rickettsia* genome (5). The full Gram-negative bacterial PGN recycling pathway is shown (6), with enzymes lacking in *Rickettsia* genomes marked with red Xs. This minimal set of enzymes generates two recyclable products (purple ellipses), while the fate of the remaining degraded products (light green ellipses) is unknown.

(F) Lipopolysaccharide biosynthesis by *Rickettsia* spp. requires import of *N*AG-1-P, D-ribose 5-P and UDP-glucose from the host. GlmU_N converts imported *N*AG-1-P to UDP-*N*AG, which enters the Raetz pathway (7) for synthesis of Kdo_2-lipid A (light blue arrows). Imported D-ribose 5-P is converted to D-ribulose 5-P via ribose-5-phosphate isomerase B (RpiB), leading to synthesis of CMP-Kdo. A pathway hole (gray circle) indicates the absence of a phosphatase to convert 3-deoxy-D-manno-octulosonate 8-P to 3-deoxy-D-manno-octulosonate (Kdo) (see panels G-I). The incorporation of various fatty acids into the growing lipid A moiety is shown (pink arrows). Acyl chain incorporation into Lipid A follows the structure deduced for *R. typhi* (8). UDP-*N*AG is also ligated to *di-trans*,*poly-cis*-undecaprenyl-P via undecaprenyl-P alpha-*N*-acetylglucosaminyl 1-P transferase (WecA) (orange arrows), creating the lipid carrier for O-antigen (see Fig. 3 of the manuscript for more details). UDP-*N*-acetylglucosamine 2-epimerase (WecB) also utilizes UDP-*N*AG to generate UDP-*N*-acetyl-D-mannosamine (UDP-ManNAc). Imported UDP-glucose is converted to UDP-glucuronate (UDP-GlcA) and UDP-alpha-D-galactose (UDP-Gal) via UDP-glucose 6-dehydrogenase (Udg) and the epimerase/dehydratase FnlA, respectively. UDP-ManNAc, UDP-GlcA and UDP-Gal are predicted to be the main sugars used by peripheral enzymes (e.g., glycosyltransferases, glucosyltransferases, etc.) that polymerize O-antigen and extend the Kdo_2-lipid A acceptor.

(G) The pathway from D-Ribulose 5-P to CMP-Kdo is highly conserved across 2227 bacterial genomes (all prokaryotic genomes with an available Kdo_2-lipid A biosynthesis pathway at KEGG). The star depicts the pathway hole (no KdsC) in the *Rickettsia* CMP-Kdo pathway.

(H) Relative to the predominant "*E. coli*-like" pathway, the "*Rickettsia*-like" pathway for CMP-Kdo biosynthesis is far less common, occurring in 442 bacterial genomes (excluding *Rickettsia* genomes). The pathway hole (no KdsC) is noted with a red X.

(I) Taxonomic breakdown of bacterial species containing the "*Rickettsia*-like" CMP-Kdo biosynthesis pathway (442 total genomes). Genomes from intracellular species represent 41% of the total number of "*Rickettsia*-like" pathways.

REFERENCES

1. **Frohlich KM**, **Audia JP**. 2013. Dual mechanisms of metabolite acquisition by the obligate intracytosolic pathogen Rickettsia prowazekii reveal novel aspects of triose phosphate transport. J Bacteriol **195**:3752–60.
2. **Frohlich KM**, **Roberts RAW**, **Housley NA**, **Audia JP**. 2010. Rickettsia prowazekii uses an sn-glycerol-3-phosphate dehydrogenase and a novel dihydroxyacetone phosphate transport system to supply triose phosphate for phospholipid biosynthesis. J Bacteriol **192**:4281–8.
3. **Gehring AM**, **Lees WJ**, **Mindiola DJ**, **Walsh CT**, **Brown ED**. 1996. Acetyltransfer Precedes Uridylyltransfer in the Formation of UDP- *N* -acetylglucosamine in Separable Active Sites of the Bifunctional GlmU Protein of *Escherichia coli* †. Biochemistry **35**:579–585.
4. **Edgar RC**. 2004. MUSCLE: Multiple sequence alignment with high accuracy and high throughput. Nucleic Acids Res **32**:1792–1797.
5. **Gillespie JJ**, **Joardar V**, **Williams KP**, **Driscoll T**, **Hostetler JB**, **Nordberg E**, **Shukla M**, **Walenz B**, **Hill CA**, **Nene VM**, **Azad AF**, **Sobral BW**, **Caler E**. 2012. A Rickettsia

genome overrun by mobile genetic elements provides insight into the acquisition of genes characteristic of an obligate intracellular lifestyle. J Bacteriol **194**:376–94.

6. **Park JT**, **Uehara T**. 2008. How bacteria consume their own exoskeletons (turnover and recycling of cell wall peptidoglycan). Microbiol Mol Biol Rev **72**:211–27, table of contents.

7. **Whitfield C**, **Trent MS**. 2014. Biosynthesis and Export of Bacterial Lipopolysaccharides. Annu Rev Biochem **83**:99–128.

8. **Fodorová M**, **Vadovič P**, **Toman R**. 2011. Structural features of lipid A of Rickettsia typhi. Acta Virol **55**:31–44.

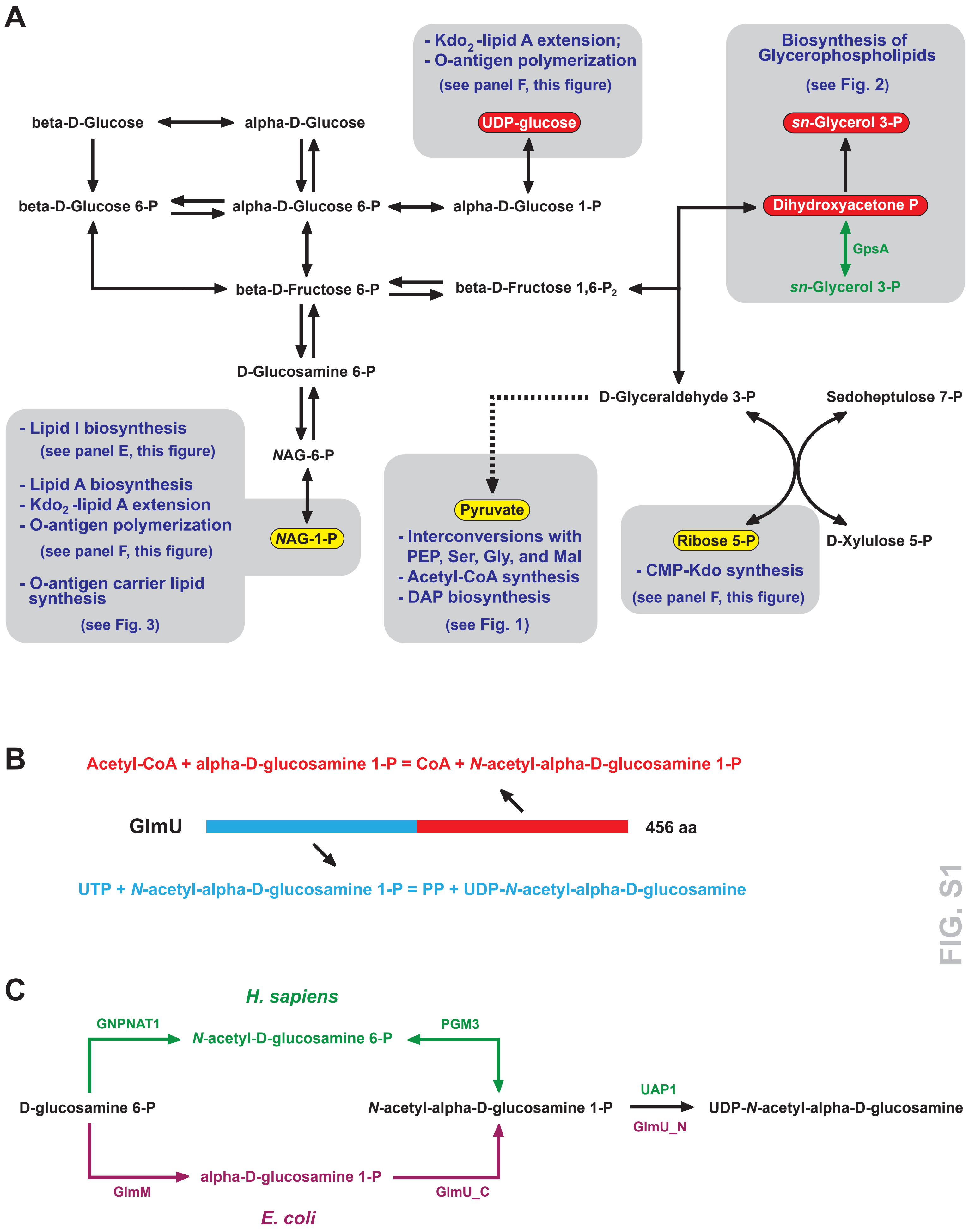
A
- Kdo2-lipid A extension;
- O-antigen polymerization
(see panel F, this figure)
UDP-glucose
Biosynthesis of Glycerophospholipids
(see Fig. 2)
sn-Glycerol 3-P
Dihydroxyacetone P
GpsA
sn-Glycerol 3-P
beta-D-Glucose
alpha-D-Glucose
beta-D-Glucose 6-P
alpha-D-Glucose 6-P
alpha-D-Glucose 1-P
beta-D-Fructose 6-P
beta-D-Fructose 1,6-P2
D-Glucosamine 6-P
D-Glyceraldehyde 3-P
Sedoheptulose 7-P
NAG-6-P
NAG-1-P
- Lipid I biosynthesis
(see panel E, this figure)
- Lipid A biosynthesis
- Kdo2 -lipid A extension
- O-antigen polymerization
(see panel F, this figure)
- O-antigen carrier lipid synthesis
(see Fig. 3)
Pyruvate
- Interconversions with PEP, Ser, Gly, and Mal
- Acetyl-CoA synthesis
- DAP biosynthesis
(see Fig. 1)
Ribose 5-P
- CMP-Kdo synthesis
(see panel F, this figure)
D-Xylulose 5-P
B
Acetyl-CoA + alpha-D-glucosamine 1-P = CoA + N-acetyl-alpha-D-glucosamine 1-P
GlmU
456 aa
UTP + N-acetyl-alpha-D-glucosamine 1-P = PP + UDP-N-acetyl-alpha-D-glucosamine
C
H. sapiens
GNPNAT1
N-acetyl-D-glucosamine 6-P
PGM3
D-glucosamine 6-P
N-acetyl-alpha-D-glucosamine 1-P
UAP1
GlmU_N
UDP-N-acetyl-alpha-D-glucosamine
alpha-D-glucosamine 1-P
GlmM
GlmU_C
E. coli

FIG. S1

D

```
E. coli   MLNNA-MSVVILAAGKGTRMYSDLPKVLHTLAGKAMVQHVIDAANELGAAHVHLVYGHGG   59
R. typhi  MLHNENYQIIILAAGKGTRMESDLPKVMHEVGGVPMLETVLKNALKI-THDVIIVY---S   56
          **:*   .::********** ******:* :.* .*:: *:. * :: :  * :**   .

E. coli   DLLKQALK--DDNLNWVLQAEQLGTGHAMQQAAPFFADDEDILMLYGDVPLISVETLQRL  117
R. typhi  EALKKYLTPYENMCRFVLQEEPKGTAHATHAAIDLIDQNKIILVLYGDHPLITPTLMYEL  116
          : **: *.  ::  .:*** *  **.** : *  :: ::: **:**** ***:    :  *

E. coli   RDAKPQGGIGLLTVKLD--DPTGYGRITRE-NGKVTGIVEHKDATDEQRQIQEINTGILI  174
R. typhi  IDYLSIINAALVTLSFERANPAQYGRIAIDKHGNFLEIIEYKNASEEEKKIKLCNSGIMA  176
          *  .  . .*:*:.::  :*: ****: : :*:.  *:*:*:*::*:.:*:  *:**:

E. coli   ANGADMKRWLAKLTNN--NAQGEYYITDIIALAYQEGREIVAVHPQRLSEVEGVNNRLQL  232
R. typhi  FSSGILNKYLPLFANNNTNCNQEIYLTEIVKICKNHGEKVSYLLSTDHDLIVGINTQSEL  236
          ... ::.:*. ::**  *.: * *:*:*: :. : * ::  : .   . : *:*.. :*

E. coli   SRLERVYQSEQAEKLLLAGVMLRDPARFDLRGTLTHGRDVEIDTNVIIEGNVTLGHRVKI  292
R. typhi  KEANNIFFQNKS------------------------------------------------  248
          .  :.:: .:::

E. coli   GTGCVIKNSVIGDDCEISPYTVVEDANLAAACTIGPFARLRPGAELLEGAHVGNFVEMKK  352
R. typhi  ------------------------------------------------------------  248

E. coli   ARLGKGSKAGHLTYLGDAEIGDNVNIGAGTITCNYDGANKFKTIIGDDVFVGSDTQLVAP  412
R. typhi  ------------------------------------------------------------  248

E. coli   VTVGKGATIAAGTTVTRNVGENALAISRVPQTQKEGWRRPVKKK  456
R. typhi  --------------------------------------------  248
```

E

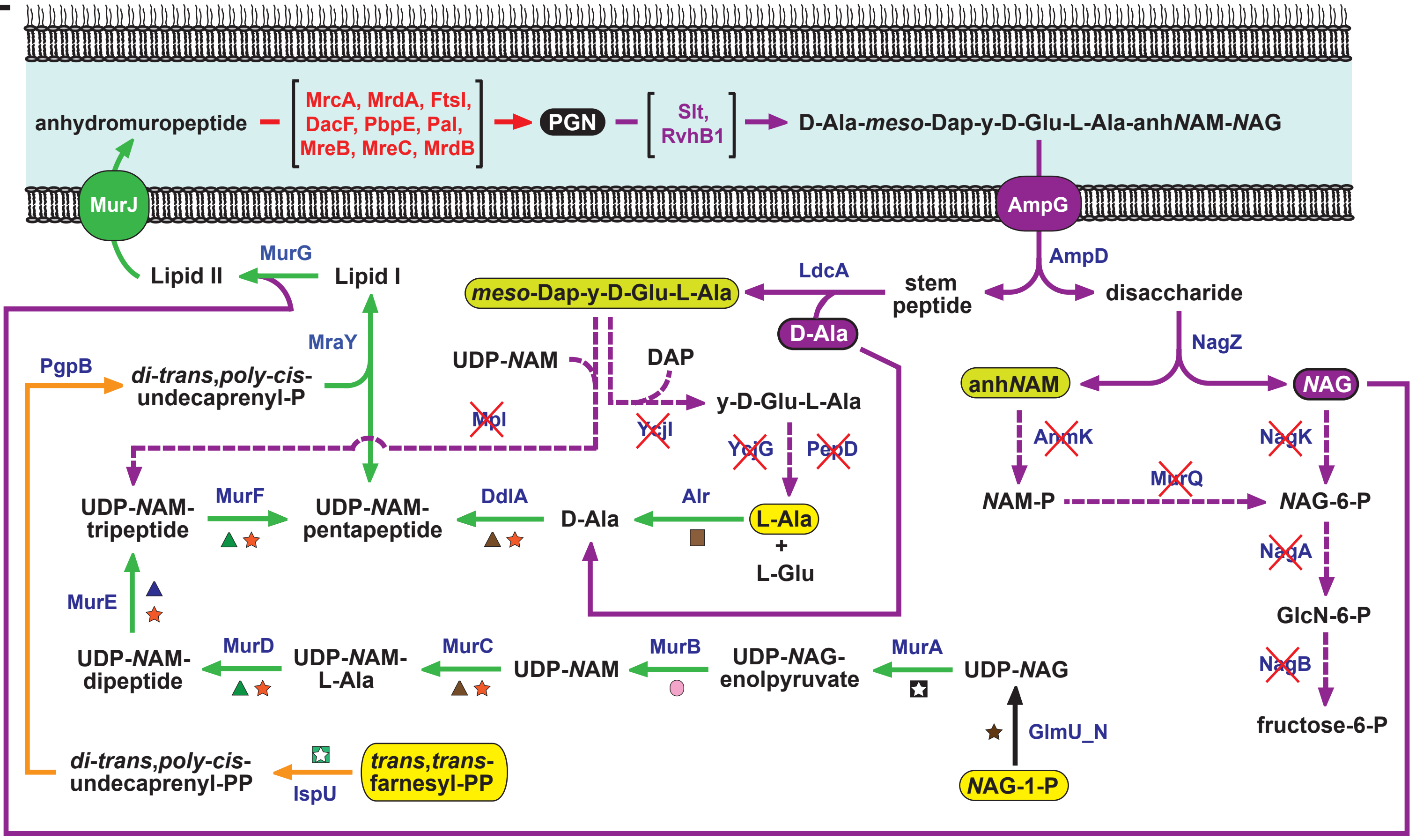

FIG. S1

F

FIG. S1

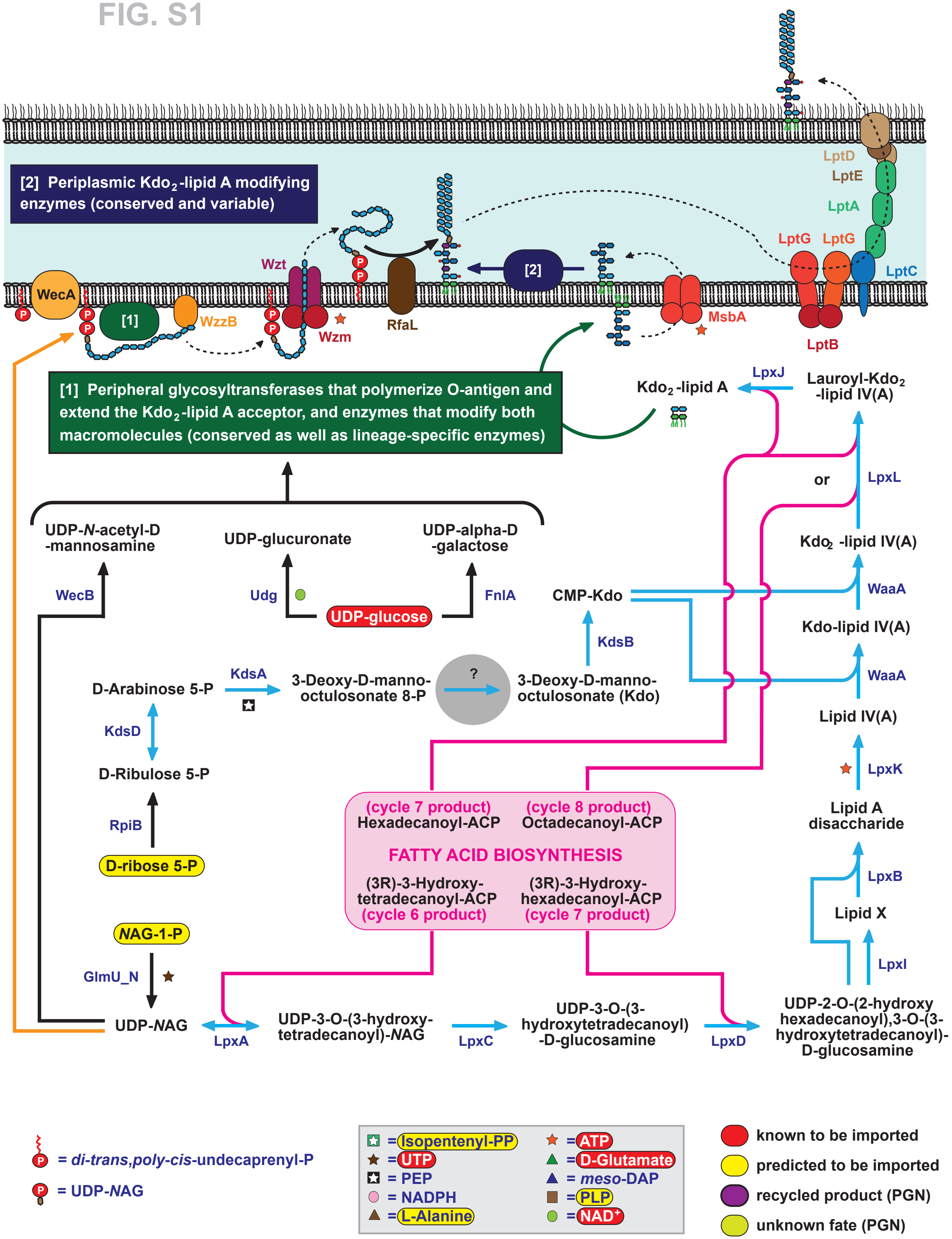

[2] Periplasmic Kdo$_2$-lipid A modifying enzymes (conserved and variable)
LptD
LptE
LptA
LptG
LptG
LptC
Wzt
WecA
[1]
WzzB
Wzm
RfaL
[2]
MsbA
LptB
[1] Peripheral glycosyltransferases that polymerize O-antigen and extend the Kdo$_2$-lipid A acceptor, and enzymes that modify both macromolecules (conserved as well as lineage-specific enzymes)
Kdo$_2$-lipid A
LpxJ
Lauroyl-Kdo$_2$-lipid IV(A)
or
LpxL
Kdo$_2$ -lipid IV(A)
WaaA
Kdo-lipid IV(A)
WaaA
Lipid IV(A)
LpxK
Lipid A disaccharide
LpxB
Lipid X
LpxI
UDP-*N*-acetyl-D-mannosamine
UDP-glucuronate
UDP-alpha-D-galactose
WecB
Udg
UDP-glucose
FnlA
CMP-Kdo
KdsB
D-Arabinose 5-P
KdsA
3-Deoxy-D-manno-octulosonate 8-P
?
3-Deoxy-D-manno-octulosonate (Kdo)
KdsD
D-Ribulose 5-P
RpiB
D-ribose 5-P
NAG-1-P
GlmU_N
UDP-*NAG*
(cycle 7 product) Hexadecanoyl-ACP
(cycle 8 product) Octadecanoyl-ACP
FATTY ACID BIOSYNTHESIS
(3R)-3-Hydroxy-tetradecanoyl-ACP (cycle 6 product)
(3R)-3-Hydroxy-hexadecanoyl-ACP (cycle 7 product)
LpxA
UDP-3-O-(3-hydroxy-tetradecanoyl)-*NAG*
LpxC
UDP-3-O-(3-hydroxytetradecanoyl)-D-glucosamine
LpxD
UDP-2-O-(2-hydroxy hexadecanoyl),3-O-(3-hydroxytetradecanoyl)-D-glucosamine
= *di-trans,poly-cis*-undecaprenyl-P
= UDP-*NAG*
= Isopentenyl-PP
= UTP
= PEP
= NADPH
= L-Alanine
= ATP
= D-Glutamate
= *meso*-DAP
= PLP
= NAD$^+$
known to be imported
predicted to be imported
recycled product (PGN)
unknown fate (PGN)

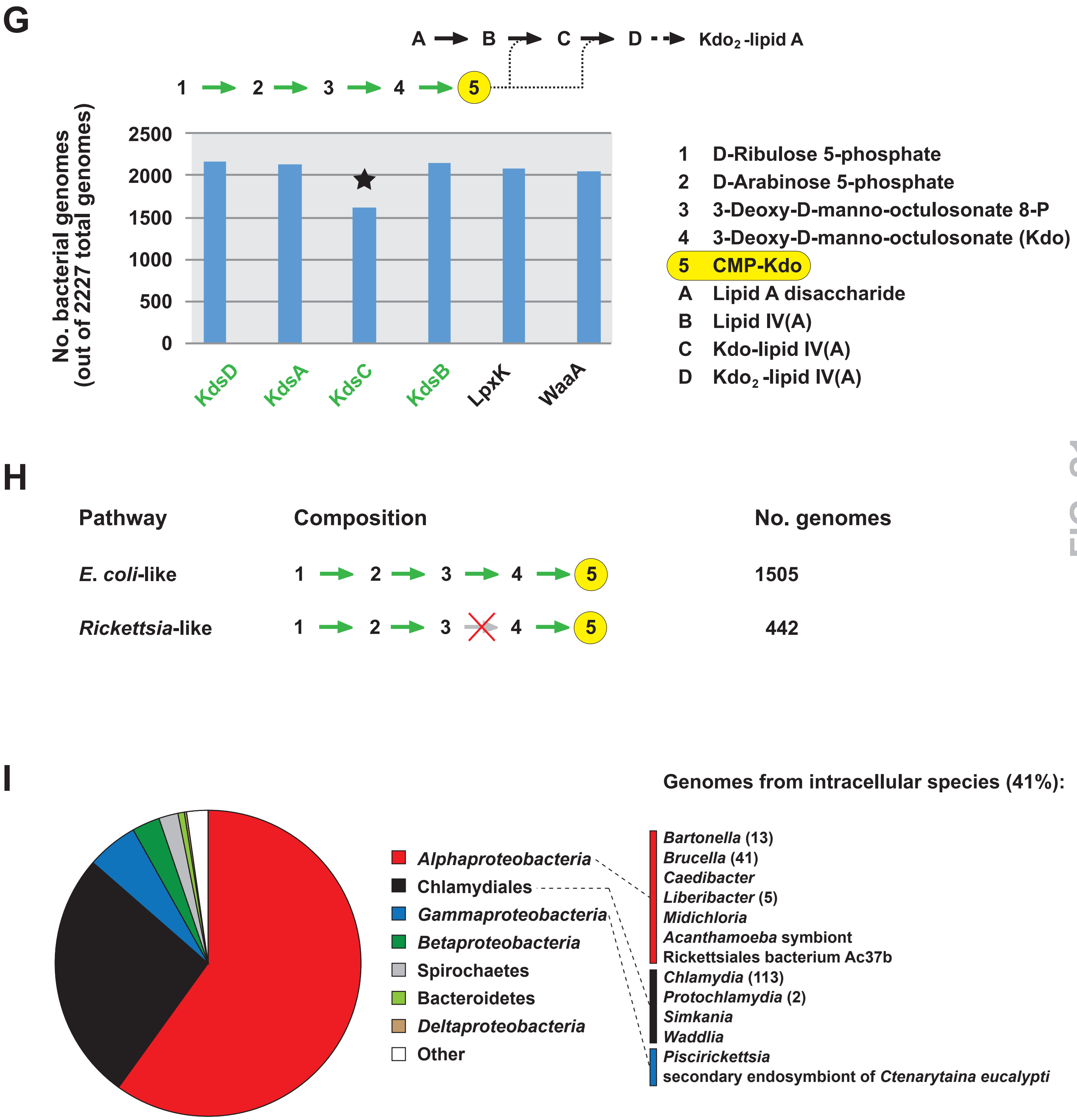
G
A → B → C → D → Kdo2-lipid A
1 → 2 → 3 → 4 → 5
No. bacterial genomes (out of 2227 total genomes)
2500
2000
1500
1000
500
0
KdsD
KdsA
KdsC
KdsB
LpxK
WaaA
1 D-Ribulose 5-phosphate
2 D-Arabinose 5-phosphate
3 3-Deoxy-D-manno-octulosonate 8-P
4 3-Deoxy-D-manno-octulosonate (Kdo)
5 CMP-Kdo
A Lipid A disaccharide
B Lipid IV(A)
C Kdo-lipid IV(A)
D Kdo2-lipid IV(A)
H
Pathway
Composition
No. genomes
E. coli-like
1 → 2 → 3 → 4 → 5
1505
Rickettsia-like
1 → 2 → 3 4 → 5
442
I
Genomes from intracellular species (41%):
Alphaproteobacteria
Chlamydiales
Gammaproteobacteria
Betaproteobacteria
Spirochaetes
Bacteroidetes
Deltaproteobacteria
Other
Bartonella (13)
Brucella (41)
Caedibacter
Liberibacter (5)
Midichloria
Acanthamoeba symbiont
Rickettsiales bacterium Ac37b
Chlamydia (113)
Protochlamydia (2)
Simkania
Waddlia
Piscirickettsia
secondary endosymbiont of Ctenarytaina eucalypti

FIG. S1
