# Supplementary material for: Wholly Rickettsia! Reconstructed Metabolic Profile of the Quintessential Bacterial Parasite of Eukaryotic Cells
Source: mBio. 2017 Sep 26;8(5):e00859-17. doi: 10.1128/mBio.00859-17 (PMC5615194; doi:10.1128/mBio.00859-17)
Supplement: FIG S2 [file mbo005173495sf2.pdf]

FIG S2. *Rickettsia* diaminopimelate biosynthesis contains a pathway hole for the conversion of *N*-Succinyl-L-2-amino-6-oxopimelate to *N*-Succinyl-LL-2,6-diaminopimelate.
(A) The pathway from L-Aspartate to *meso*-2,6-Diaminopimelate (DAP) has several characterized routes for conversion of 2,3,4,5-Tetrahydrodipicolinate to DAP (model at top, with different colors depicting alternative pathways). Bar graph illustrates the distribution of all DAP pathway enzymes across 3763 analyzed genomes (all prokaryotic genomes with an available DAP biosynthesis pathway at KEGG). Dashed gray lines connect conserved proximal and distal enzymes to the model above, with less conserved enzymes of the alternative pathways colored accordingly. The star depicts the pathway hole (no DapC or ArgD) in the *Rickettsia* DAP pathway.
(B) Relative to the predominant "*E. coli*-like" pathway, the "*Rickettsia*-like" pathway for DAP biosynthesis is far less common, occurring in 315 bacterial genomes (excluding *Rickettsia* genomes). The pathway hole (no DapC or ArgD) is noted with a red X.
(C) Taxonomic breakdown of bacterial species containing the "*Rickettsia*-like" DAP biosynthesis pathway (315 total genomes). Genomes from intracellular species represent 11% of the total number of "*Rickettsia*-like" pathways.
(D) Top blastp hits against the NCBI *Rickettsia* database (taxid:780) using the alternative DAP pathway enzymes as queries. This analysis shows that, despite lacking DapC or ArgD homologs, *Rickettsia* genomes do not contain any alternative enzymes of the other characterized DAP biosynthesis pathways.

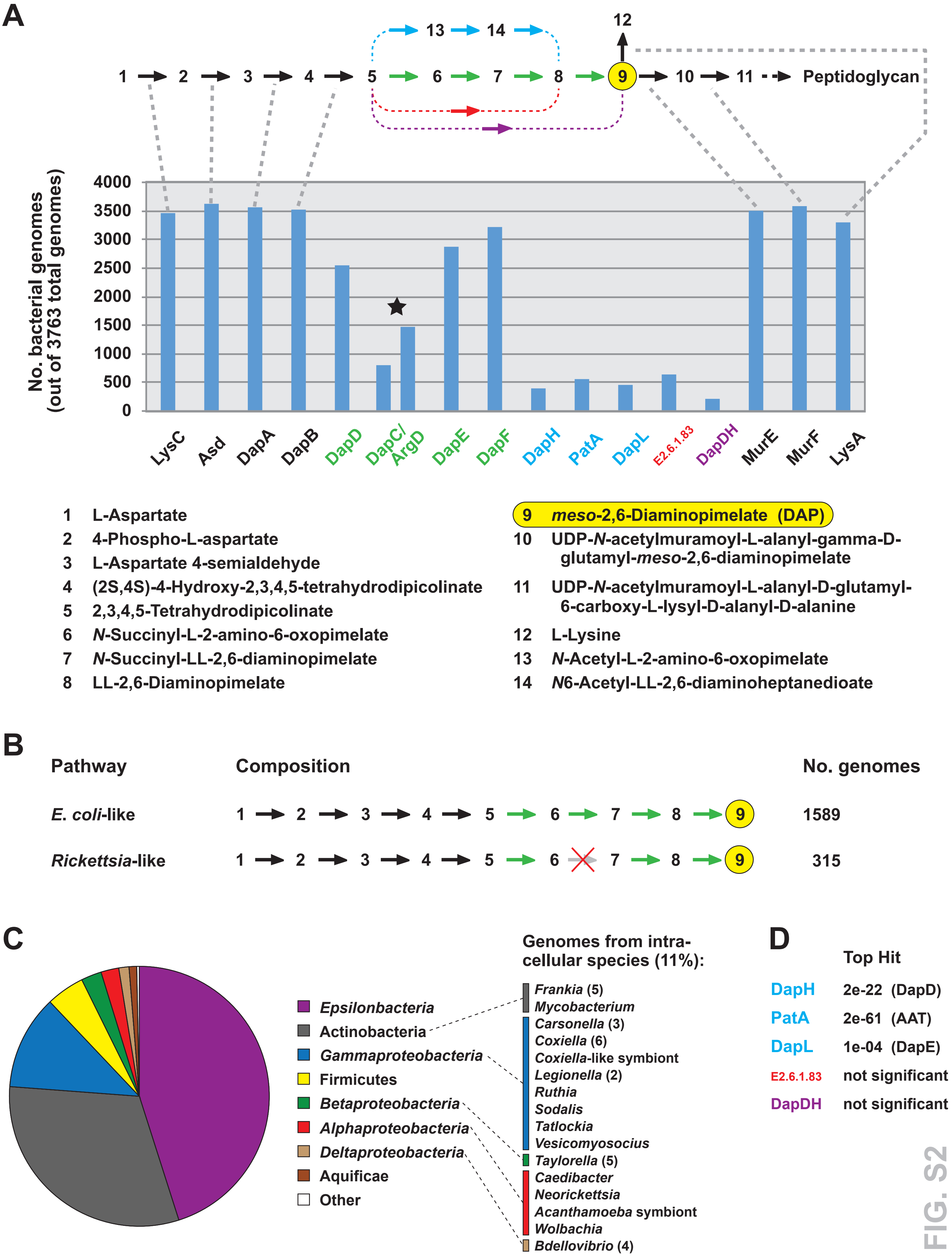
A
13
14
12
1
2
3
4
5
6
7
8
9
10
11
Peptidoglycan
No. bacterial genomes (out of 3763 total genomes)
4000
3500
3000
2500
2000
1500
1000
500
0
LysC
Asd
DapA
DapB
DapD
DapC/ArgD
DapE
DapF
DapH
PatA
DapL
E2.6.1.83
DapDH
MurE
MurF
LysA
1 L-Aspartate
2 4-Phospho-L-aspartate
3 L-Aspartate 4-semialdehyde
4 (2S,4S)-4-Hydroxy-2,3,4,5-tetrahydrodipicolinate
5 2,3,4,5-Tetrahydrodipicolinate
6 N-Succinyl-L-2-amino-6-oxopimelate
7 N-Succinyl-LL-2,6-diaminopimelate
8 LL-2,6-Diaminopimelate
9 meso-2,6-Diaminopimelate (DAP)
10 UDP-N-acetylmuramoyl-L-alanyl-gamma-D-glutamyl-meso-2,6-diaminopimelate
11 UDP-N-acetylmuramoyl-L-alanyl-D-glutamyl-6-carboxy-L-lysyl-D-alanyl-D-alanine
12 L-Lysine
13 N-Acetyl-L-2-amino-6-oxopimelate
14 N6-Acetyl-LL-2,6-diaminoheptanedioate
B
Pathway
Composition
No. genomes
E. coli-like
1 2 3 4 5 6 7 8 9
1589
Rickettsia-like
1 2 3 4 5 6 7 8 9
315
C
Epsilonbacteria
Actinobacteria
Gammaproteobacteria
Firmicutes
Betaproteobacteria
Alphaproteobacteria
Deltaproteobacteria
Aquificae
Other
Genomes from intra-cellular species (11%):
Frankia (5)
Mycobacterium
Carsonella (3)
Coxiella (6)
Coxiella-like symbiont
Legionella (2)
Ruthia
Sodalis
Tatlockia
Vesicomyosocius
Taylorella (5)
Caedibacter
Neorickettsia
Acanthamoeba symbiont
Wolbachia
Bdellovibrio (4)
D
Top Hit
DapH 2e-22 (DapD)
PatA 2e-61 (AAT)
DapL 1e-04 (DapE)
E2.6.1.83 not significant
DapDH not significant

FIG. S2
