# Supplementary material for: Wholly Rickettsia! Reconstructed Metabolic Profile of the Quintessential Bacterial Parasite of Eukaryotic Cells
Source: mBio. 2017 Sep 26;8(5):e00859-17. doi: 10.1128/mBio.00859-17 (PMC5615194; doi:10.1128/mBio.00859-17)
Supplement: FIG S3 [file mbo005173495sf3.pdf]

FIG S3. *Rickettsia* biosynthesis of glycerophospholipids contains a pathway hole for the conversion of *sn*-glycerol 3-P to lysophosphatidic acid.

(A) The pathway from dihydroxyacetone phosphate to CDP-diacylglycerol (CDP-DG) contains two characterized enzymes that generate lysophosphatidic acid (LPA) from *sn*-glycerol 3-phosphate (G3P): the G3P acyltransferases PlsB and PlsY, both of which can utilize either acyl-CoA or acyl-ACP as the fatty acyl donor. The phosphate acyltransferase PlsX converts acyl-ACP to acyl-PO_4, which is also a donor for PlsY (1, 2), and both enzymes occur exclusively in bacteria (3). The bar graph indicates that the LPA-generating acyltransferases are variably-encoded across 3830 bacterial genomes, while the remaining enzymes of the pathway are highly conserved. The star depicts the pathway hole (no PlsB, PlsX or PlsY) in the *Rickettsia* glycerophospholipid biosynthesis pathway.

(B) The predominant bacterial glycerophospholipid biosynthesis pathway utilizes the PlsX/Y acyltransferases to generate LPA, with other pathways far less common. The "*Rickettsia*-like" pathway for generating LPA (i.e., sans any of the characterized G3P acyltransferases) occurs in 433 bacterial genomes (excluding *Rickettsia* genomes). The pathway hole (no PlsB, PlsX or PlsY) is noted with red Xs.

(C) Taxonomic breakdown of bacterial species containing the "*Rickettsia*-like" glycerophospholipid biosynthesis pathway (433 total genomes). Genomes from intracellular species represent 3% of the total number of "*Rickettsia*-like" pathways.

REFERENCES

1. **Lu Y-J**, **Zhang Y-M**, **Grimes KD**, **Qi J**, **Lee RE**, **Rock CO**. 2006. Acyl-phosphates initiate membrane phospholipid synthesis in Gram-positive pathogens. Mol Cell **23**:765–72.

2. **Lu Y-J**, **Zhang F**, **Grimes KD**, **Lee RE**, **Rock CO**. 2007. Topology and active site of PlsY: the bacterial acylphosphate:glycerol-3-phosphate acyltransferase. J Biol Chem **282**:11339–46.

3. **Yao J**, **Rock CO**. 2013. Phosphatidic acid synthesis in bacteria. Biochim Biophys Acta **1831**:495–502.

A

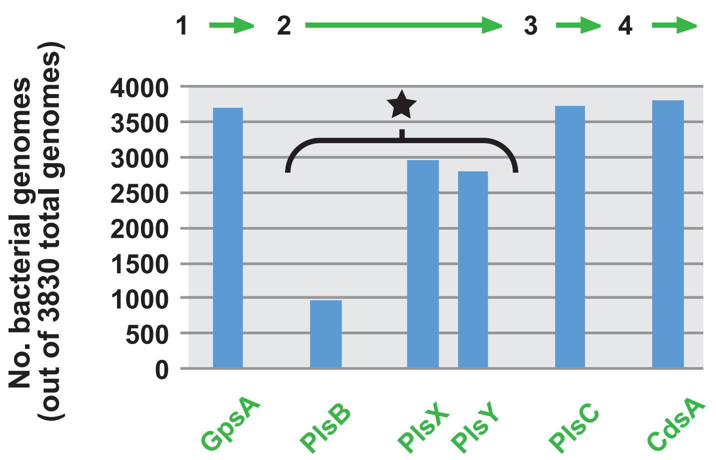

1 Dihydroxyacetone phosphate
2 *sn*-Glycerol 3-phosphate
3 Lysophosphatidic acid
4 Phosphatidic acid
5 CDP-diacylglycerol

B

Pathway	Composition (enzymes)	No. genomes
E. coli-like	GpsA, PlsB, PlsX and PlsY, PlsC, CdsA	650
Predominant	GpsA, ~~PlsB~~, PlsX and PlsY, PlsC, CdsA	1938
PlsB + PlsX	GpsA, PlsB, PlsX and ~~PlsY~~, PlsC, CdsA	650
PlsB + PlsY	GpsA, PlsB, ~~PlsX~~ and PlsY, PlsC, CdsA	27
PlsB only	GpsA, PlsB, ~~PlsX~~ and ~~PlsY~~, PlsC, CdsA	240
PlsX only	GpsA, ~~PlsB~~, PlsX and ~~PlsY~~, PlsC, CdsA	190
PlsY only	GpsA, ~~PlsB~~, ~~PlsX~~ and PlsY, PlsC, CdsA	31
Rickettsia-like	GpsA, ~~PlsB~~, ~~PlsX~~ and ~~PlsY~~, PlsC, CdsA	433

C

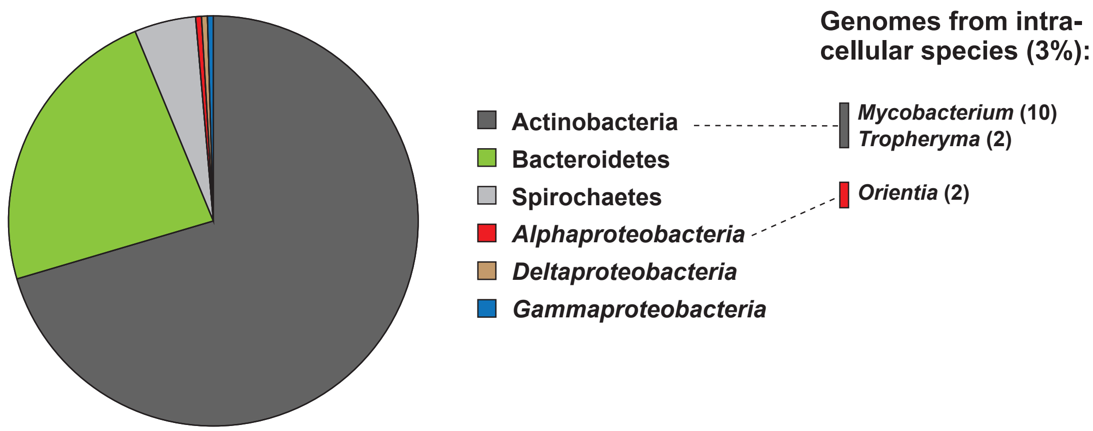

FIG. S3
