# Supplementary material for: Wholly Rickettsia! Reconstructed Metabolic Profile of the Quintessential Bacterial Parasite of Eukaryotic Cells
Source: mBio. 2017 Sep 26;8(5):e00859-17. doi: 10.1128/mBio.00859-17 (PMC5615194; doi:10.1128/mBio.00859-17)
Supplement: FIG S4 [file mbo005173495sf4.pdf]

FIG S4. Pathways for *Rickettsia* terpenoid and ubiquinone biosynthesis contain holes that are not common in other bacterial genomes.

(A) Bacteria utilize a variety of enzymes (e.g., farnesyl diphosphate synthase, IspA; or short chain isoprenyl diphosphate synthase, IdsA; or geranylgeranyl pyrophosphate synthase, GGPS) to generate geranyl diphosphate (GPP) and *trans*,*trans*-farnesyl diphosphate (FPP). The precursor isoprenes isopentenyl diphosphate (IPP) and dimethylallyl diphosphate (DMAPP), generated by the Mevalonate or MEP/DOXP pathway, are critical for these reactions, with DMAPP also necessary to provide dimethylallyl phosphate for the ubiquinone (CoQ_8) pathway. NOTE: the schema only shows IPP and DMAPP generated via the MEP/DOXP pathway, which is the predominant route for terpenoid synthesis in bacteria. Bar graph illustrates the distribution of these enzymes across 3763 analyzed genomes (all bacterial genomes with an available terpenoid biosynthesis pathway at KEGG). Also included is 4-hydroxy-3-methylbut-2-enyl diphosphate reductase (IspH), the terminal enzyme of the MEP/DOXP pathway, and the enzymes necessary to generate the lipid carriers for cell envelope glycoconjugates (undecaprenyl diphosphate synthase, IspU) and CoQ_8 (octaprenyl-diphosphate synthase, IspB). The star depicts the pathway hole (no IspA or IdsA or GGPS) in the *Rickettsia* terpenoid biosynthesis pathway.

(B) Relative to the "*E. coli*-like" pathway, the "*Rickettsia*-like" pathway for terpenoid biosynthesis is far less common, occurring in only 37 bacterial genomes (excluding *Rickettsia* genomes). The pathway hole (no IspA or IdsA or GGPS) is noted with red Xs (the lack of IspH is not considered a pathway hole since the entire MEP/DOXP pathway is absent).

(C) Taxonomic breakdown of bacterial species containing the "*Rickettsia*-like" terpenoid biosynthesis pathway (37 total genomes). Genomes from intracellular species represent 24% of the total number of "*Rickettsia*-like" pathways (all species of *Blattabacterium*, obligate mutualistic endosymbionts of cockroaches).

(D) Bacteria synthesize ubiquinone from chorismate and an *all*-*trans*-polyprenyl diphosphate (octaprenyl diphosphate (ODP) in the case of *E. coli* and *Rickettsia* species). Bar graph illustrates

the distribution of the enzymes of the ubiquinone pathway across 3195 analyzed genomes (all bacterial genomes with an available ubiquinone biosynthesis pathway at KEGG). NOTE: many bacteria do not synthesize ubiquinone, but instead generate other quinones (only UbiE is found across all pathways). The star depicts the pathway hole (no UbiI) in the *Rickettsia* CoQ_8 biosynthesis pathway.

(E) Relative to the "*E*. *coli*-like" pathway, the "*Rickettsia*-like" pathway for CoQ_8 biosynthesis is far less common, occurring in only 74 bacterial genomes (excluding *Rickettsia* genomes). The pathway hole (no UbiI) is noted with red Xs (the lack of UbiC is not considered a pathway hole since *Rickettsia* species do synthesize chorismate and thus cannot generate 4-hydroxybenzoate).

(F) Taxonomic breakdown of bacterial species containing the "*Rickettsia*-like" CoQ_8 biosynthesis pathway (74 total genomes). Genomes from intracellular species represent 32% of the total number of "*Rickettsia*-like" pathways.

(G) Phylogenomics analysis (across 84 genomes) of the *Rickettsia* terpenoid backbone and CoQ_8 biosynthesis pathways. See manuscript for the position of enzymes within their respective pathways (Fig. 3). Yellow highlighting indicates that the most basal lineage of SFG rickettsiae (*R*. *tamurae*, *R*. *monacensis*, REIP, and *R*. *buchneri* strains) lack *idi*, and thus must acquire DMAPP from the host.

A

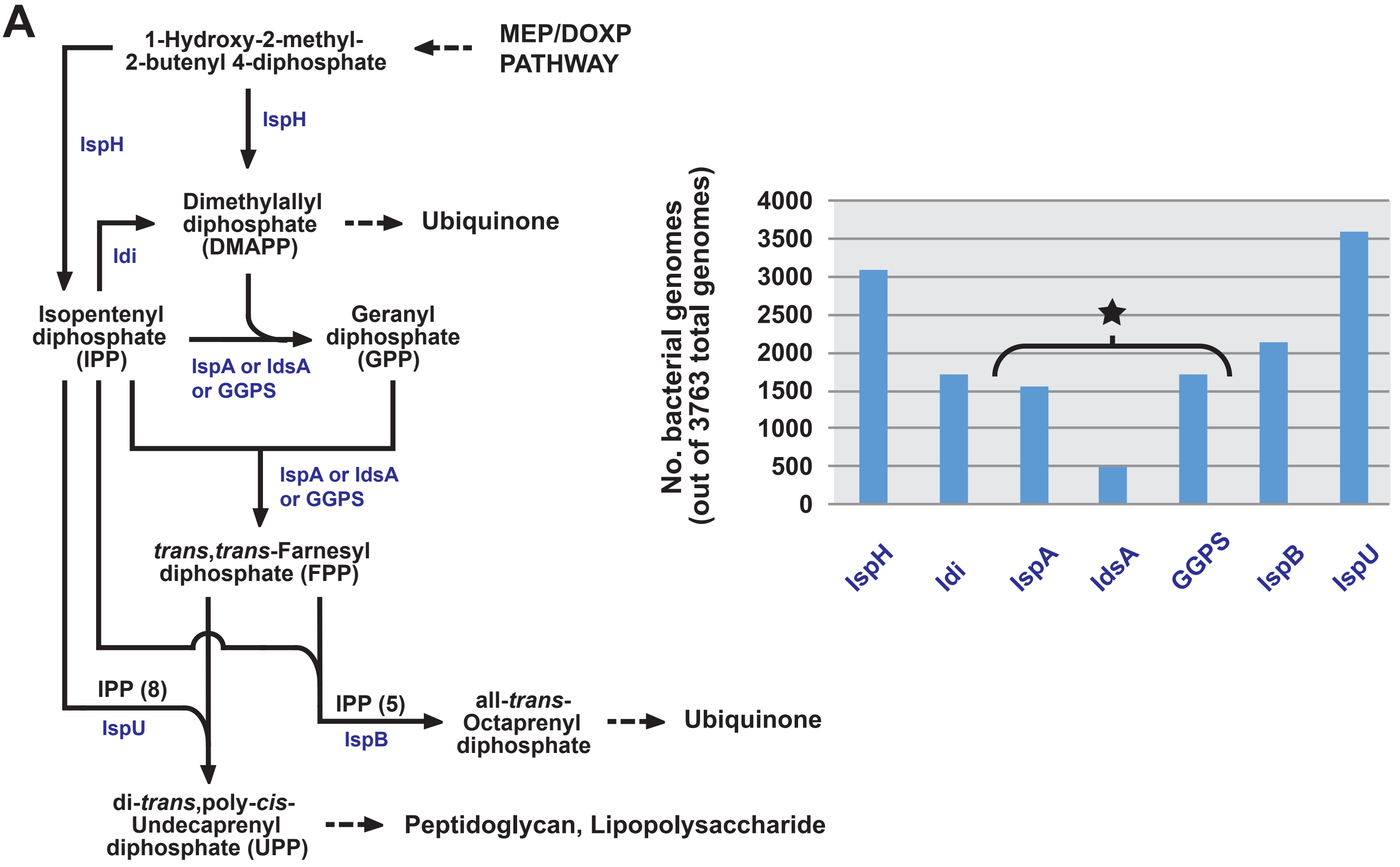

B

Pathway	Composition (enzymes)	No. genomes
E. coli-like	IspH, Idi, IspA or IdsA or GGPS, IspB, IspU	445
Rickettsia-like	IspH, Idi, ~~IspA~~ or ~~IdsA~~ or ~~GGPS~~, IspB, IspU	37

C

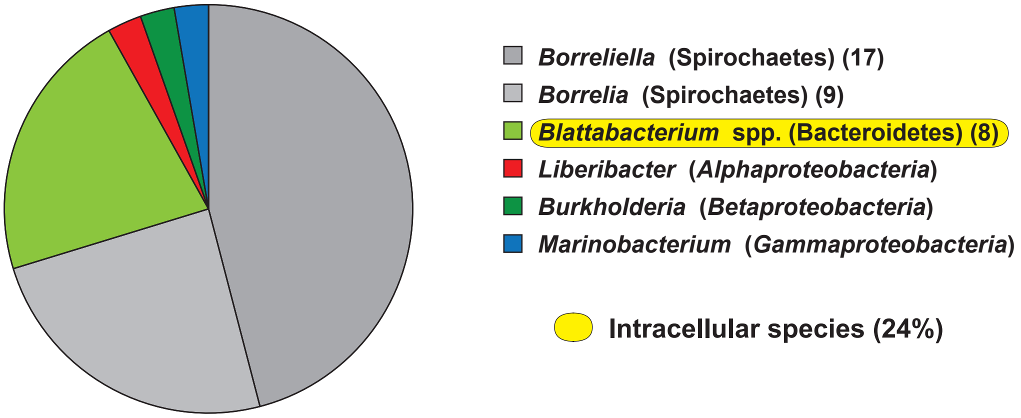

FIG. S4

D

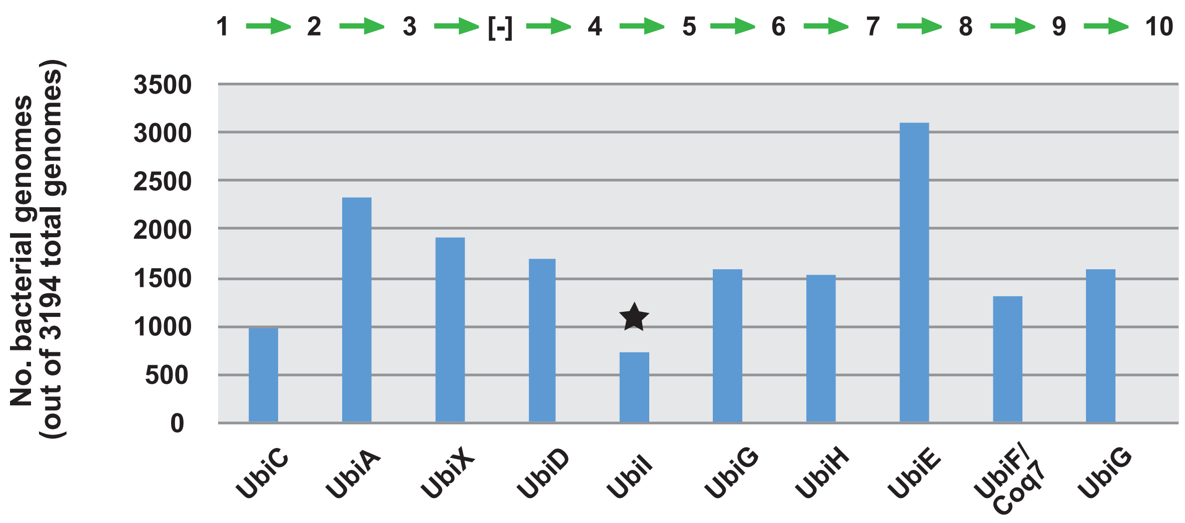

1 Chorismate
2 4-Hydroxybenzoate
3 3-Octaprenyl-4-hydroxybenzoate
\- Prenylated $FMNH_2$
4 2-Octaprenylphenol
5 2-Octaprenyl-6-hydroxyphenol
6 2-Octaprenyl-6-methoxyphenol
7 2-Octaprenyl-6-methoxy-1,4-benzoquinone
8 2-Octaprenyl-3-methyl-6-methoxy-1,4-benzoquinone
9 2-Octaprenyl-3-methyl-5-hydroxy-6-methoxy-1,4-benzoquinone
10 Ubiquinone-8 (Coenzyme Q_8)

E

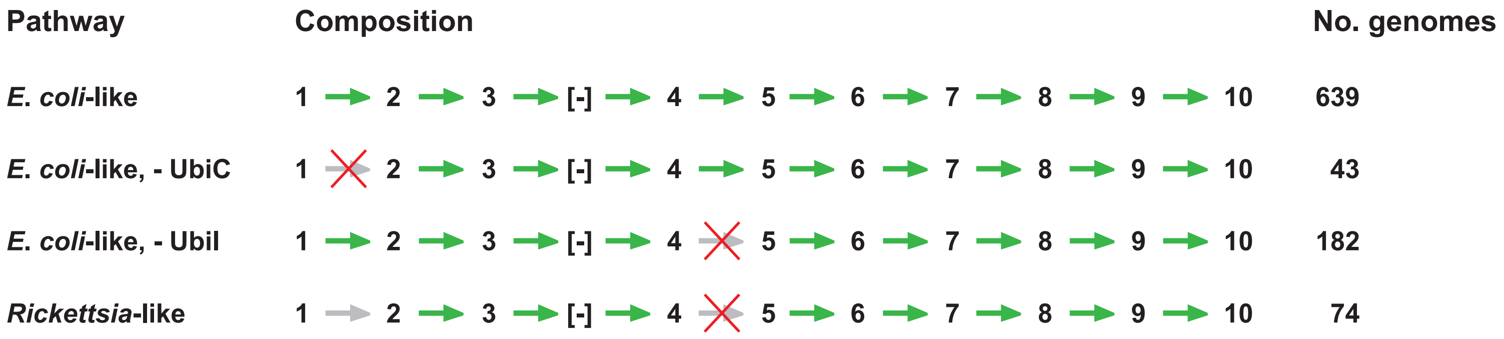

F

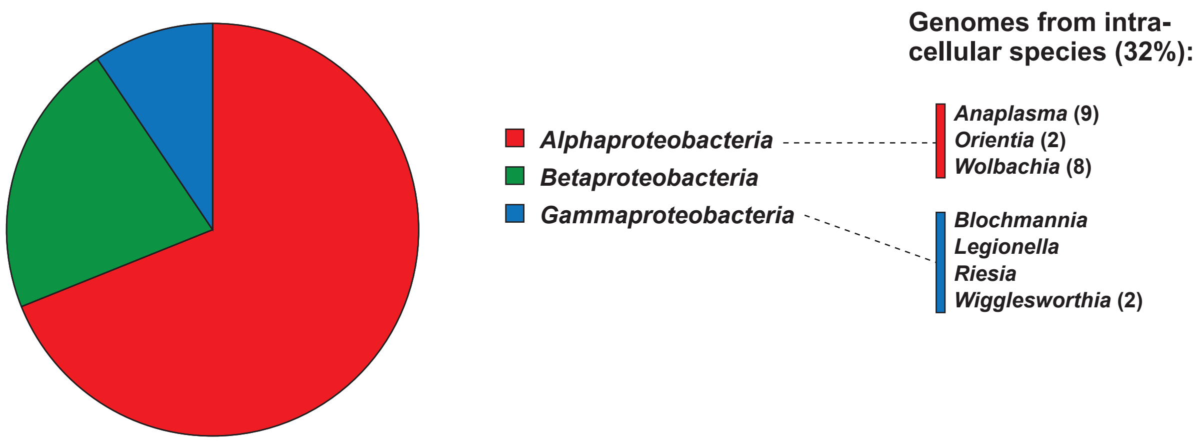

FIG. S4

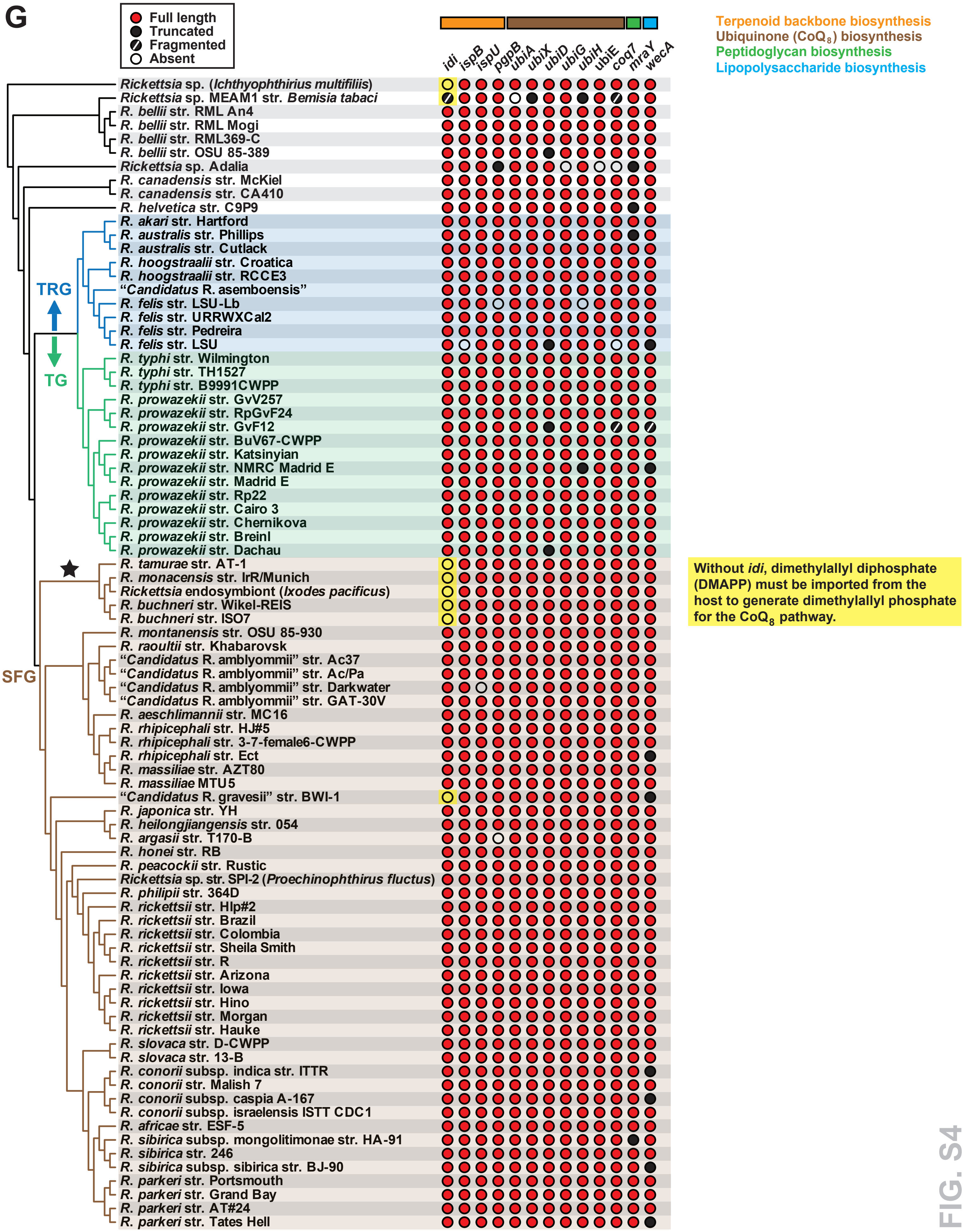

G
Full length
Truncated
Fragmented
Absent
Terpenoid backbone biosynthesis
Ubiquinone (CoQ_8) biosynthesis
Peptidoglycan biosynthesis
Lipopolysaccharide biosynthesis
idi
ispB
ispU
pgpB
ubiA
ubiX
ubiD
ubiG
ubiH
ubiE
coq7
mraY
wecA
TRG
TG
SFG
Rickettsia sp. (Ichthyophthirius multifiliis)
Rickettsia sp. MEAM1 str. Bemisia tabaci
R. bellii str. RML An4
R. bellii str. RML Mogi
R. bellii str. RML369-C
R. bellii str. OSU 85-389
Rickettsia sp. Adalia
R. canadensis str. McKiel
R. canadensis str. CA410
R. helvetica str. C9P9
R. akari str. Hartford
R. australis str. Phillips
R. australis str. Cutlack
R. hoogstraalii str. Croatica
R. hoogstraalii str. RCCE3
“Candidatus R. asemboensis”
R. felis str. LSU-Lb
R. felis str. URRWXCal2
R. felis str. Pedreira
R. felis str. LSU
R. typhi str. Wilmington
R. typhi str. TH1527
R. typhi str. B9991CWPP
R. prowazekii str. GvV257
R. prowazekii str. RpGvF24
R. prowazekii str. GvF12
R. prowazekii str. BuV67-CWPP
R. prowazekii str. Katsinyian
R. prowazekii str. NMRC Madrid E
R. prowazekii str. Madrid E
R. prowazekii str. Rp22
R. prowazekii str. Cairo 3
R. prowazekii str. Chernikova
R. prowazekii str. Breinl
R. prowazekii str. Dachau
R. tamurae str. AT-1
R. monacensis str. IrR/Munich
Rickettsia endosymbiont (Ixodes pacificus)
R. buchneri str. Wikel-REIS
R. buchneri str. ISO7
R. montanensis str. OSU 85-930
R. raoultii str. Khabarovsk
“Candidatus R. amblyommii” str. Ac37
“Candidatus R. amblyommii” str. Ac/Pa
“Candidatus R. amblyommii” str. Darkwater
“Candidatus R. amblyommii” str. GAT-30V
R. aeschlimannii str. MC16
R. rhipicephali str. HJ#5
R. rhipicephali str. 3-7-female6-CWPP
R. rhipicephali str. Ect
R. massiliae str. AZT80
R. massiliae MTU5
“Candidatus R. gravesii” str. BWI-1
R. japonica str. YH
R. heilongjiangensis str. 054
R. argasii str. T170-B
R. honei str. RB
R. peacockii str. Rustic
Rickettsia sp. str. SPI-2 (Proechinophthirus fluctus)
R. philipii str. 364D
R. rickettsii str. Hlp#2
R. rickettsii str. Brazil
R. rickettsii str. Colombia
R. rickettsii str. Sheila Smith
R. rickettsii str. R
R. rickettsii str. Arizona
R. rickettsii str. Iowa
R. rickettsii str. Hino
R. rickettsii str. Morgan
R. rickettsii str. Hauke
R. slovaca str. D-CWPP
R. slovaca str. 13-B
R. conorii subsp. indica str. ITTR
R. conorii str. Malish 7
R. conorii subsp. caspia A-167
R. conorii subsp. israelensis ISTT CDC1
R. africae str. ESF-5
R. sibirica subsp. mongolitimonae str. HA-91
R. sibirica str. 246
R. sibirica subsp. sibirica str. BJ-90
R. parkeri str. Portsmouth
R. parkeri str. Grand Bay
R. parkeri str. AT#24
R. parkeri str. Tates Hell
Without idi, dimethylallyl diphosphate (DMAPP) must be imported from the host to generate dimethylallyl phosphate for the CoQ_8 pathway.

FIG. S4
