# Supplementary material for: Wholly Rickettsia! Reconstructed Metabolic Profile of the Quintessential Bacterial Parasite of Eukaryotic Cells
Source: mBio. 2017 Sep 26;8(5):e00859-17. doi: 10.1128/mBio.00859-17 (PMC5615194; doi:10.1128/mBio.00859-17)
Supplement: FIG S5 [file mbo005173495sf5.pdf]

FIG S5. *Rickettsia* species utilize 7,8-dihydroneopterin 3'-triphosphate for queuosine biosynthesis and import host tetrahydrofolate for one-carbon transfer reactions by folate. In the classical folate biosynthesis pathway, the conversion of 7,8-dihydroneopterin to 6-hydroxymethyl-7,8-dihydropteridin (HMDHP) is carried out by dihydropteridin aldolase (FolB). In the “FolB bypass”, PTPS-III can generate HMDHP directly from 7,8-dihydroneopterin 3'-triphosphate (DHN-P_3) (see Fig. 4 of the manuscript). PTPS-III is a variant of 6-pyruvoyl tetrahydrobiopterin synthase (PTPS), a widespread group of enzymes functioning mainly in biopterin (1, 2) and queuosine (3, 4) synthesis. The PTPS active site contains a Cys; however, the active site of PTPS-III has Cys either replaced or accompanied by Glu, a specialization imparting functional replacement of FolB *in vivo* (5). For *Rickettsia* species, a previous report suggested that a conserved protein (e.g., NCBI accession no. KJW03529 from *Rickettsia* endosymbiont of *Ixodes pacificus*) could substitute for a missing FolB enzyme, completing a pathway for folate biosynthesis in those species containing FolE, FolK/P, FolC, and FolA (6). The alternative hypothesis identifies KJW03529 as 6-carboxy-5,6,7,8-tetrahydropterin synthase (QueD), an enzyme that converts 7,8-dihydroneopterin 3'-triphosphate to 6-carboxy-5,6,7,8-tetra-hydropterin, the first committed step in the synthesis of queuosine, a modified nucleoside that is present in certain tRNAs in bacteria and eukaryotes.

(A) Analysis of the active sites of select QueD and PTPS-III sequences indicates that the *Rickettsia* endosymbiont of *Ixodes pacificus* protein KJW03529 (boxed) is QueD and not PTPS-III. Sequences were obtained from Uniprot and aligned with MUSCLE (default parameters) (7). Yellow highlighting depicts the active sites, with asterisks under the alignment showing invariant residues. Sequence names are colored according to taxonomy (see inset).

(B) The active site of *Rickettsia* PTPS-like proteins is highly conserved, indicating that all *Rickettsia* genomes encode QueD enzymes involved in queuosine synthesis (instead of PTPS-III enzymes involved in folate synthesis). Only non-redundant proteins (n = 36) from the QueD ortholog group were analyzed. Sequence logo was generated using WEBLOGO v.3.3 (8).

(C) Like other enzymes of the folate biosynthesis pathway, FolB genes from most *Rickettsia* species have undergone pseudogenization. In this analysis, phylogeny estimations were compared between the shorter FolB proteins and the larger FolC proteins from diverse bacterial lineages. Despite the difficulty in estimating phylogeny on small sequences (limited informative sites to support estimated branching patterns), the FolB and FolC trees are largely congruent. For *Rickettsia rhipicephali* str. 3-7-female6-CWPP, one of only three *Rickettsia* genomes harboring a complete *folB* gene, both its FolB and FolC proteins are placed in the same position in both phylogeny estimations. This indicates that these FolB proteins are remnants of a once functional folate pathway encoded in *Rickettsia* genomes, and not recent products of lateral gene transfer. All sequences were retrieved from NCBI or Uniprot and aligned with MUSCLE (default parameters) (7). Phylogenies were estimated under maximum likelihood (ML) using RAxML v.7.2.8 (9), implementing a gamma model of rate heterogeneity and estimation of the proportion of invariable sites. Two separate analyses for each dataset (FolB and FolC) employed the WAG or LG amino acid substitution models, resulting in four total ML-based phylogeny estimations (the trees using the LG models are shown, and are highly similar to those using the WAG models). Branch support was assessed with 1000 bootstrap pseudoreplications.

(D) Phylogenomics analysis (across 84 genomes) of the *Rickettsia* queuosine and folate biosynthesis pathways, and enzymes participating in the reactions within the one carbon pool by folate. All of the enzymes are shown within their respective pathways (see Fig. 4 of the manuscript). NOTE: no single *Rickettsia* genome encodes a complete pathway for *de novo* folate biosynthesis (yellow highlighting), while the queuosine biosynthesis and one carbon pool by folate pathways are highly conserved.

REFERENCES

1. **Forrest HS**, **Baalen C V**. 1970. Microbiology of Unconjugated Pteridines. Annu Rev Microbiol **24**:91–108.

2. **Kong JS**, **Kang J-Y**, **Kim HL**, **Kwon O-S**, **Lee KH**, **Park YS**. 2006. 6-Pyruvoyltetrahydropterin synthase orthologs of either a single or dual domain structure are responsible for tetrahydrobiopterin synthesis in bacteria. FEBS Lett **580**:4900–4.
3. **McCarty RM**, **Somogyi A**, **Bandarian V**. 2009. *Escherichia coli* QueD Is a 6-Carboxy-5,6,7,8-tetrahydropterin Synthase †. Biochemistry **48**:2301–2303.
4. **Reader JS**, **Metzgar D**, **Schimmel P**, **de Crécy-Lagard V**. 2003. Identification of Four Genes Necessary for Biosynthesis of the Modified Nucleoside Queuosine. J Biol Chem **279**:6280–6285.
5. **Pribat A**, **Jeanguenin L**, **Lara-Núñez A**, **Ziemak MJ**, **Hyde JE**, **de Crécy-Lagard V**, **Hanson AD**. 2009. 6-pyruvoyltetrahydropterin synthase paralogs replace the folate synthesis enzyme dihydroneopterin aldolase in diverse bacteria. J Bacteriol **191**:4158–65.
6. **Hunter DJ**, **Torkelson JL**, **Bodnar J**, **Mortazavi B**, **Laurent T**, **Deason J**, **Thephavongsa K**, **Zhong J**. 2015. The Rickettsia Endosymbiont of Ixodes pacificus Contains All the Genes of De Novo Folate Biosynthesis. PLoS One **10**:e0144552.
7. **Edgar RC**. 2004. MUSCLE: Multiple sequence alignment with high accuracy and high throughput. Nucleic Acids Res **32**:1792–1797.
8. **Crooks GE**, **Hon G**, **Chandonia J-M**, **Brenner SE**. 2004. WebLogo: a sequence logo generator. Genome Res **14**:1188–90.
9. **Stamatakis A**. 2014. RAxML version 8: A tool for phylogenetic analysis and post-analysis of large phylogenies. Bioinformatics **30**:1312–1313.

A

Group	Organism [accession]		Sequence		
QueD	*Escherichia coli* str. K12 [P65870]	[15]	HRLPHVPEGHKCGRLHGH	[.88]	121
	Salmonella enterica subsp. enterica serovar Ouakam [AKQ35539]	[21]	HRLPHVPEGHKCGRLHGH	[.88]	127
	Sinorhizobium americanum [WP_037377663]	[12]	HRLPNVPSTHKCHRMHGH	[.79]	118
	Rickettsia endosymbiont of *Ixodes pacificus* [KJW03529]	[13]	HRIIG--HQNKCQFLHGH	[109]	138
	"*Candidatus* Midichloria mitochondrii" str. IricVA [AEI88702]	[13]	HRVVG--HQGKCYKLHGH	[112]	141
	Bordetella petrii [WP_028355890]	[13]	HRIPD--HRSQCRNLHGH	[118]	147
	Azoarcus olearius [WP_065341117]	[12]	HRIPD--HASQCRHLHGH	[123]	151
	Prevotella bivia [WP_061315274]	[15]	HRVVG--HENKCKHLHGH	[110]	141
	Porphyromonas gingivicanis [WP_025843504]	[22]	HRVVG--HEHKCRHLHGH	[114]	152
	Parachlamydia acanthamoebae str. Hall's coccus [EFB41517]	[15]	HRVMN--HENKCATAHGH	[112]	143
PTPS-III	gamma proteobacterium IMCC3088 [F3KYM3]	[19]	HFTIF--SATDRERLHGH	[127]	162
	Pseudohaliea rubra DSM 19751 [A0A095X331]	[22]	HYTIF--AAGDRERLHGH	[125]	163
	Legionella pneumophila pneumophila Philadelphia 1 [YP_096859]	[20]	HTTIF--SATEREPLHGH	[128]	164
	Thiorhodococcus sp. AK35 [W9V734]	[21]	HFTIF--SASERENLHGH	[127]	164
	Cystobacter fuscus DSM 2262 [S9R713]	[19]	HFTIF--SATHRENMHGH	[130]	165
	Enhygromyxa salina [A0A0C2D8S3]	[19]	HFTIY--DASHRERLHGH	[132]	167
	Rhodopirellula islandica [A0A0J1BA84]	[19]	HFITF--AGDICERIHGH	[129]	164
	Rubripirellula obstinata [WP_068265057]	[14]	HRLFE--HGGKCEHFHGH	[129]	159
	Thermococcus sp. 2319x1 [A0A0U3IC94]	[14]	HAVK---TNGELEEIHGH	[.86]	115
	hydrocarbon metagenome [A0A0W8G7C5]	[25]	HCLRH--YGGPCENLHGH	[100]	143
			* ***		

B

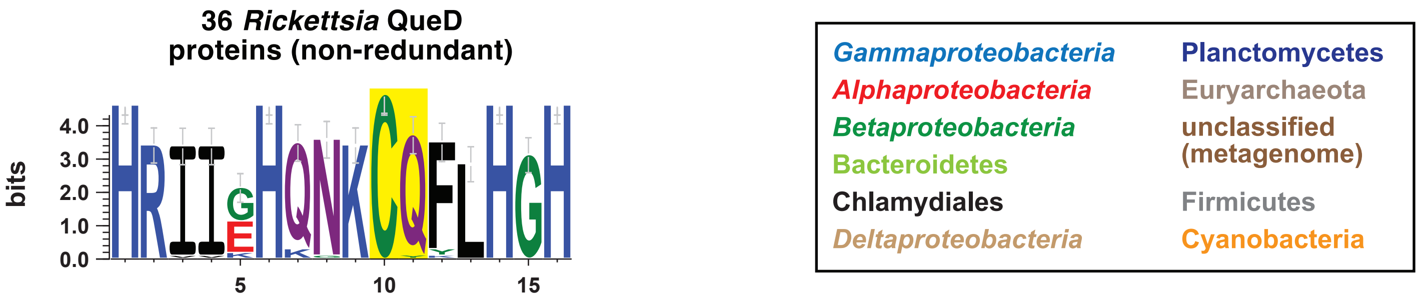

C

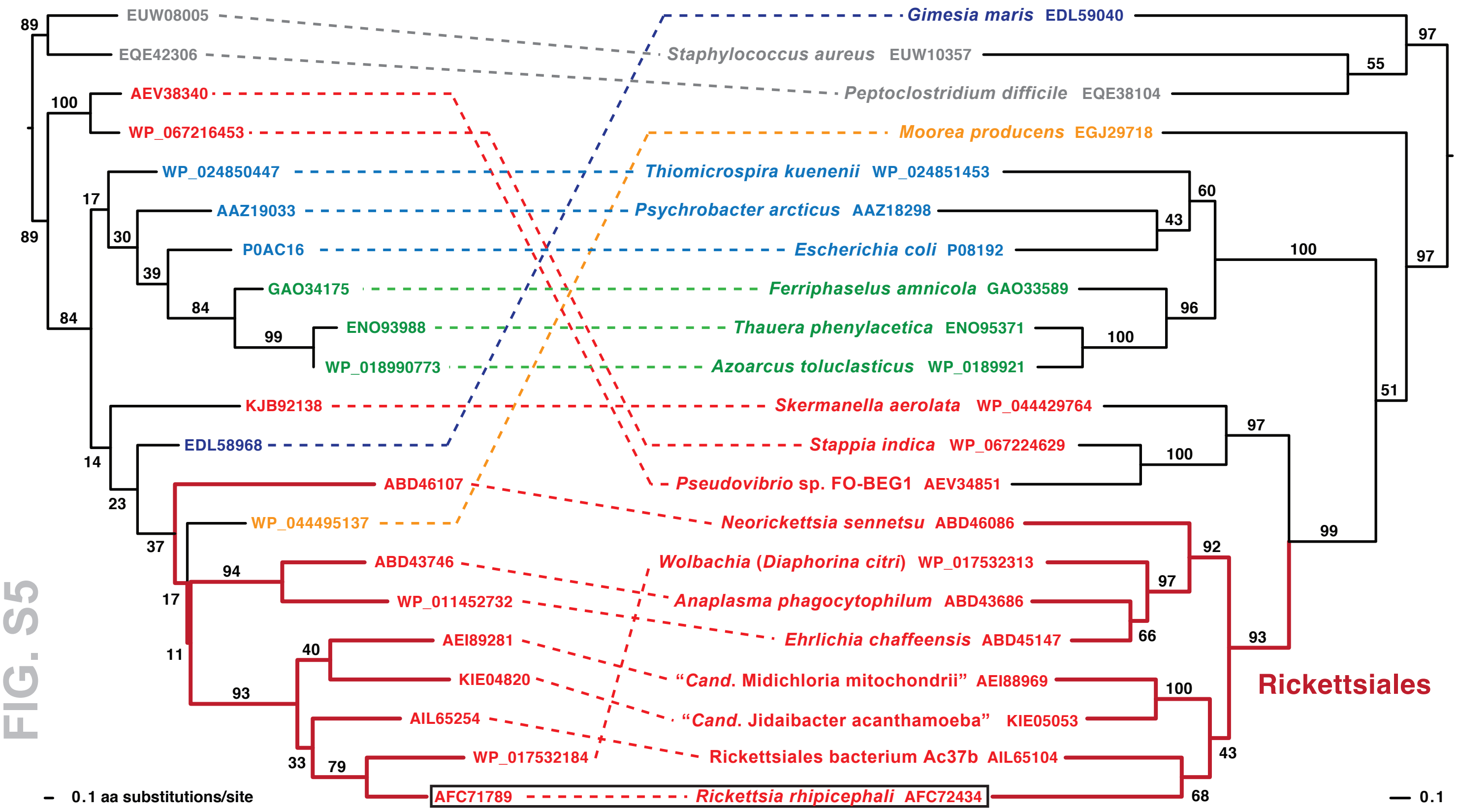

FIG. S5

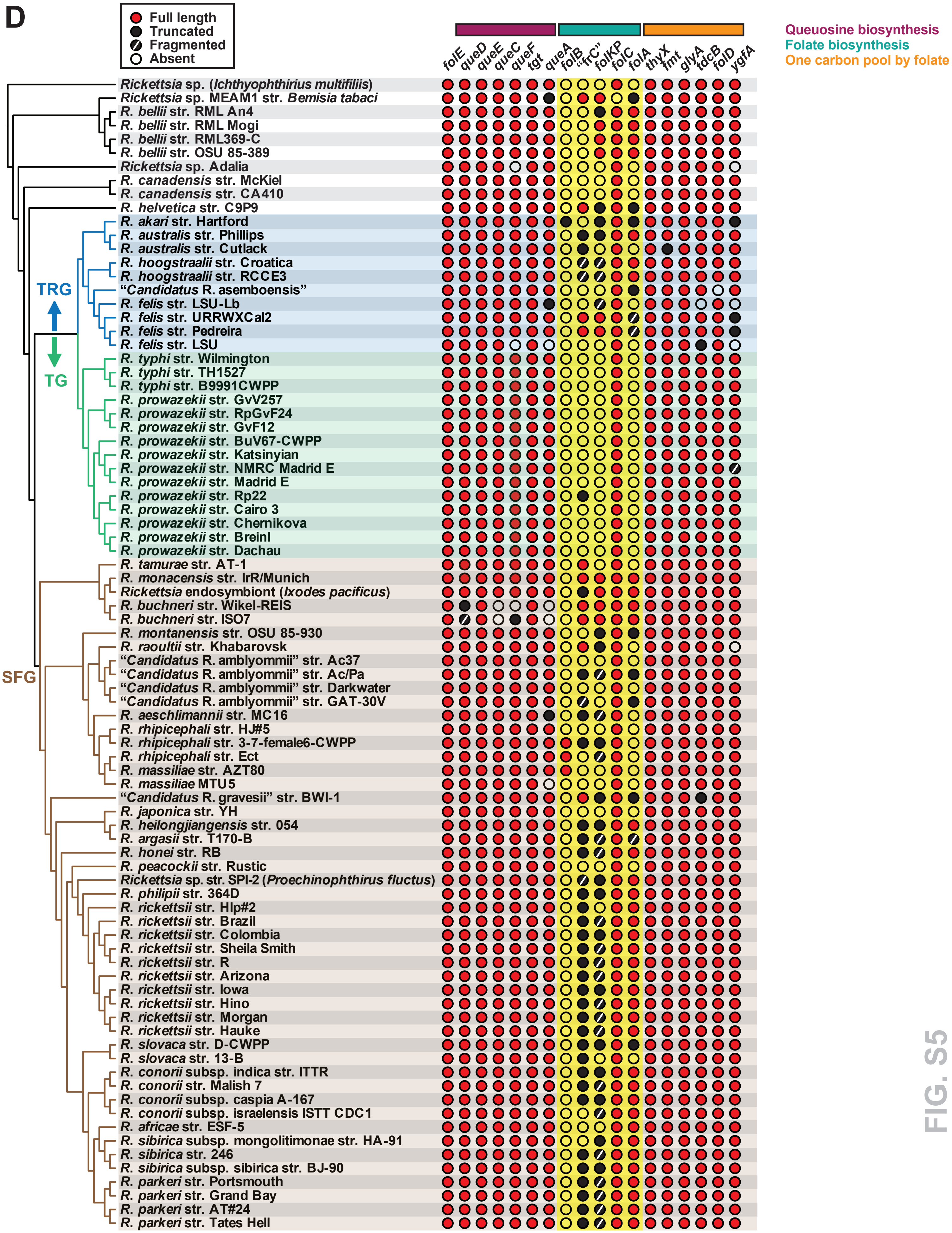
D
Full length
Truncated
Fragmented
Absent
Queuosine biosynthesis
Folate biosynthesis
One carbon pool by folate
folE
queD
queE
queC
queF
tgt
queA
folB
"frC"
folKP
folC
folA
thyX
fmt
glyA
tdcB
folD
ygfA
Rickettsia sp. (Ichthyophthirius multifiliis)
Rickettsia sp. MEAM1 str. Bemisia tabaci
R. bellii str. RML An4
R. bellii str. RML Mogi
R. bellii str. RML369-C
R. bellii str. OSU 85-389
Rickettsia sp. Adalia
R. canadensis str. McKiel
R. canadensis str. CA410
R. helvetica str. C9P9
R. akari str. Hartford
R. australis str. Phillips
R. australis str. Cutlack
R. hoogstraalii str. Croatica
R. hoogstraalii str. RCCE3
"Candidatus R. asemboensis"
R. felis str. LSU-Lb
R. felis str. URRWXCal2
R. felis str. Pedreira
R. felis str. LSU
R. typhi str. Wilmington
R. typhi str. TH1527
R. typhi str. B9991CWPP
R. prowazekii str. GvV257
R. prowazekii str. RpGvF24
R. prowazekii str. GvF12
R. prowazekii str. BuV67-CWPP
R. prowazekii str. Katsinyian
R. prowazekii str. NMRC Madrid E
R. prowazekii str. Madrid E
R. prowazekii str. Rp22
R. prowazekii str. Cairo 3
R. prowazekii str. Chernikova
R. prowazekii str. Breinl
R. prowazekii str. Dachau
R. tamurae str. AT-1
R. monacensis str. IrR/Munich
Rickettsia endosymbiont (Ixodes pacificus)
R. buchneri str. Wikel-REIS
R. buchneri str. ISO7
R. montanensis str. OSU 85-930
R. raoultii str. Khabarovsk
"Candidatus R. amblyommii" str. Ac37
"Candidatus R. amblyommii" str. Ac/Pa
"Candidatus R. amblyommii" str. Darkwater
"Candidatus R. amblyommii" str. GAT-30V
R. aeschlimannii str. MC16
R. rhipicephali str. HJ#5
R. rhipicephali str. 3-7-female6-CWPP
R. rhipicephali str. Ect
R. massiliae str. AZT80
R. massiliae MTU5
"Candidatus R. gravesii" str. BWI-1
R. japonica str. YH
R. heilongjiangensis str. 054
R. argasii str. T170-B
R. honei str. RB
R. peacockii str. Rustic
Rickettsia sp. str. SPI-2 (Proechinophthirus fluctus)
R. philipii str. 364D
R. rickettsii str. Hlp#2
R. rickettsii str. Brazil
R. rickettsii str. Colombia
R. rickettsii str. Sheila Smith
R. rickettsii str. R
R. rickettsii str. Arizona
R. rickettsii str. Iowa
R. rickettsii str. Hino
R. rickettsii str. Morgan
R. rickettsii str. Hauke
R. slovaca str. D-CWPP
R. slovaca str. 13-B
R. conorii subsp. indica str. ITTR
R. conorii str. Malish 7
R. conorii subsp. caspia A-167
R. conorii subsp. israelensis ISTT CDC1
R. africae str. ESF-5
R. sibirica subsp. mongolitimonae str. HA-91
R. sibirica str. 246
R. sibirica subsp. sibirica str. BJ-90
R. parkeri str. Portsmouth
R. parkeri str. Grand Bay
R. parkeri str. AT#24
R. parkeri str. Tates Hell
TRG
TG
SFG

FIG. S5
