# Supplementary material for: Wholly Rickettsia! Reconstructed Metabolic Profile of the Quintessential Bacterial Parasite of Eukaryotic Cells
Source: mBio. 2017 Sep 26;8(5):e00859-17. doi: 10.1128/mBio.00859-17 (PMC5615194; doi:10.1128/mBio.00859-17)
Supplement: FIG S6 [file mbo005173495sf6.pdf]

**FIG S6. *Rickettsia* queuosine biosynthesis contains a pathway hole for the reduction of epoxyqueuosine to queuosine.**

(A) Enzymes functioning in the pathway from 7,8-dihydroneopterin 3'-triphosphate to queuosine occur in varying frequencies across 3493 bacterial genomes (all prokaryotic genomes with an available queuosine biosynthesis pathway at KEGG). The star depicts the pathway hole (no QueG) in the *Rickettsia* queuosine pathway.

(B) Relative to the "*E. coli*-like" pathway, the "*Rickettsia*-like" pathway for queuosine biosynthesis is far less common, occurring in only 61 other bacterial genomes (excluding *Rickettsia* genomes). The pathway hole (no QueG) is noted with a red X.

(C) Taxonomic breakdown of bacterial species containing the "*Rickettsia*-like" queuosine biosynthesis pathway (61 total genomes). Genomes from intracellular species represent 51% of the total number of "*Rickettsia*-like" pathways.

## A

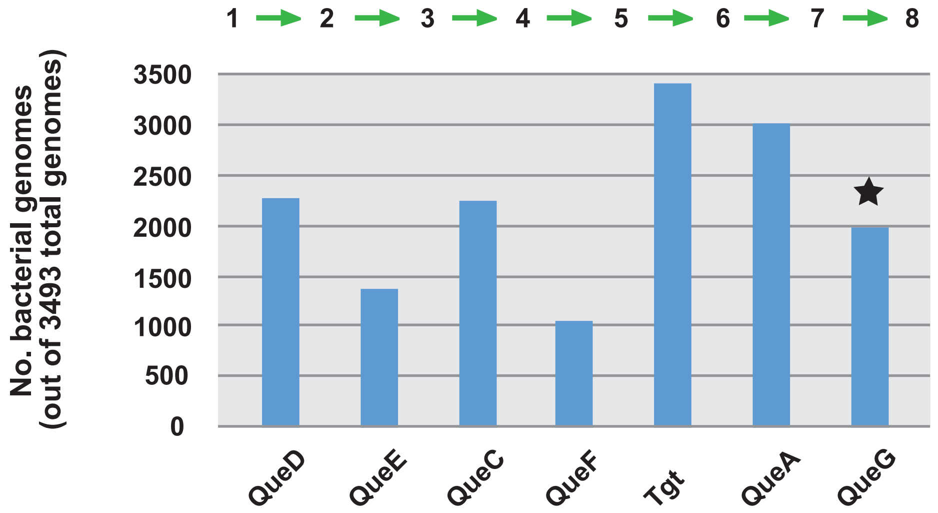


| | | | |
|---|---|---|---|
| 1 | 7,8-dihydroneopterin 3'-triphosphate | 5 | 7-aminomethyl-7-carbaguanine |
| 2 | 6-carboxy-5,6,7,8-tetra-hydropterin | 6 | 7-aminomethyl-7-deaza-guanosine[34] (in tRNA) |
| 3 | 7-carboxy-7-carbaguanine | 7 | epoxy-queuosine[34] (in tRNA) |
| 4 | 7-cyano-7-carbaguanine | 8 | queuosine[34] (in tRNA) |

## B

| Pathway | Composition | No. genomes |
|---|---|---|
| *E. coli*-like | 1 → 2 → 3 → 4 → 5 → 6 → 7 → 8 | 567 |
| *Rickettsia*-like | 1 → 2 → 3 → 4 → 5 → 6 → 7 ⇸ 8 | 61 |

FIG. S6

## C

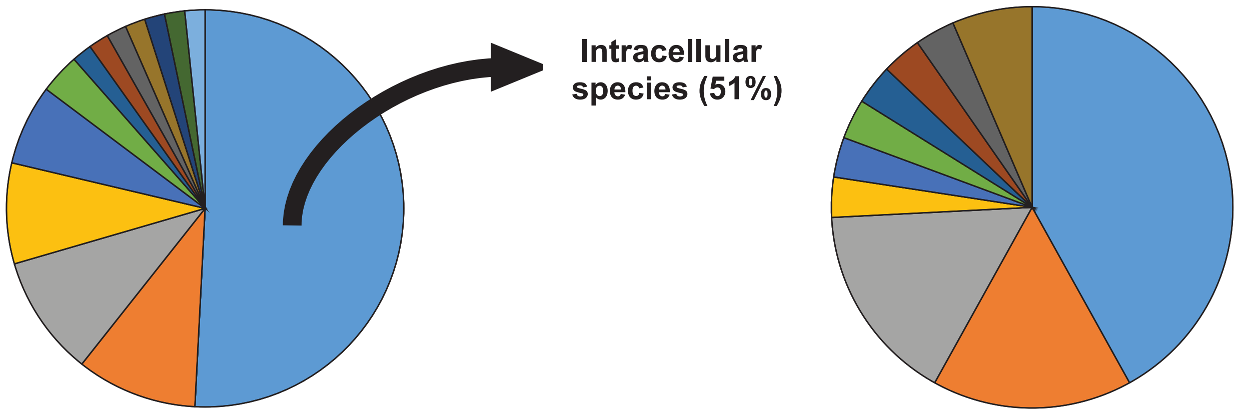


| | | | |
|---|---|---|---|
| *Erwinia* | *Desulfotalea* | *Legionella* | *Piscirickettsia* |
| *Xylella* | *Acinetobacter* | *Buchnera* | *Sodalis* |
| *Moraxella* | *Escherichia* | *Coxiella* | *Tachikawaea* |
| *Xanthomonas* | *Salmonella* | *Hamiltonella* | *Baumannia* |
| *Pantoea* | *Vibrio* | 2° endosymbiont of *Ctenarytaina eucalypti* | |
| *Odoribacter* | *Xenorhabdus* | 2° endosymbiont of *Heteropsylla cubana* | |
