# Supplementary material for: Wholly Rickettsia! Reconstructed Metabolic Profile of the Quintessential Bacterial Parasite of Eukaryotic Cells
Source: mBio. 2017 Sep 26;8(5):e00859-17. doi: 10.1128/mBio.00859-17 (PMC5615194; doi:10.1128/mBio.00859-17)
Supplement: FIG S7 [file mbo005173495sf7.pdf]

FIG S7. The evolutionary trajectory of six *Rickettsia* biosynthetic pathways that contain holes, or "missing enzymes".

Our *Rickettsia* metabolic reconstruction identified six holes occurring in pathways for the synthesis of CMP-3-deoxy-β-D-manno-octulosonate (CMP-Kdo), Diaminopimelate (DAP), CDP-diacylglycerol (CDP-DG), terpenoid backbones, ubiquinone (CoQ_8), and queuosine (see manuscript for further details). These pathways were analyzed in the genomes of Rickettsiales and Holosporales to determine the presence/absence of the missing enzymes. If present, phylogenies were estimated to determine if the genes encoding these enzymes were present in the Rickettsiales/Holosporales common ancestor.

(A) *E. coli* enzymes corresponding to the holes in the *Rickettsia* CMP-Kdo (KdsC), DAP (DapC), CDP-DG (PlsX, PlsY or PlsB), terpenoid backbones (IspA), CoQ_8 (UbiC, UbiI), and queuosine (QueG) pathways were used as queries in blastp searches against the Rickettsiales and Holosporales databases at NCBI. For significant matches, only top hits with coverage to the query greater than 85% were considered. The four obtained rickettsial homologs were then used as queries in blastp searches against the *Alphaproteobacteria* (excluding Rickettsiales and Holosporales) and the NR (excluding *Alphaproteobacteria*) databases at NCBI. Again, for significant matches, only top hits with coverage to the query greater than 85% were considered.

(B) Phylogenomics analysis of CMP-Kdo, DAP, CDP-DG, terpenoid backbones, CoQ_8, and queuosine pathways across selected Holosporales and Rickettsiales genomes. The presence/absence of enzymes corresponding to the holes in the *Rickettsia* pathways are shown at right. Genome-based phylogeny was estimated on 105 orthologous groups (OGs) of proteins (single copy CDS in over 95% of genomes). We sampled 53 Rickettsiales/Holosporales and three outgroup *Alphaproteobacteria* genomes: IDs for sequences retrieved from PATRIC (P) and NCBI (N) are shown for each taxon. Several genomes were specifically annotated for this project: *Caedibacter varicaedens* (NCBI, NZ_BBVC00000000.1); "*Candidatus* Arcanobacter lacustris" str. SCGC (NCBI, JYHA00000000.1); "*Candidatus* Paracaedibacter symbiosus" (NCBI,

NZ_JQAK00000000.1); "*Candidatus* Xenolissoclinum pacificiensis" str. L6 (NCBI, AXCJ00000000.1); *Rickettsia tamurae* str. AT-1 (NCBI, CCMG01000000.1); "*Candidatus* Hepatobacter penaei" str. NHPB (NCBI, JQAJ00000000.1); *Rickettsia* endosymbiont of *Adalia bipunctata* (unpublished); *Rickettsia* endosymbiont of *Ichthyophthirius multifiliis* (unpublished); *Rickettsia* sp. MEAM1 str. *Bemisia tabaci* (unpublished). Data for Rickettsiales endosymbiont of *Trichoplax adhaerens* is described elsewhere (1). OGs were generated using FastOrtho, a modified version of OrthoMCL (2). Multiple sequence alignment of each OG was performed using MUSCLE (default parameters) (3), with regions of poor alignment (length heterogeneous regions) masked using Gblocks (4). All modified alignments were concatenated into a single dataset for phylogeny estimation. Using PhyloBayes MPI (5), we analyzed the dataset with the CAT model of substitution, a nonparametric method for modeling site-specific features of sequence evolution (6, 7). Given the strong base compositional bias of rickettsial genomes (~30 %GC), the ability of the CAT model to accommodate saturation due to convergences and reversions (8) is of substantial importance for estimating rickettsial phylogeny, as demonstrated by us and others (1, 9–11). Two independent Markov chains were run in parallel using PhyloBayes MPI v.1.2e under the CAT-GTR model, with the bipartition frequencies analyzed at various time points using the bpcomp program. For tree-building, appropriate burn-in values were determined by plotting the log likelihoods for each chain over sampled generations (time). Analyses were considered complete when the maximum difference in bipartition frequencies between the two chains was less than 0.1. Ultimately, a burn-in value of 1000, with sampling every 2 trees, was used to build a consensus tree.

(C-F) Phylogeny estimations of DapC (C), PlsX (D), PlsY (E), and IspA (F) proteins. Datasets for each protein were constructed as follows: the rickettsial protein (panel A) was used in blastp queries against several taxon-specific databases: 1) "Rickettsiales", 2) "Holosporales", 3) "*Alphaproteobacteria* (minus Rickettsiales and Holosporales)", 4) "*Proteobacteria* (minus *Alphaproteobacteria*)", 5) "Bacteria (minus *Proteobacteria*)", and 6) "minus Bacteria". The top 5-

10 (query-dependent) subjects from each search resulting in significant (> 40 bits) alignments were all compiled and aligned using MUSCLE v3.8.31 (default parameters). Protein phylogenies were estimated under maximum likelihood with RAxML v8.2.4 (12), using a gamma model of rate heterogeneity and estimation of the proportion of invariant sites. Both the Lee and Gascuel (LG) and Blocks of Amino Acid Substitution Matrix (BLOSUM62) models of amino acid substitution were used, and branch support was assessed with 1,000 pseudo-replications.

REFERENCES

1. **Driscoll T**, **Gillespie JJ**, **Nordberg EK**, **Azad AF**, **Sobral BW**. 2013. Bacterial DNA sifted from the Trichoplax adhaerens (Animalia: Placozoa) genome project reveals a putative rickettsial endosymbiont. Genome Biol Evol **5**:621–45.
2. **Li L**, **Stoeckert CJ**, **Roos DS**. 2003. OrthoMCL: identification of ortholog groups for eukaryotic genomes. Genome Res **13**:2178–89.
3. **Edgar RC**. 2004. MUSCLE: Multiple sequence alignment with high accuracy and high throughput. Nucleic Acids Res **32**:1792–1797.
4. **Talavera G**, **Castresana J**. 2007. Improvement of phylogenies after removing divergent and ambiguously aligned blocks from protein sequence alignments. Syst Biol **56**:564–77.
5. **Lartillot N**, **Rodrigue N**, **Stubbs D**, **Richer J**. 2013. PhyloBayes MPI: phylogenetic reconstruction with infinite mixtures of profiles in a parallel environment. Syst Biol **62**:611–5.
6. **Lartillot N**, **Philippe H**. 2004. A Bayesian Mixture Model for Across-Site Heterogeneities in the Amino-Acid Replacement Process. Mol Biol Evol **21**:1095–1109.
7. **Lartillot N**, **Philippe H**. 2006. Computing Bayes factors using thermodynamic integration. Syst Biol **55**:195–207.
8. **Lartillot N**, **Brinkmann H**, **Philippe H**. 2007. Suppression of long-branch attraction artefacts in the animal phylogeny using a site-heterogeneous model. BMC Evol Biol **7**

Suppl 1:S4.

9. **Rodríguez-Ezpeleta N**, **Embley TM**. 2012. The SAR11 group of alpha-proteobacteria is not related to the origin of mitochondria. PLoS One **7**:e30520.

10. **Viklund J**, **Ettema TJG**, **Andersson SGE**. 2012. Independent genome reduction and phylogenetic reclassification of the oceanic SAR11 clade. Mol Biol Evol **29**:599–615.

11. **Gillespie JJ**, **Driscoll TP**, **Verhoeve VI**, **Utsuki T**, **Husseneder C**, **Chouljenko VN**, **Azad AF**, **Macaluso KR**. 2014. Genomic diversification in strains of Rickettsia felis isolated from different arthropods. Genome Biol Evol.

12. **Stamatakis A**. 2014. RAxML version 8: A tool for phylogenetic analysis and post-analysis of large phylogenies. Bioinformatics **30**:1312–1313.

A

Hole	Reference protein	Top Rick (% ID)		Top *Alpha* E value; % ID		Top non-*Alpha* E value; % ID	
KdsC	P0ABZ4: *E. coli* str. K12	---		---		---	
DapC	P18335: *E. coli* str. K12	WP_070064655:	*Wolbachia pipientis* (42%)	4e-130;	50%	1e-122;	48%
PlsX	P27247: *E. coli* str. K12	WP_011452016:	*Neorickettsia sennetsu* (45%)	9e-94;	47%	4e-77;	45%
PlsY	P60782: *E. coli* str. K12	KKB96285:	"*Cand.* Arcanobacter lacustris" (40%)	5e-50;	49%	2e-36;	49%
PlsB	P0A7A7: *E. coli* str. K12	---		---		---	
IspA	P22939: *E. coli* str. K12	WP_039459403:	"*Cand.* Jidaibacter acanthamoeba" (44%)	7e-109;	59%	2e-72;	55%
UbiC	P26602: *E. coli* str. K12	---		---		---	
UbiI	P25535: *E. coli* str. K12	---		---		---	
QueG	P39288: *E. coli* str. K12	---		---		---	

B

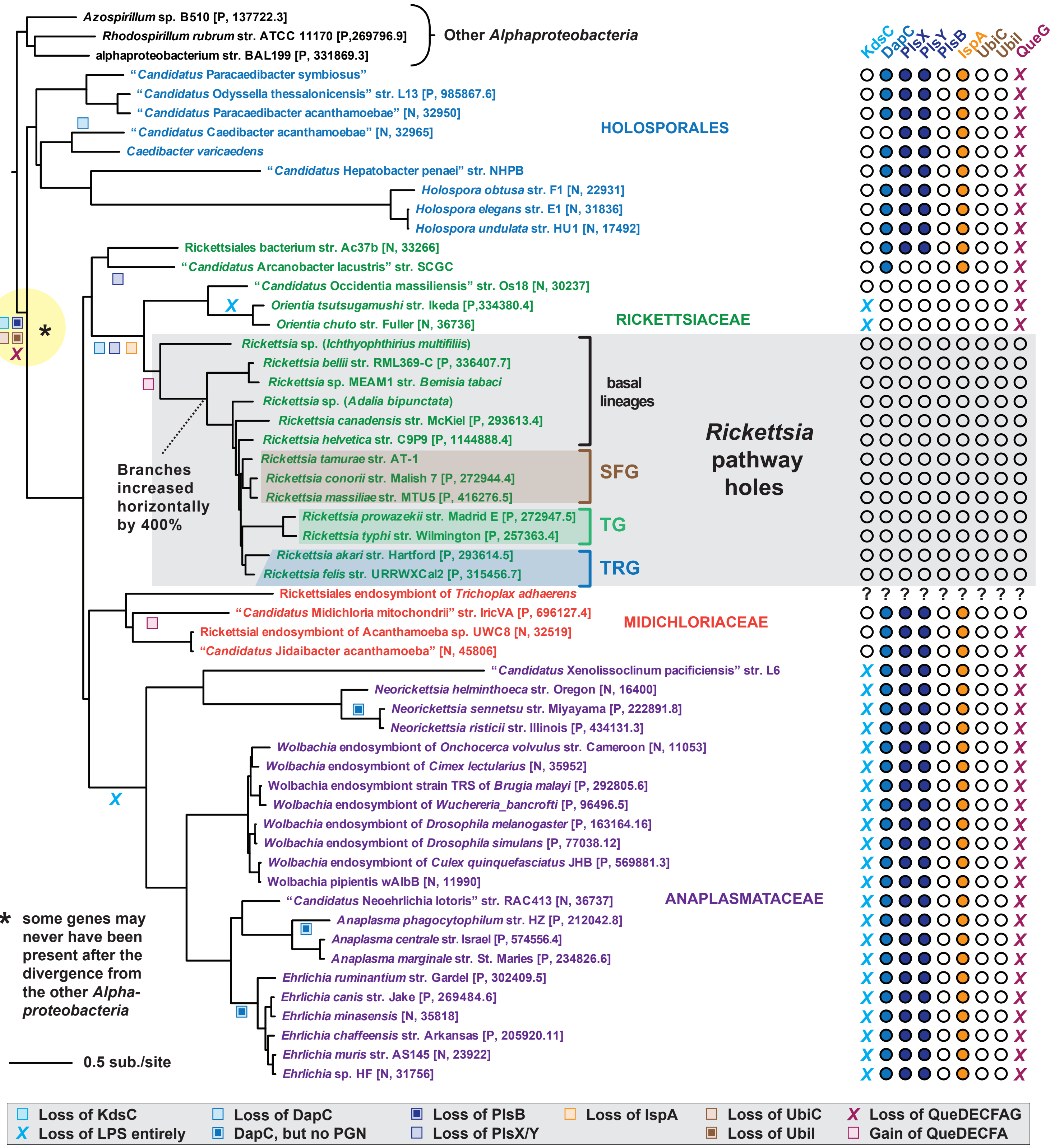

FIG. S7

C

DapC: *N*-succinyldiaminopimelate aminotransferase

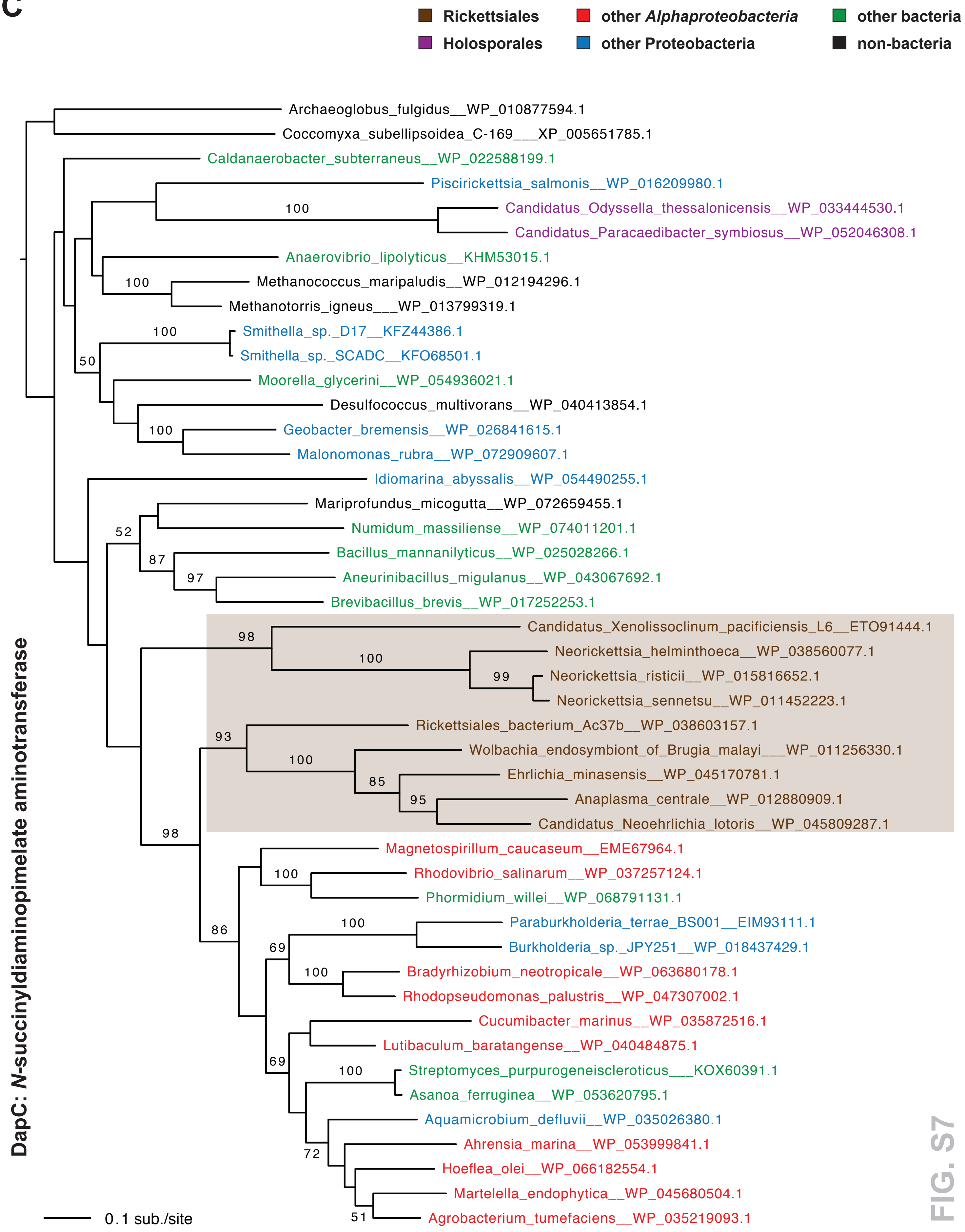

FIG. S7

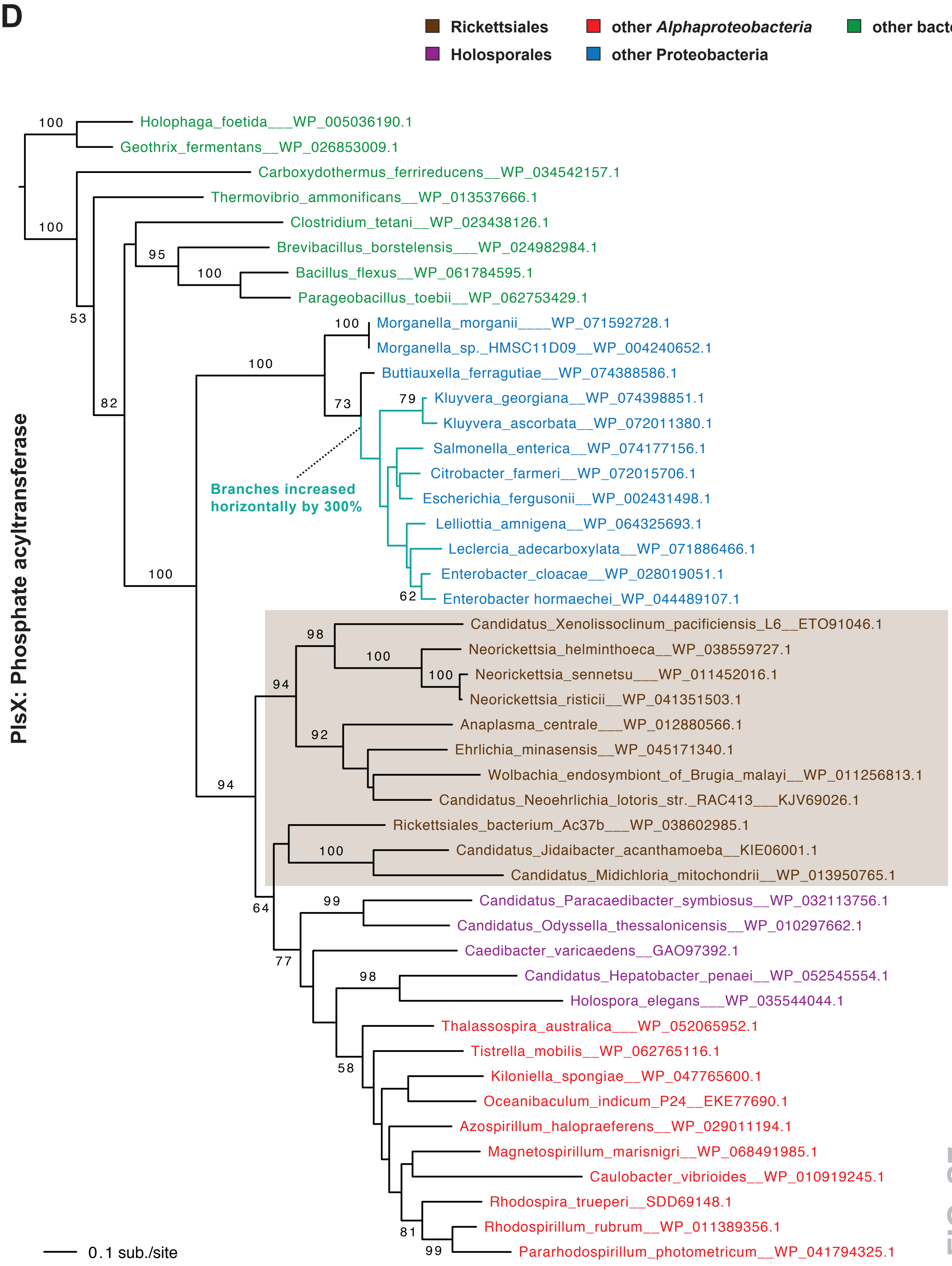
D
Rickettsiales
Holosporales
other Alphaproteobacteria
other Proteobacteria
other bacteria
PlsX: Phosphate acyltransferase
Holophaga_foetida___WP_005036190.1
Geothrix_fermentans__WP_026853009.1
Carboxydothermus_ferrireducens__WP_034542157.1
Thermovibrio_ammonificans__WP_013537666.1
Clostridium_tetani__WP_023438126.1
Brevibacillus_borstelensis___WP_024982984.1
Bacillus_flexus__WP_061784595.1
Parageobacillus_toebii__WP_062753429.1
Morganella_morganii____WP_071592728.1
Morganella_sp._HMSC11D09__WP_004240652.1
Buttiauxella_ferragutiae__WP_074388586.1
Kluyvera_georgiana__WP_074398851.1
Kluyvera_ascorbata__WP_072011380.1
Salmonella_enterica__WP_074177156.1
Citrobacter_farmeri__WP_072015706.1
Escherichia_fergusonii__WP_002431498.1
Lelliottia_amnigena__WP_064325693.1
Leclercia_adecarboxylata__WP_071886466.1
Enterobacter_cloacae__WP_028019051.1
Enterobacter hormaechei_WP_044489107.1
Branches increased horizontally by 300%
Candidatus_Xenolissoclinum_pacificiensis_L6__ETO91046.1
Neorickettsia_helminthoeca__WP_038559727.1
Neorickettsia_sennetsu___WP_011452016.1
Neorickettsia_risticii__WP_041351503.1
Anaplasma_centrale___WP_012880566.1
Ehrlichia_minasensis__WP_045171340.1
Wolbachia_endosymbiont_of_Brugia_malayi__WP_011256813.1
Candidatus_Neoehrlichia_lotoris_str._RAC413___KJV69026.1
Rickettsiales_bacterium_Ac37b___WP_038602985.1
Candidatus_Jidaibacter_acanthamoeba__KIE06001.1
Candidatus_Midichloria_mitochondrii__WP_013950765.1
Candidatus_Paracaedibacter_symbiosus__WP_032113756.1
Candidatus_Odyssella_thessalonicensis__WP_010297662.1
Caedibacter_varicaedens__GAO97392.1
Candidatus_Hepatobacter_penaei__WP_052545554.1
Holospora_elegans___WP_035544044.1
Thalassospira_australica___WP_052065952.1
Tistrella_mobilis__WP_062765116.1
Kiloniella_spongiae__WP_047765600.1
Oceanibaculum_indicum_P24__EKE77690.1
Azospirillum_halopraeferens__WP_029011194.1
Magnetospirillum_marisnigri__WP_068491985.1
Caulobacter_vibrioides__WP_010919245.1
Rhodospira_trueperi__SDD69148.1
Rhodospirillum_rubrum__WP_011389356.1
Pararhodospirillum_photometricum__WP_041794325.1
100
100
95
100
53
82
100
100
73
79
62
100
98
100
100
94
92
94
100
64
99
77
98
58
81
99
0.1 sub./site

FIG. S7

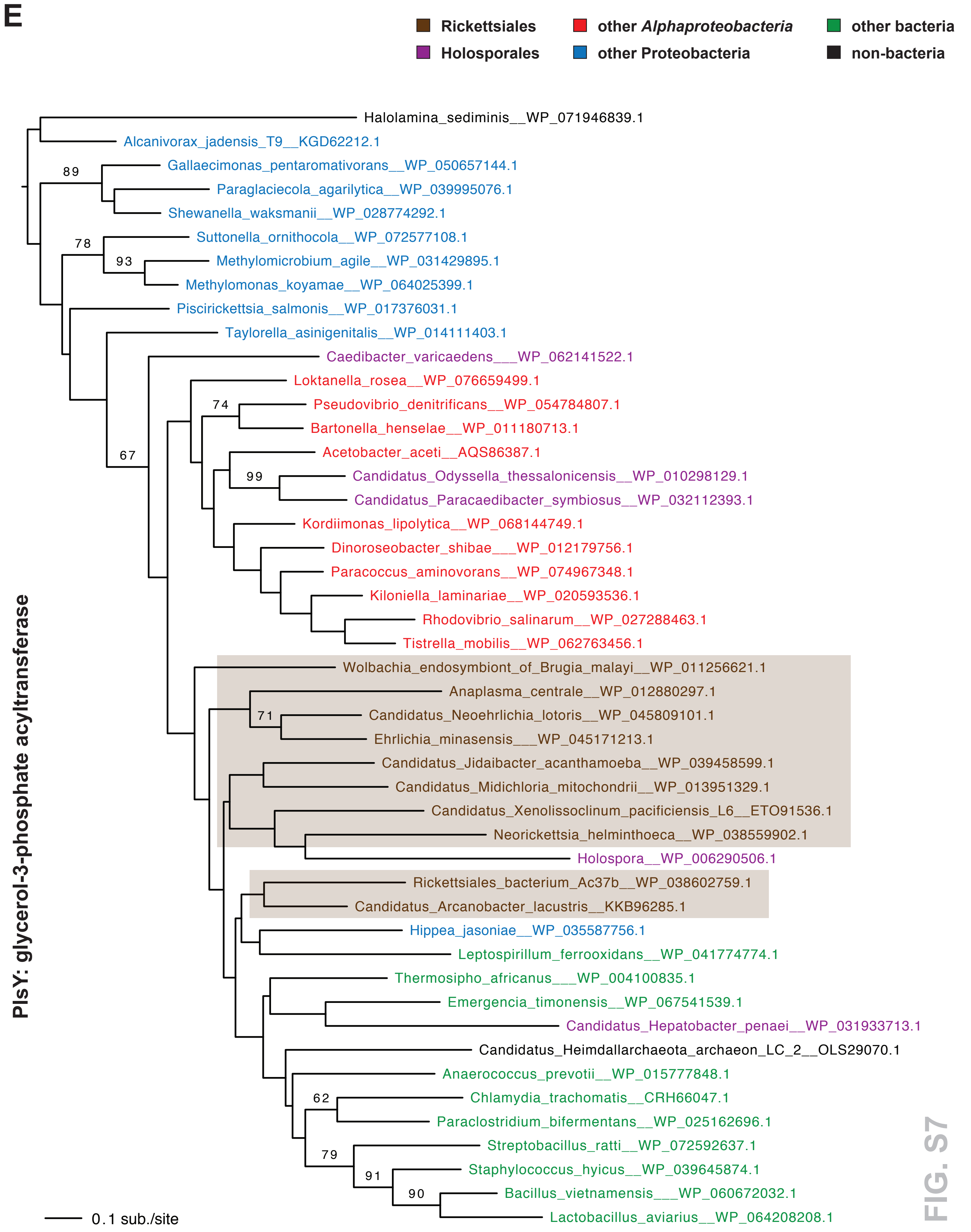
E
Rickettsiales
Holosporales
other Alphaproteobacteria
other Proteobacteria
other bacteria
non-bacteria
PlsY: glycerol-3-phosphate acyltransferase
Halolamina_sediminis__WP_071946839.1
Alcanivorax_jadensis_T9__KGD62212.1
Gallaecimonas_pentaromativorans__WP_050657144.1
Paraglaciecola_agarilytica__WP_039995076.1
Shewanella_waksmanii__WP_028774292.1
Suttonella_ornithocola__WP_072577108.1
Methylomicrobium_agile__WP_031429895.1
Methylomonas_koyamae__WP_064025399.1
Piscirickettsia_salmonis__WP_017376031.1
Taylorella_asinigenitalis__WP_014111403.1
Caedibacter_varicaedens___WP_062141522.1
Loktanella_rosea__WP_076659499.1
Pseudovibrio_denitrificans__WP_054784807.1
Bartonella_henselae__WP_011180713.1
Acetobacter_aceti__AQS86387.1
Candidatus_Odyssella_thessalonicensis__WP_010298129.1
Candidatus_Paracaedibacter_symbiosus__WP_032112393.1
Kordiimonas_lipolytica__WP_068144749.1
Dinoroseobacter_shibae___WP_012179756.1
Paracoccus_aminovorans__WP_074967348.1
Kiloniella_laminariae__WP_020593536.1
Rhodovibrio_salinarum__WP_027288463.1
Tistrella_mobilis__WP_062763456.1
Wolbachia_endosymbiont_of_Brugia_malayi__WP_011256621.1
Anaplasma_centrale__WP_012880297.1
Candidatus_Neoehrlichia_lotoris__WP_045809101.1
Ehrlichia_minasensis___WP_045171213.1
Candidatus_Jidaibacter_acanthamoeba__WP_039458599.1
Candidatus_Midichloria_mitochondrii__WP_013951329.1
Candidatus_Xenolissoclinum_pacificiensis_L6__ETO91536.1
Neorickettsia_helminthoeca__WP_038559902.1
Holospora__WP_006290506.1
Rickettsiales_bacterium_Ac37b__WP_038602759.1
Candidatus_Arcanobacter_lacustris__KKB96285.1
Hippea_jasoniae__WP_035587756.1
Leptospirillum_ferrooxidans__WP_041774774.1
Thermosipho_africanus___WP_004100835.1
Emergencia_timonensis__WP_067541539.1
Candidatus_Hepatobacter_penaei__WP_031933713.1
Candidatus_Heimdallarchaeota_archaeon_LC_2__OLS29070.1
Anaerococcus_prevotii__WP_015777848.1
Chlamydia_trachomatis__CRH66047.1
Paraclostridium_bifermentans__WP_025162696.1
Streptobacillus_ratti__WP_072592637.1
Staphylococcus_hyicus__WP_039645874.1
Bacillus_vietnamensis___WP_060672032.1
Lactobacillus_aviarius__WP_064208208.1
89
78
93
74
67
99
71
62
79
91
90
0.1 sub./site

FIG. S7

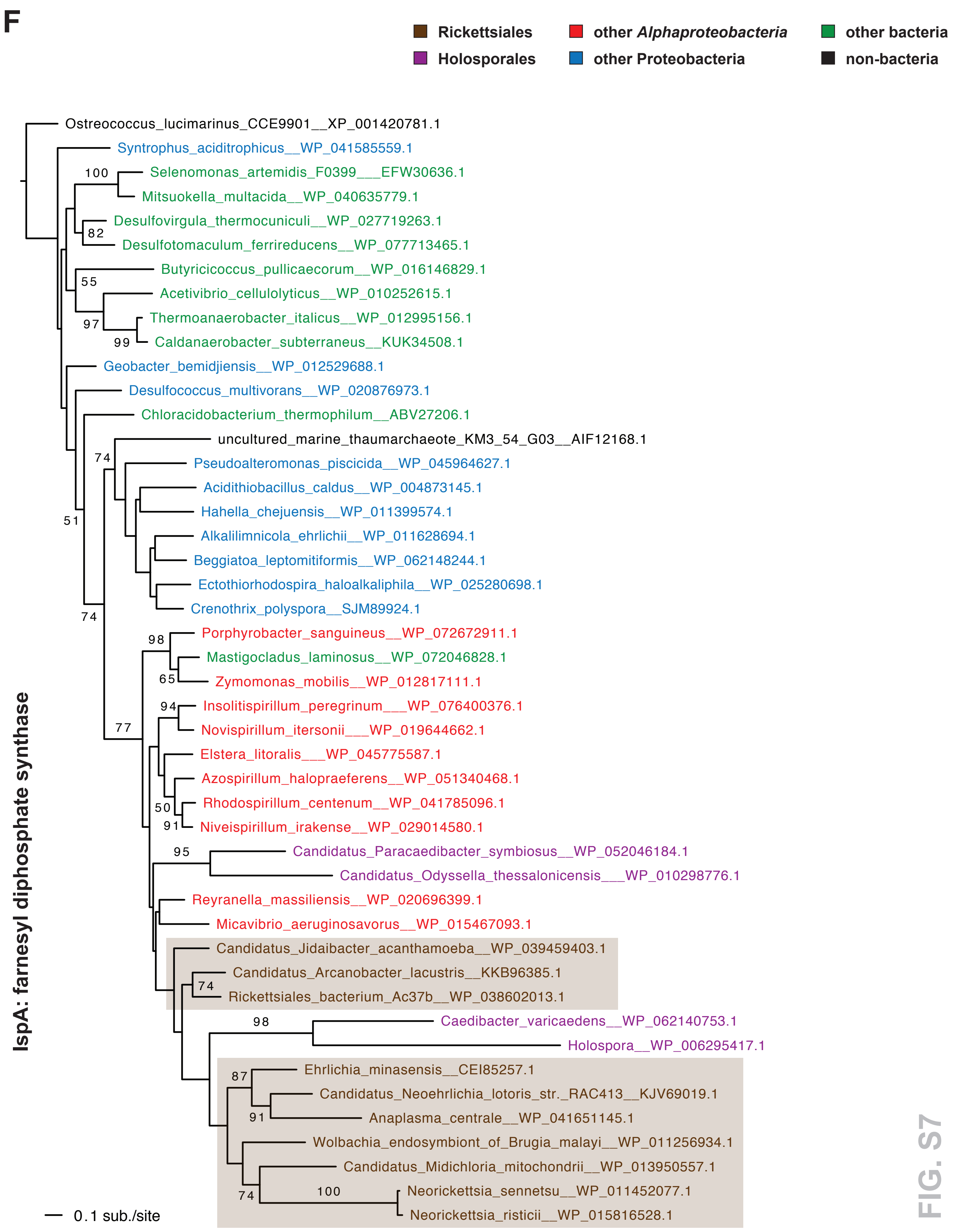
F
Rickettsiales
Holosporales
other Alphaproteobacteria
other Proteobacteria
other bacteria
non-bacteria
IspA: farnesyl diphosphate synthase
Ostreococcus_lucimarinus_CCE9901__XP_001420781.1
Syntrophus_aciditrophicus__WP_041585559.1
Selenomonas_artemidis_F0399___EFW30636.1
Mitsuokella_multacida__WP_040635779.1
Desulfovirgula_thermocuniculi__WP_027719263.1
Desulfotomaculum_ferrireducens__WP_077713465.1
Butyricicoccus_pullicaecorum__WP_016146829.1
Acetivibrio_cellulolyticus__WP_010252615.1
Thermoanaerobacter_italicus__WP_012995156.1
Caldanaerobacter_subterraneus__KUK34508.1
Geobacter_bemidjiensis__WP_012529688.1
Desulfococcus_multivorans__WP_020876973.1
Chloracidobacterium_thermophilum__ABV27206.1
uncultured_marine_thaumarchaeote_KM3_54_G03__AIF12168.1
Pseudoalteromonas_piscicida__WP_045964627.1
Acidithiobacillus_caldus__WP_004873145.1
Hahella_chejuensis__WP_011399574.1
Alkalilimnicola_ehrlichii__WP_011628694.1
Beggiatoa_leptomitiformis__WP_062148244.1
Ectothiorhodospira_haloalkaliphila__WP_025280698.1
Crenothrix_polyspora__SJM89924.1
Porphyrobacter_sanguineus__WP_072672911.1
Mastigocladus_laminosus__WP_072046828.1
Zymomonas_mobilis__WP_012817111.1
Insolitispirillum_peregrinum___WP_076400376.1
Novispirillum_itersonii___WP_019644662.1
Elstera_litoralis___WP_045775587.1
Azospirillum_halopraeferens__WP_051340468.1
Rhodospirillum_centenum__WP_041785096.1
Niveispirillum_irakense__WP_029014580.1
Candidatus_Paracaedibacter_symbiosus__WP_052046184.1
Candidatus_Odyssella_thessalonicensis___WP_010298776.1
Reyranella_massiliensis__WP_020696399.1
Micavibrio_aeruginosavorus__WP_015467093.1
Candidatus_Jidaibacter_acanthamoeba__WP_039459403.1
Candidatus_Arcanobacter_lacustris__KKB96385.1
Rickettsiales_bacterium_Ac37b__WP_038602013.1
Caedibacter_varicaedens__WP_062140753.1
Holospora__WP_006295417.1
Ehrlichia_minasensis__CEI85257.1
Candidatus_Neoehrlichia_lotoris_str._RAC413__KJV69019.1
Anaplasma_centrale__WP_041651145.1
Wolbachia_endosymbiont_of_Brugia_malayi__WP_011256934.1
Candidatus_Midichloria_mitochondrii__WP_013950557.1
Neorickettsia_sennetsu__WP_011452077.1
Neorickettsia_risticii__WP_015816528.1
100
82
55
97
99
74
51
74
98
65
94
77
50
91
95
74
98
87
91
74
100
0.1 sub./site

FIG. S7
