# Supplementary material for: Wholly Rickettsia! Reconstructed Metabolic Profile of the Quintessential Bacterial Parasite of Eukaryotic Cells
Source: mBio. 2017 Sep 26;8(5):e00859-17. doi: 10.1128/mBio.00859-17 (PMC5615194; doi:10.1128/mBio.00859-17)
Supplement: FIG S8 [file mbo005173495sf8.pdf]

FIG S8. Phylogeny estimation of isopentenyl-diphosphate delta-isomerase (*idi*) and queuosine biosynthesis (*queD,E,C,F,A,H*) genes.

(A-H) Phylogeny estimations of Idi (A), Tgt (B), QueD (C), QueE (D), QueC (E), QueF (F), QueA (G), and QueH (H) proteins. Datasets for each protein were constructed as follows: the rickettsial protein was used in blastp queries against several taxon-specific databases: 1) “Rickettsiales”, 2) “Holosporales”, 3) “*Alphaproteobacteria* (minus Rickettsiales and Holosporales)”, 4) “*Proteobacteria* (minus *Alphaproteobacteria*)”, 5) “Bacteria (minus *Proteobacteria*)”, and 6) “minus Bacteria”. The top 5-10 (query-dependent) subjects from each search resulting in significant (> 40 bits) alignments were all compiled and aligned using MUSCLE v3.8.31 (default parameters) (1). Protein phylogenies were estimated under maximum likelihood with RAxML v8.2.4 (2) using a gamma model of rate heterogeneity and estimation of the proportion of invariant sites. Both the Lee and Gascuel (LG) and Blocks of Amino Acid Substitution Matrix (BLOSUM62) models of amino acid substitution were used, and branch support was assessed with 1,000 pseudo-replications.

REFERENCES

1. **Edgar RC**. 2004. MUSCLE: Multiple sequence alignment with high accuracy and high throughput. Nucleic Acids Res **32**:1792–1797.
2. **Stamatakis A**. 2014. RAxML version 8: A tool for phylogenetic analysis and post-analysis of large phylogenies. Bioinformatics **30**:1312–1313.

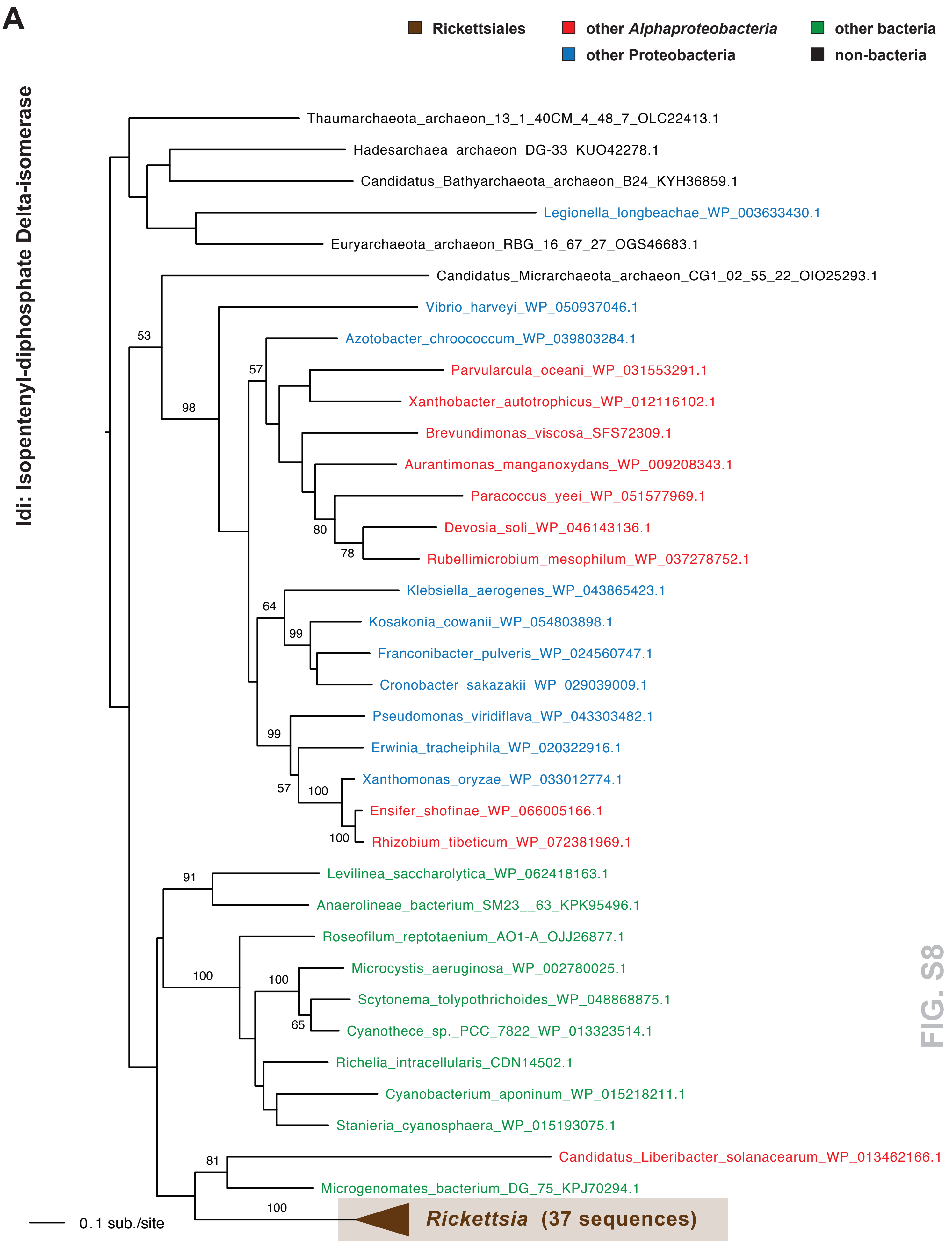
A
Rickettsiales
other Alphaproteobacteria
other bacteria
other Proteobacteria
non-bacteria
Idi: Isopentenyl-diphosphate Delta-isomerase
Thaumarchaeota_archaeon_13_1_40CM_4_48_7_OLC22413.1
Hadesarchaea_archaeon_DG-33_KUO42278.1
Candidatus_Bathyarchaeota_archaeon_B24_KYH36859.1
Legionella_longbeachae_WP_003633430.1
Euryarchaeota_archaeon_RBG_16_67_27_OGS46683.1
Candidatus_Micrarchaeota_archaeon_CG1_02_55_22_OIO25293.1
Vibrio_harveyi_WP_050937046.1
Azotobacter_chroococcum_WP_039803284.1
Parvularcula_oceani_WP_031553291.1
Xanthobacter_autotrophicus_WP_012116102.1
Brevundimonas_viscosa_SFS72309.1
Aurantimonas_manganoxydans_WP_009208343.1
Paracoccus_yeei_WP_051577969.1
Devosia_soli_WP_046143136.1
Rubellimicrobium_mesophilum_WP_037278752.1
Klebsiella_aerogenes_WP_043865423.1
Kosakonia_cowanii_WP_054803898.1
Franconibacter_pulveris_WP_024560747.1
Cronobacter_sakazakii_WP_029039009.1
Pseudomonas_viridiflava_WP_043303482.1
Erwinia_tracheiphila_WP_020322916.1
Xanthomonas_oryzae_WP_033012774.1
Ensifer_shofinae_WP_066005166.1
Rhizobium_tibeticum_WP_072381969.1
Levilinea_saccharolytica_WP_062418163.1
Anaerolineae_bacterium_SM23__63_KPK95496.1
Roseofilum_reptotaenium_AO1-A_OJJ26877.1
Microcystis_aeruginosa_WP_002780025.1
Scytonema_tolypothrichoides_WP_048868875.1
Cyanothece_sp._PCC_7822_WP_013323514.1
Richelia_intracellularis_CDN14502.1
Cyanobacterium_aponinum_WP_015218211.1
Stanieria_cyanosphaera_WP_015193075.1
Candidatus_Liberibacter_solanacearum_WP_013462166.1
Microgenomates_bacterium_DG_75_KPJ70294.1
Rickettsia (37 sequences)
53
98
57
80
78
64
99
99
57
100
100
91
100
100
65
81
100
0.1 sub./site

FIG. S8

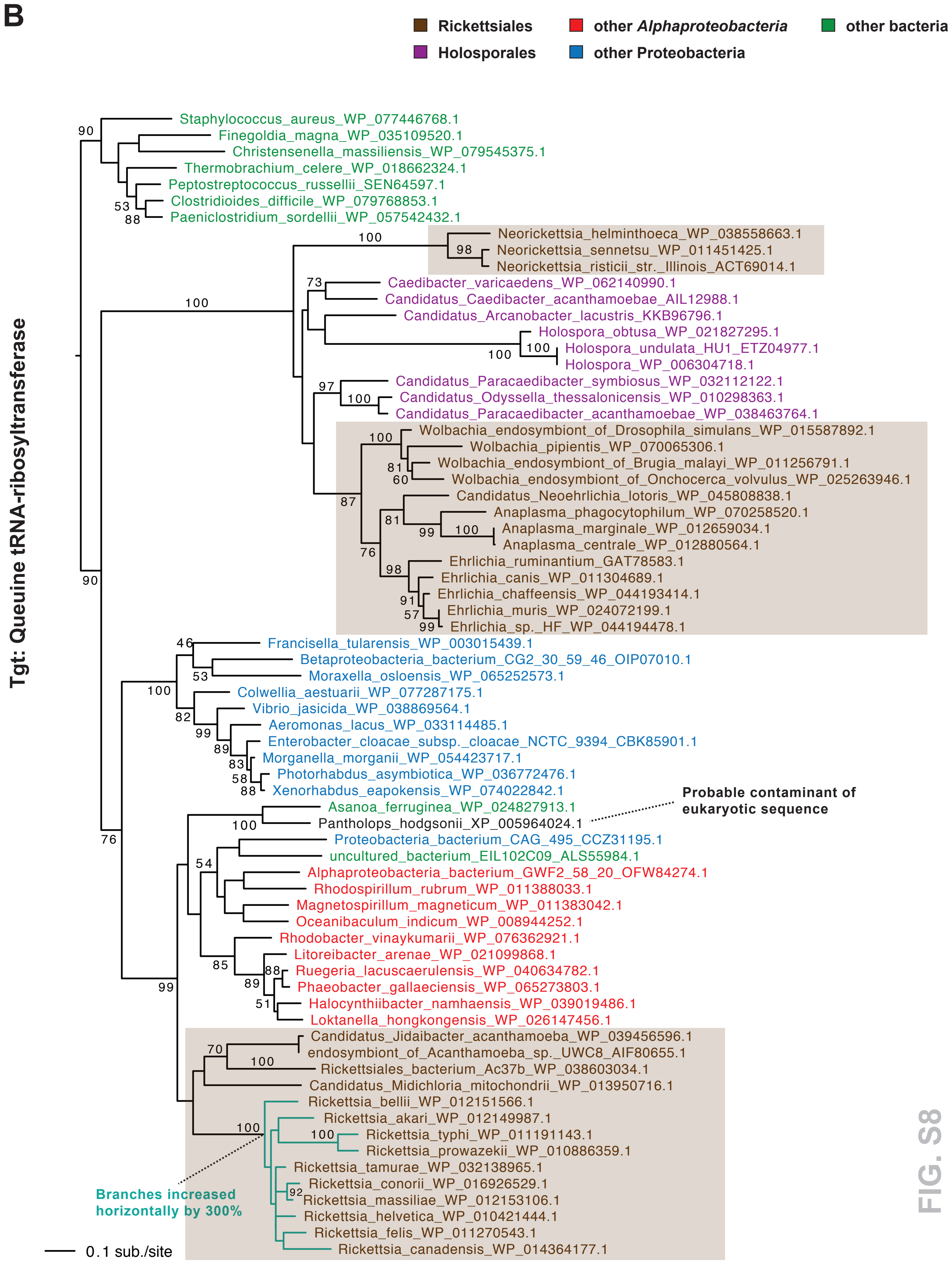
B
Rickettsiales
Holosporales
other Alphaproteobacteria
other Proteobacteria
other bacteria
Tgt: Queuine tRNA-ribosyltransferase
Staphylococcus_aureus_WP_077446768.1
Finegoldia_magna_WP_035109520.1
Christensenella_massiliensis_WP_079545375.1
Thermobrachium_celere_WP_018662324.1
Peptostreptococcus_russellii_SEN64597.1
Clostridioides_difficile_WP_079768853.1
Paeniclostridium_sordellii_WP_057542432.1
Neorickettsia_helminthoeca_WP_038558663.1
Neorickettsia_sennetsu_WP_011451425.1
Neorickettsia_risticii_str._Illinois_ACT69014.1
Caedibacter_varicaedens_WP_062140990.1
Candidatus_Caedibacter_acanthamoebae_AIL12988.1
Candidatus_Arcanobacter_lacustris_KKB96796.1
Holospora_obtusa_WP_021827295.1
Holospora_undulata_HU1_ETZ04977.1
Holospora_WP_006304718.1
Candidatus_Paracaedibacter_symbiosus_WP_032112122.1
Candidatus_Odyssella_thessalonicensis_WP_010298363.1
Candidatus_Paracaedibacter_acanthamoebae_WP_038463764.1
Wolbachia_endosymbiont_of_Drosophila_simulans_WP_015587892.1
Wolbachia_pipientis_WP_070065306.1
Wolbachia_endosymbiont_of_Brugia_malayi_WP_011256791.1
Wolbachia_endosymbiont_of_Onchocerca_volvulus_WP_025263946.1
Candidatus_Neoehrlichia_lotoris_WP_045808838.1
Anaplasma_phagocytophilum_WP_070258520.1
Anaplasma_marginale_WP_012659034.1
Anaplasma_centrale_WP_012880564.1
Ehrlichia_ruminantium_GAT78583.1
Ehrlichia_canis_WP_011304689.1
Ehrlichia_chaffeensis_WP_044193414.1
Ehrlichia_muris_WP_024072199.1
Ehrlichia_sp._HF_WP_044194478.1
Francisella_tularensis_WP_003015439.1
Betaproteobacteria_bacterium_CG2_30_59_46_OIP07010.1
Moraxella_osloensis_WP_065252573.1
Colwellia_aestuarii_WP_077287175.1
Vibrio_jasicida_WP_038869564.1
Aeromonas_lacus_WP_033114485.1
Enterobacter_cloacae_subsp._cloacae_NCTC_9394_CBK85901.1
Morganella_morganii_WP_054423717.1
Photorhabdus_asymbiotica_WP_036772476.1
Xenorhabdus_eapokensis_WP_074022842.1
Asanoa_ferruginea_WP_024827913.1
Pantholops_hodgsonii_XP_005964024.1
Probable contaminant of eukaryotic sequence
Proteobacteria_bacterium_CAG_495_CCZ31195.1
uncultured_bacterium_EIL102C09_ALS55984.1
Alphaproteobacteria_bacterium_GWF2_58_20_OFW84274.1
Rhodospirillum_rubrum_WP_011388033.1
Magnetospirillum_magneticum_WP_011383042.1
Oceanibaculum_indicum_WP_008944252.1
Rhodobacter_vinaykumarii_WP_076362921.1
Litoreibacter_arenae_WP_021099868.1
Ruegeria_lacuscaerulensis_WP_040634782.1
Phaeobacter_gallaeciensis_WP_065273803.1
Halocynthiibacter_namhaensis_WP_039019486.1
Loktanella_hongkongensis_WP_026147456.1
Candidatus_Jidaibacter_acanthamoeba_WP_039456596.1
endosymbiont_of_Acanthamoeba_sp._UWC8_AIF80655.1
Rickettsiales_bacterium_Ac37b_WP_038603034.1
Candidatus_Midichloria_mitochondrii_WP_013950716.1
Rickettsia_bellii_WP_012151566.1
Rickettsia_akari_WP_012149987.1
Rickettsia_typhi_WP_011191143.1
Rickettsia_prowazekii_WP_010886359.1
Rickettsia_tamurae_WP_032138965.1
Rickettsia_conorii_WP_016926529.1
Rickettsia_massiliae_WP_012153106.1
Rickettsia_helvetica_WP_010421444.1
Rickettsia_felis_WP_011270543.1
Rickettsia_canadensis_WP_014364177.1
Branches increased horizontally by 300%
0.1 sub./site

FIG. S8

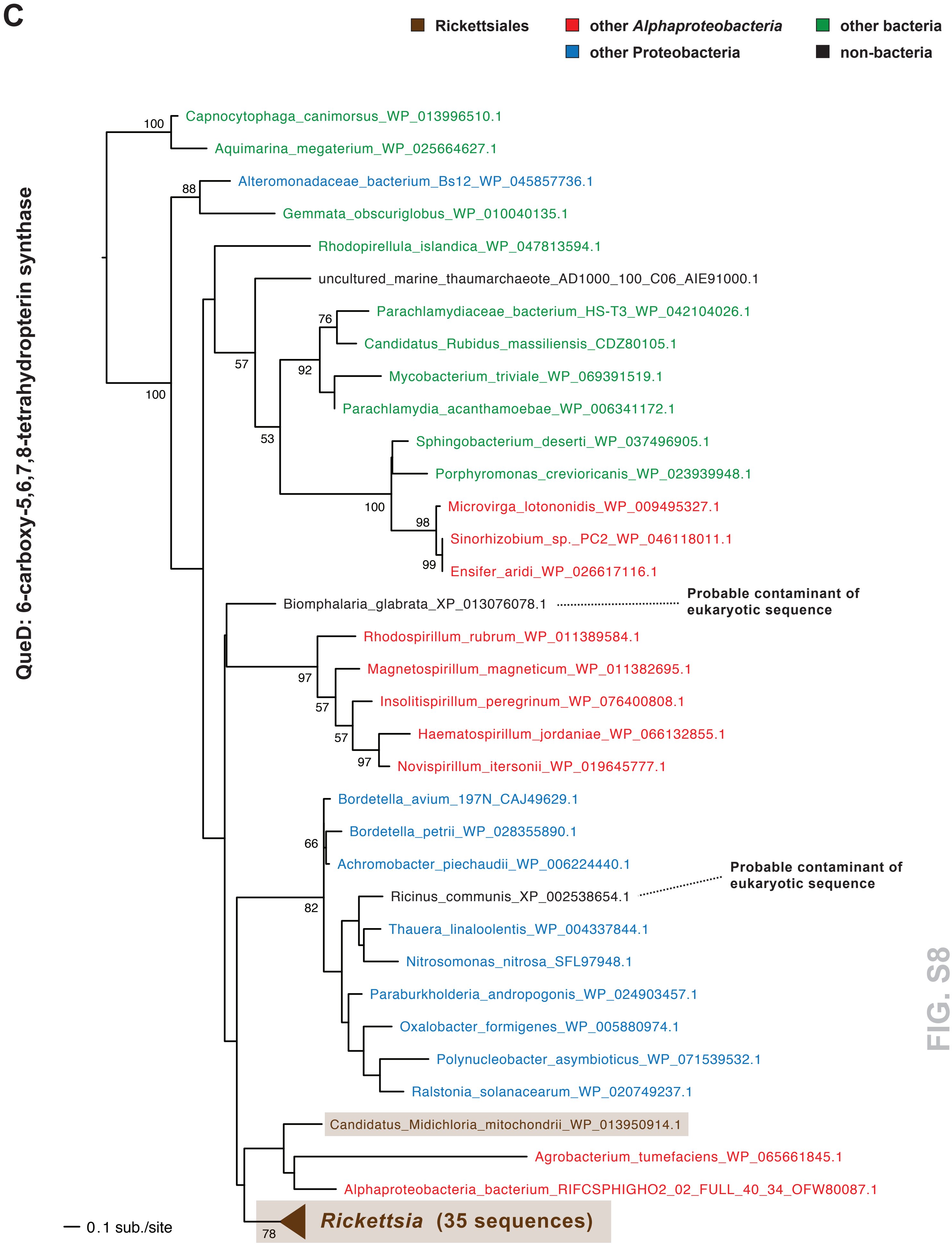
C
Rickettsiales
other Alphaproteobacteria
other bacteria
other Proteobacteria
non-bacteria
QueD: 6-carboxy-5,6,7,8-tetrahydropterin synthase
Capnocytophaga_canimorsus_WP_013996510.1
Aquimarina_megaterium_WP_025664627.1
Alteromonadaceae_bacterium_Bs12_WP_045857736.1
Gemmata_obscuriglobus_WP_010040135.1
Rhodopirellula_islandica_WP_047813594.1
uncultured_marine_thaumarchaeote_AD1000_100_C06_AIE91000.1
Parachlamydiaceae_bacterium_HS-T3_WP_042104026.1
Candidatus_Rubidus_massiliensis_CDZ80105.1
Mycobacterium_triviale_WP_069391519.1
Parachlamydia_acanthamoebae_WP_006341172.1
Sphingobacterium_deserti_WP_037496905.1
Porphyromonas_crevioricanis_WP_023939948.1
Microvirga_lotononidis_WP_009495327.1
Sinorhizobium_sp._PC2_WP_046118011.1
Ensifer_aridi_WP_026617116.1
Biomphalaria_glabrata_XP_013076078.1
Probable contaminant of eukaryotic sequence
Rhodospirillum_rubrum_WP_011389584.1
Magnetospirillum_magneticum_WP_011382695.1
Insolitispirillum_peregrinum_WP_076400808.1
Haematospirillum_jordaniae_WP_066132855.1
Novispirillum_itersonii_WP_019645777.1
Bordetella_avium_197N_CAJ49629.1
Bordetella_petrii_WP_028355890.1
Achromobacter_piechaudii_WP_006224440.1
Ricinus_communis_XP_002538654.1
Probable contaminant of eukaryotic sequence
Thauera_linaloolentis_WP_004337844.1
Nitrosomonas_nitrosa_SFL97948.1
Paraburkholderia_andropogonis_WP_024903457.1
Oxalobacter_formigenes_WP_005880974.1
Polynucleobacter_asymbioticus_WP_071539532.1
Ralstonia_solanacearum_WP_020749237.1
Candidatus_Midichloria_mitochondrii_WP_013950914.1
Agrobacterium_tumefaciens_WP_065661845.1
Alphaproteobacteria_bacterium_RIFCSPHIGHO2_02_FULL_40_34_OFW80087.1
Rickettsia (35 sequences)
0.1 sub./site
100
88
100
57
76
92
53
100
98
99
97
57
57
97
66
82
78

FIG. S8

D

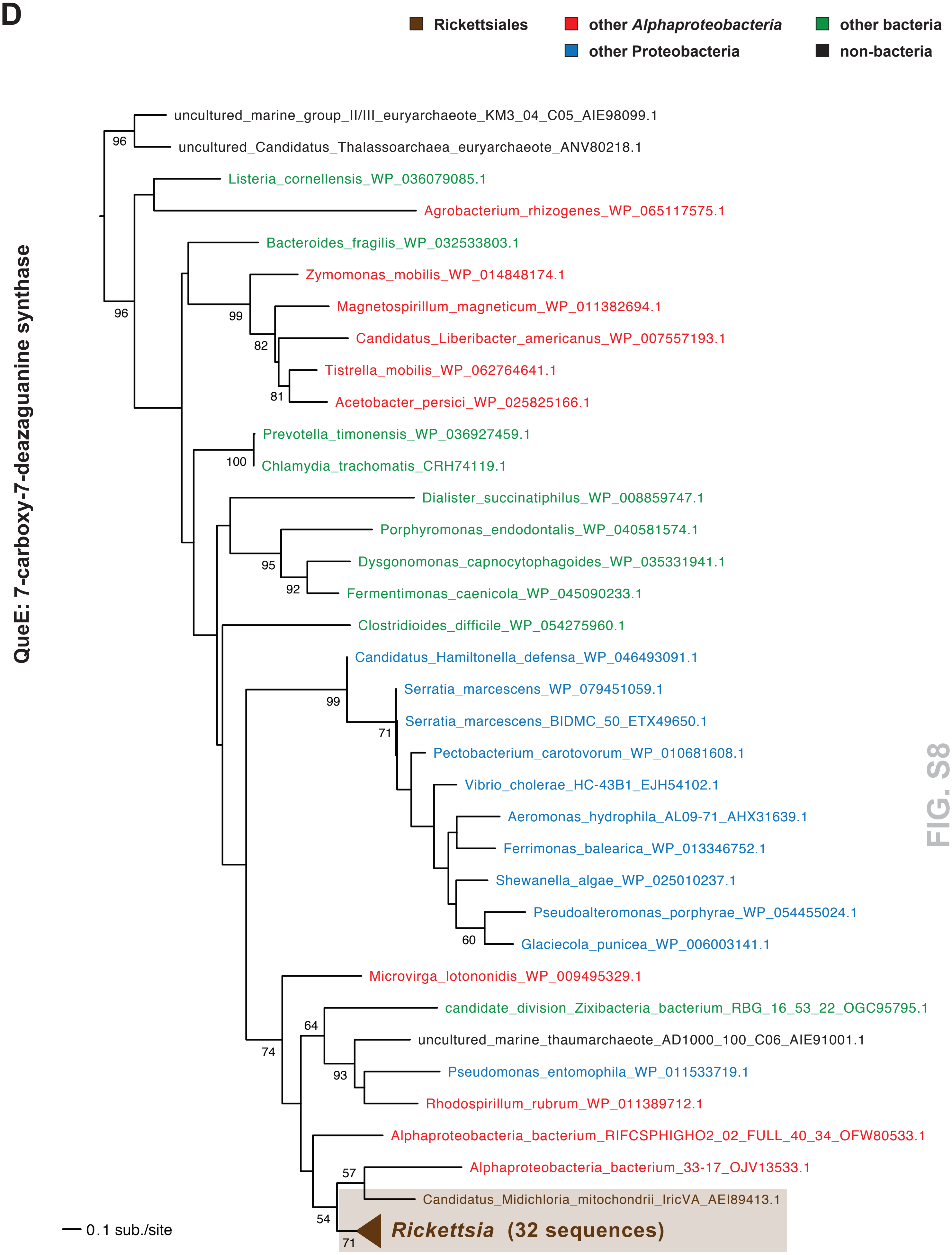
Rickettsiales
other Alphaproteobacteria
other bacteria
other Proteobacteria
non-bacteria
QueE: 7-carboxy-7-deazaguanine synthase
uncultured_marine_group_II/III_euryarchaeote_KM3_04_C05_AIE98099.1
uncultured_Candidatus_Thalassoarchaea_euryarchaeote_ANV80218.1
Listeria_cornellensis_WP_036079085.1
Agrobacterium_rhizogenes_WP_065117575.1
Bacteroides_fragilis_WP_032533803.1
Zymomonas_mobilis_WP_014848174.1
Magnetospirillum_magneticum_WP_011382694.1
Candidatus_Liberibacter_americanus_WP_007557193.1
Tistrella_mobilis_WP_062764641.1
Acetobacter_persici_WP_025825166.1
Prevotella_timonensis_WP_036927459.1
Chlamydia_trachomatis_CRH74119.1
Dialister_succinatiphilus_WP_008859747.1
Porphyromonas_endodontalis_WP_040581574.1
Dysgonomonas_capnocytophagoides_WP_035331941.1
Fermentimonas_caenicola_WP_045090233.1
Clostridioides_difficile_WP_054275960.1
Candidatus_Hamiltonella_defensa_WP_046493091.1
Serratia_marcescens_WP_079451059.1
Serratia_marcescens_BIDMC_50_ETX49650.1
Pectobacterium_carotovorum_WP_010681608.1
Vibrio_cholerae_HC-43B1_EJH54102.1
Aeromonas_hydrophila_AL09-71_AHX31639.1
Ferrimonas_balearica_WP_013346752.1
Shewanella_algae_WP_025010237.1
Pseudoalteromonas_porphyrae_WP_054455024.1
Glaciecola_punicea_WP_006003141.1
Microvirga_lotononidis_WP_009495329.1
candidate_division_Zixibacteria_bacterium_RBG_16_53_22_OGC95795.1
uncultured_marine_thaumarchaeote_AD1000_100_C06_AIE91001.1
Pseudomonas_entomophila_WP_011533719.1
Rhodospirillum_rubrum_WP_011389712.1
Alphaproteobacteria_bacterium_RIFCSPHIGHO2_02_FULL_40_34_OFW80533.1
Alphaproteobacteria_bacterium_33-17_OJV13533.1
Candidatus_Midichloria_mitochondrii_IricVA_AEI89413.1
Rickettsia (32 sequences)
96
96
99
82
81
100
95
92
99
71
60
74
64
93
57
54
71
0.1 sub./site

FIG. S8

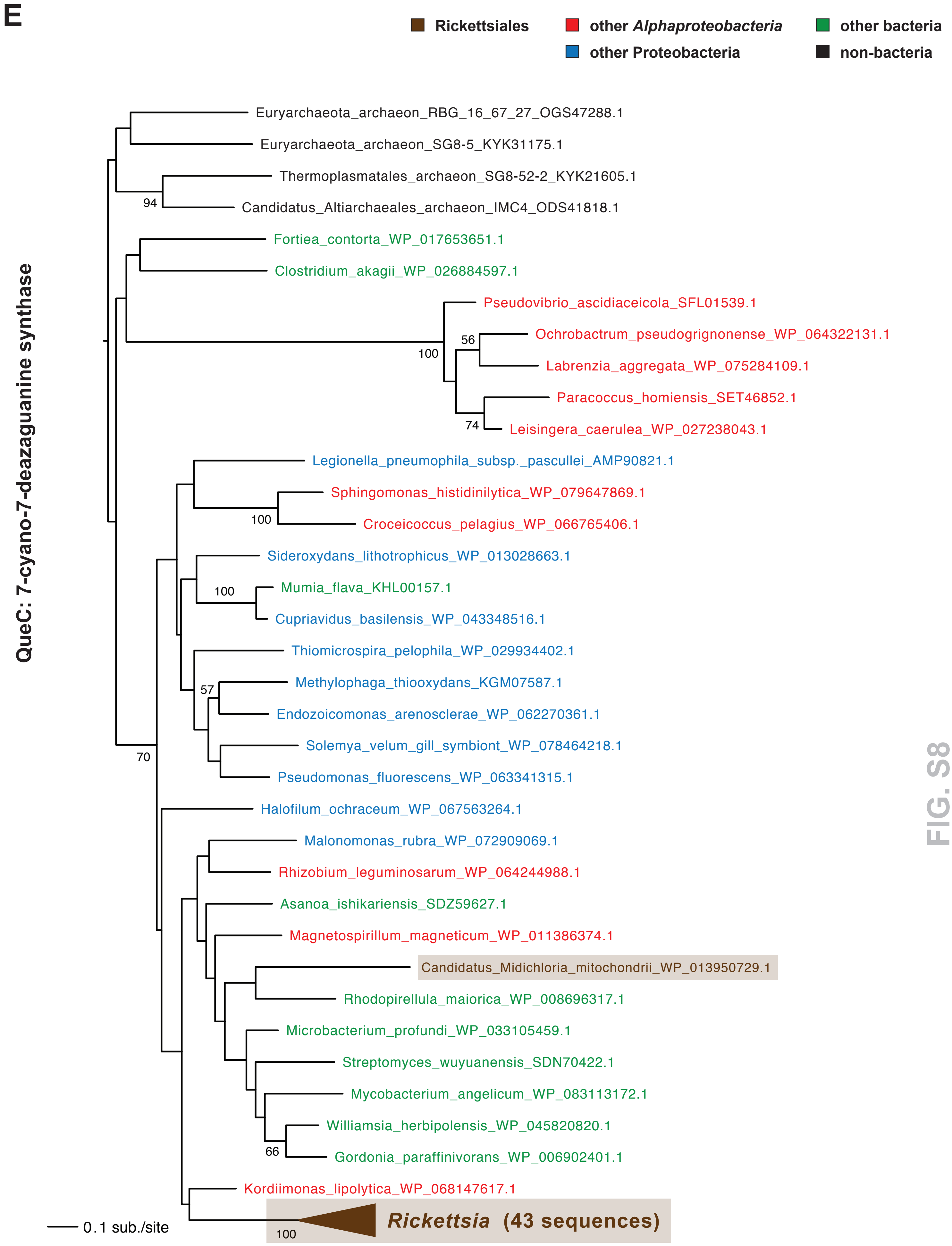
E
Rickettsiales
other Alphaproteobacteria
other bacteria
other Proteobacteria
non-bacteria
QueC: 7-cyano-7-deazaguanine synthase
Euryarchaeota_archaeon_RBG_16_67_27_OGS47288.1
Euryarchaeota_archaeon_SG8-5_KYK31175.1
Thermoplasmatales_archaeon_SG8-52-2_KYK21605.1
94
Candidatus_Altiarchaeales_archaeon_IMC4_ODS41818.1
Fortiea_contorta_WP_017653651.1
Clostridium_akagii_WP_026884597.1
Pseudovibrio_ascidiaceicola_SFL01539.1
56
Ochrobactrum_pseudogrignonense_WP_064322131.1
100
Labrenzia_aggregata_WP_075284109.1
Paracoccus_homiensis_SET46852.1
74
Leisingera_caerulea_WP_027238043.1
Legionella_pneumophila_subsp._pascullei_AMP90821.1
Sphingomonas_histidinilytica_WP_079647869.1
100
Croceicoccus_pelagius_WP_066765406.1
Sideroxydans_lithotrophicus_WP_013028663.1
100
Mumia_flava_KHL00157.1
Cupriavidus_basilensis_WP_043348516.1
Thiomicrospira_pelophila_WP_029934402.1
57
Methylophaga_thiooxydans_KGM07587.1
Endozoicomonas_arenosclerae_WP_062270361.1
Solemya_velum_gill_symbiont_WP_078464218.1
70
Pseudomonas_fluorescens_WP_063341315.1
Halofilum_ochraceum_WP_067563264.1
Malonomonas_rubra_WP_072909069.1
Rhizobium_leguminosarum_WP_064244988.1
Asanoa_ishikariensis_SDZ59627.1
Magnetospirillum_magneticum_WP_011386374.1
Candidatus_Midichloria_mitochondrii_WP_013950729.1
Rhodopirellula_maiorica_WP_008696317.1
Microbacterium_profundi_WP_033105459.1
Streptomyces_wuyuanensis_SDN70422.1
Mycobacterium_angelicum_WP_083113172.1
Williamsia_herbipolensis_WP_045820820.1
66
Gordonia_paraffinivorans_WP_006902401.1
Kordiimonas_lipolytica_WP_068147617.1
0.1 sub./site
100
Rickettsia (43 sequences)

FIG. S8

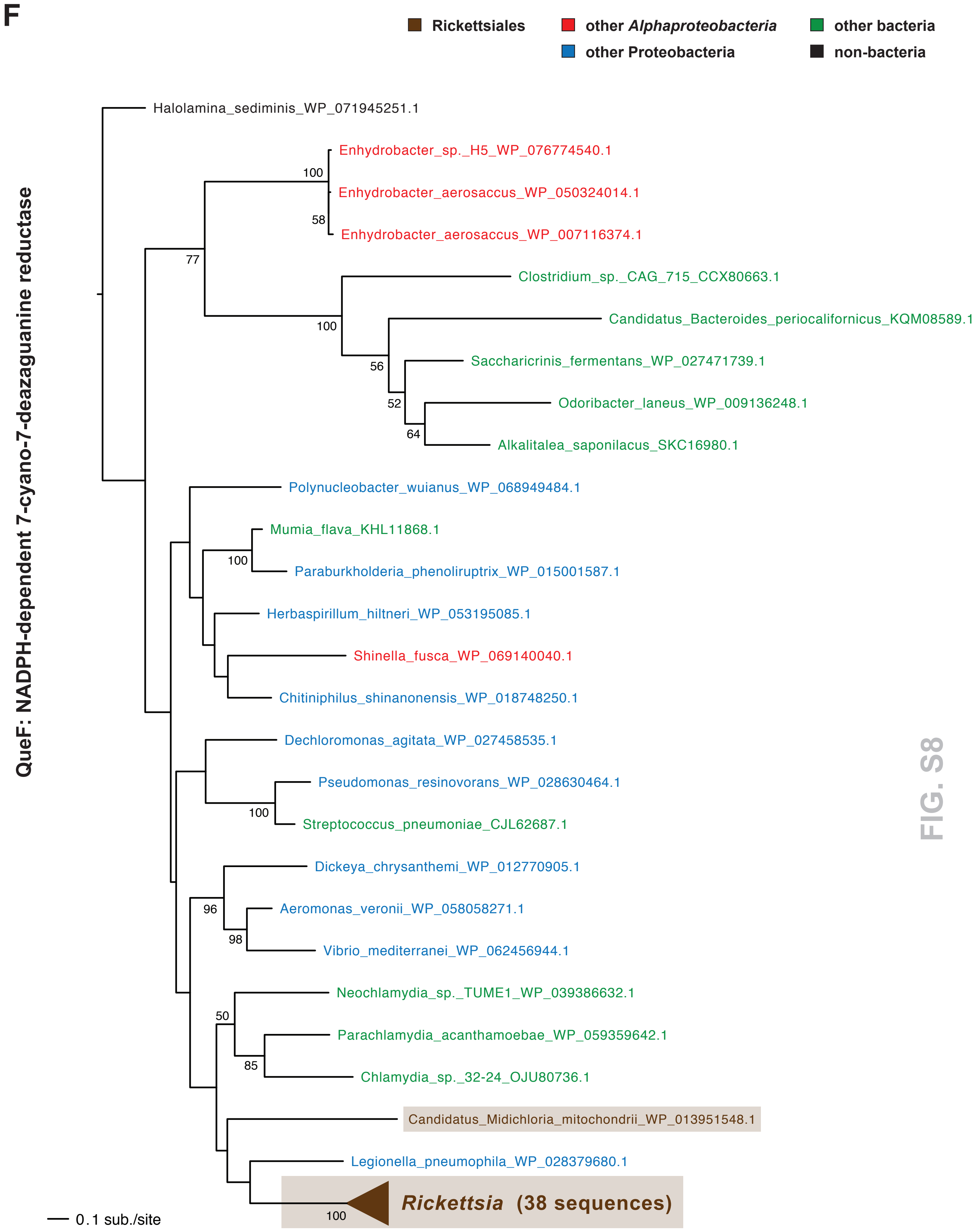
F
Rickettsiales
other Alphaproteobacteria
other bacteria
other Proteobacteria
non-bacteria
QueF: NADPH-dependent 7-cyano-7-deazaguanine reductase
Halolamina_sediminis_WP_071945251.1
Enhydrobacter_sp._H5_WP_076774540.1
Enhydrobacter_aerosaccus_WP_050324014.1
Enhydrobacter_aerosaccus_WP_007116374.1
Clostridium_sp._CAG_715_CCX80663.1
Candidatus_Bacteroides_periocalifornicus_KQM08589.1
Saccharicrinis_fermentans_WP_027471739.1
Odoribacter_laneus_WP_009136248.1
Alkalitalea_saponilacus_SKC16980.1
Polynucleobacter_wuianus_WP_068949484.1
Mumia_flava_KHL11868.1
Paraburkholderia_phenoliruptrix_WP_015001587.1
Herbaspirillum_hiltneri_WP_053195085.1
Shinella_fusca_WP_069140040.1
Chitiniphilus_shinanonensis_WP_018748250.1
Dechloromonas_agitata_WP_027458535.1
Pseudomonas_resinovorans_WP_028630464.1
Streptococcus_pneumoniae_CJL62687.1
Dickeya_chrysanthemi_WP_012770905.1
Aeromonas_veronii_WP_058058271.1
Vibrio_mediterranei_WP_062456944.1
Neochlamydia_sp._TUME1_WP_039386632.1
Parachlamydia_acanthamoebae_WP_059359642.1
Chlamydia_sp._32-24_OJU80736.1
Candidatus_Midichloria_mitochondrii_WP_013951548.1
Legionella_pneumophila_WP_028379680.1
Rickettsia (38 sequences)
100
58
77
100
56
52
64
100
100
96
98
50
85
100
0.1 sub./site

FIG. S8

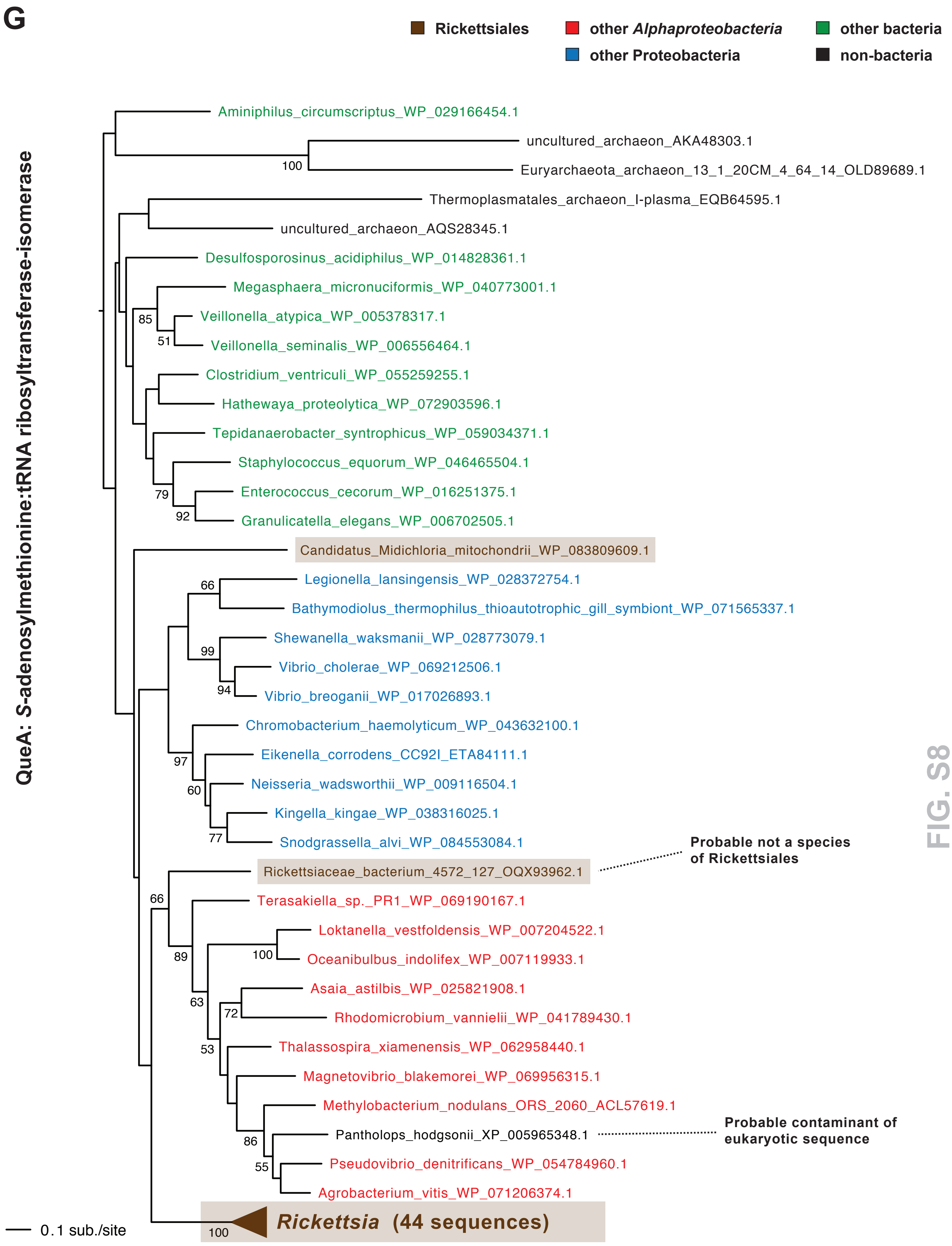
G
Rickettsiales
other Alphaproteobacteria
other bacteria
other Proteobacteria
non-bacteria
QueA: S-adenosylmethionine:tRNA ribosyltransferase-isomerase
Aminiphilus_circumscriptus_WP_029166454.1
uncultured_archaeon_AKA48303.1
Euryarchaeota_archaeon_13_1_20CM_4_64_14_OLD89689.1
Thermoplasmatales_archaeon_I-plasma_EQB64595.1
uncultured_archaeon_AQS28345.1
Desulfosporosinus_acidiphilus_WP_014828361.1
Megasphaera_micronuciformis_WP_040773001.1
Veillonella_atypica_WP_005378317.1
Veillonella_seminalis_WP_006556464.1
Clostridium_ventriculi_WP_055259255.1
Hathewaya_proteolytica_WP_072903596.1
Tepidanaerobacter_syntrophicus_WP_059034371.1
Staphylococcus_equorum_WP_046465504.1
Enterococcus_cecorum_WP_016251375.1
Granulicatella_elegans_WP_006702505.1
Candidatus_Midichloria_mitochondrii_WP_083809609.1
Legionella_lansingensis_WP_028372754.1
Bathymodiolus_thermophilus_thioautotrophic_gill_symbiont_WP_071565337.1
Shewanella_waksmanii_WP_028773079.1
Vibrio_cholerae_WP_069212506.1
Vibrio_breoganii_WP_017026893.1
Chromobacterium_haemolyticum_WP_043632100.1
Eikenella_corrodens_CC92I_ETA84111.1
Neisseria_wadsworthii_WP_009116504.1
Kingella_kingae_WP_038316025.1
Snodgrassella_alvi_WP_084553084.1
Rickettsiaceae_bacterium_4572_127_OQX93962.1
Probable not a species of Rickettsiales
Terasakiella_sp._PR1_WP_069190167.1
Loktanella_vestfoldensis_WP_007204522.1
Oceanibulbus_indolifex_WP_007119933.1
Asaia_astilbis_WP_025821908.1
Rhodomicrobium_vannielii_WP_041789430.1
Thalassospira_xiamenensis_WP_062958440.1
Magnetovibrio_blakemorei_WP_069956315.1
Methylobacterium_nodulans_ORS_2060_ACL57619.1
Pantholops_hodgsonii_XP_005965348.1
Probable contaminant of eukaryotic sequence
Pseudovibrio_denitrificans_WP_054784960.1
Agrobacterium_vitis_WP_071206374.1
Rickettsia (44 sequences)
100
100
85
51
79
92
66
99
94
97
60
77
66
89
100
63
72
53
86
55
0.1 sub./site

FIG. S8

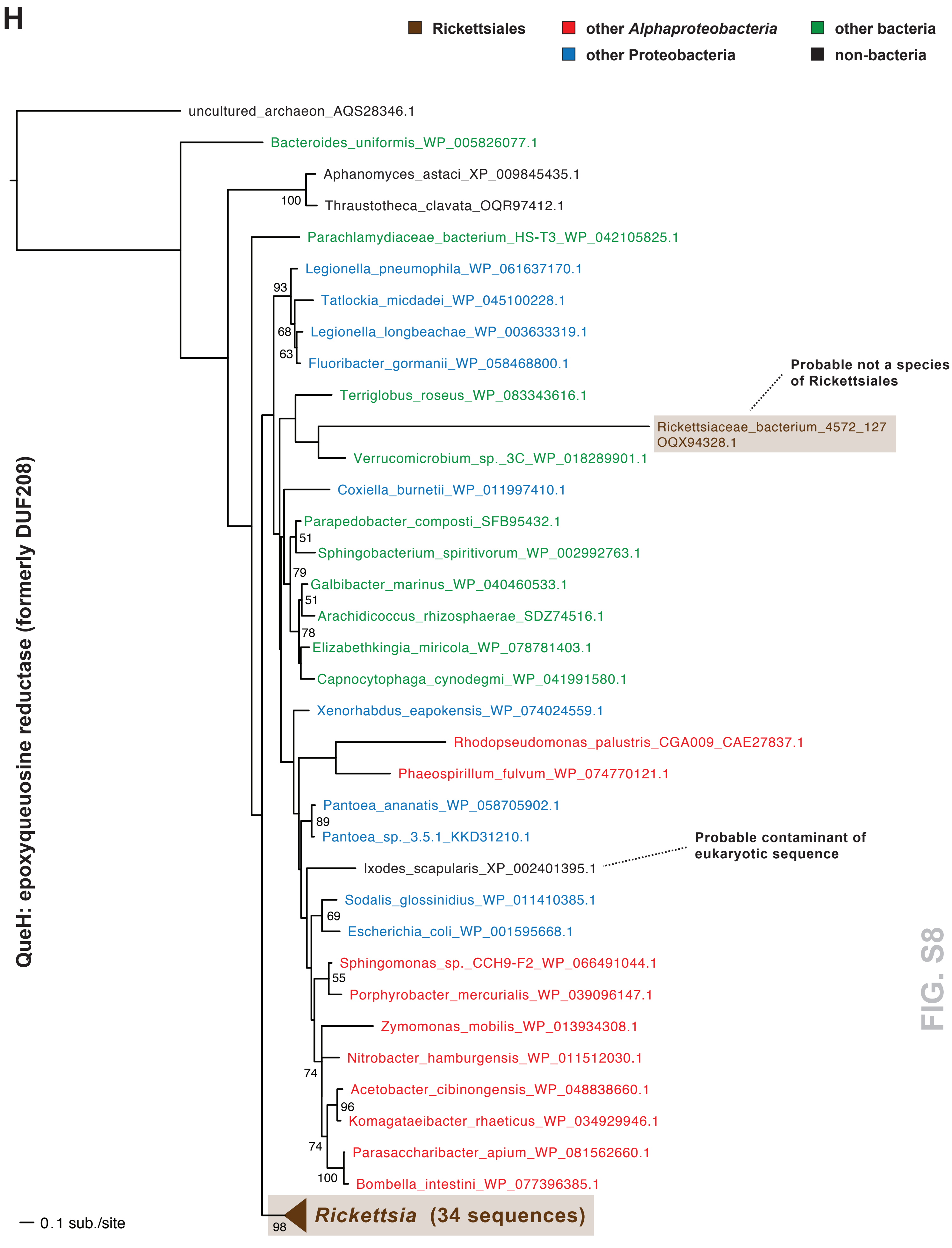
H
Rickettsiales
other Alphaproteobacteria
other bacteria
other Proteobacteria
non-bacteria
QueH: epoxyqueuosine reductase (formerly DUF208)
uncultured_archaeon_AQS28346.1
Bacteroides_uniformis_WP_005826077.1
Aphanomyces_astaci_XP_009845435.1
100
Thraustotheca_clavata_OQR97412.1
Parachlamydiaceae_bacterium_HS-T3_WP_042105825.1
Legionella_pneumophila_WP_061637170.1
93
Tatlockia_micdadei_WP_045100228.1
68
Legionella_longbeachae_WP_003633319.1
63
Fluoribacter_gormanii_WP_058468800.1
Probable not a species of Rickettsiales
Terriglobus_roseus_WP_083343616.1
Rickettsiaceae_bacterium_4572_127 OQX94328.1
Verrucomicrobium_sp._3C_WP_018289901.1
Coxiella_burnetii_WP_011997410.1
Parapedobacter_composti_SFB95432.1
51
Sphingobacterium_spiritivorum_WP_002992763.1
79
Galbibacter_marinus_WP_040460533.1
51
Arachidicoccus_rhizosphaerae_SDZ74516.1
78
Elizabethkingia_miricola_WP_078781403.1
Capnocytophaga_cynodegmi_WP_041991580.1
Xenorhabdus_eapokensis_WP_074024559.1
Rhodopseudomonas_palustris_CGA009_CAE27837.1
Phaeospirillum_fulvum_WP_074770121.1
Pantoea_ananatis_WP_058705902.1
89
Pantoea_sp._3.5.1_KKD31210.1
Probable contaminant of eukaryotic sequence
Ixodes_scapularis_XP_002401395.1
Sodalis_glossinidius_WP_011410385.1
69
Escherichia_coli_WP_001595668.1
Sphingomonas_sp._CCH9-F2_WP_066491044.1
55
Porphyrobacter_mercurialis_WP_039096147.1
Zymomonas_mobilis_WP_013934308.1
Nitrobacter_hamburgensis_WP_011512030.1
74
Acetobacter_cibinongensis_WP_048838660.1
96
Komagataeibacter_rhaeticus_WP_034929946.1
74
Parasaccharibacter_apium_WP_081562660.1
100
Bombella_intestini_WP_077396385.1
Rickettsia (34 sequences)
98
0.1 sub./site

FIG. S8
