# Supplementary material for: Wholly Rickettsia! Reconstructed Metabolic Profile of the Quintessential Bacterial Parasite of Eukaryotic Cells
Source: mBio. 2017 Sep 26;8(5):e00859-17. doi: 10.1128/mBio.00859-17 (PMC5615194; doi:10.1128/mBio.00859-17)
Supplement: FIG S9 [file mbo005173495sf9.pdf]

FIG S9. *Rickettsia* transport and metabolism of host-acquired ribonucletoides.

(A) Current knowledge of *Rickettsia* ribonucleotide transport. The five Tlc ribonucleotide transporters are shown as previously characterized (1, 2). The substrates of Tlc2 and Tlc3, as well as the transport mechanism(s) for UMP, AMP and GMP (3–5), remain unknown.

(B) Rickettsiae acquire host UMP, UTP and CTP to drive the synthesis of additional pyrimidine ribonucleotides, as well as all pyrimidine deoxyribonucleotides.

(C) Host-acquired guanosine (GMP, GDP and GTP) and adenosine (AMP and ATP) ribonucleotides independently drive the synthesis of guanosine and adenosine deoxyribonucleotides. Regarding GTP metabolism, rickettsiae encode stringent response regulatory proteins, including SpoT hydrolases and synthetases (highly variable in number across rickettsial species), as well as the guanosine-5'-triphosphate,3'-diphosphate pyrophosphatase GppA (highly conserved across *Rickettsia* genomes) (6). Rickettsiae also contain deoxyguanosinetriphosphate triphosphohydrolase (Dgt), which hydrolyzes dGTP to form deoxyguanosine, the functional significance of which is not clear. Finally, rickettsiae harbor SAICAR synthetase (PurC), which typically functions in both purine and thiamine metabolism. As these pathways are disintegrated in rickettsiae, PurC is an orphan enzyme with unknown functional significance. CAIR, 1-(5-Phospho-D-ribosyl)-5-amino-4-imidazolecarboxylate; SAICAR, 1-(5'-Phosphoribosyl)-5-amino-4-(N-succinocarboxamide)-imidazole.

REFERENCES

1. **Winkler HH**. 1976. Rickettsial permeability. An ADP-ATP transport system. J Biol Chem **251**:389–396.
2. **Audia JP**, **Winkler HH**. 2006. Study of the five Rickettsia prowazekii proteins annotated as ATP/ADP translocases (Tlc): Only Tlc1 transports ATP/ADP, while Tlc4 and Tlc5 transport other ribonucleotides. J Bacteriol **188**:6261–6268.
3. **Winkler HH**, **Daugherty R**, **Hu F**. 1999. Rickettsia prowazekii transports ump and gmp,

but not cmp, as building blocks for rna synthesis. J Bacteriol **181**:3238–3241.

4. **Atkinson WH**, **Winkler HH**. 1985. Transport of AMP by Rickettsia prowazekii. J Bacteriol **161**:32–38.

5. **Speed RR**, **Winkler HH**. 1991. Acquisition of thymidylate by the obligate intracytoplasmic bacterium Rickettsia prowazekii. J Bacteriol **173**:1704–10.

6. **Gillespie JJ**, **Joardar V**, **Williams KP**, **Driscoll T**, **Hostetler JB**, **Nordberg E**, **Shukla M**, **Walenz B**, **Hill CA**, **Nene VM**, **Azad AF**, **Sobral BW**, **Caler E**. 2012. A Rickettsia genome overrun by mobile genetic elements provides insight into the acquisition of genes characteristic of an obligate intracellular lifestyle. J Bacteriol **194**:376–94.

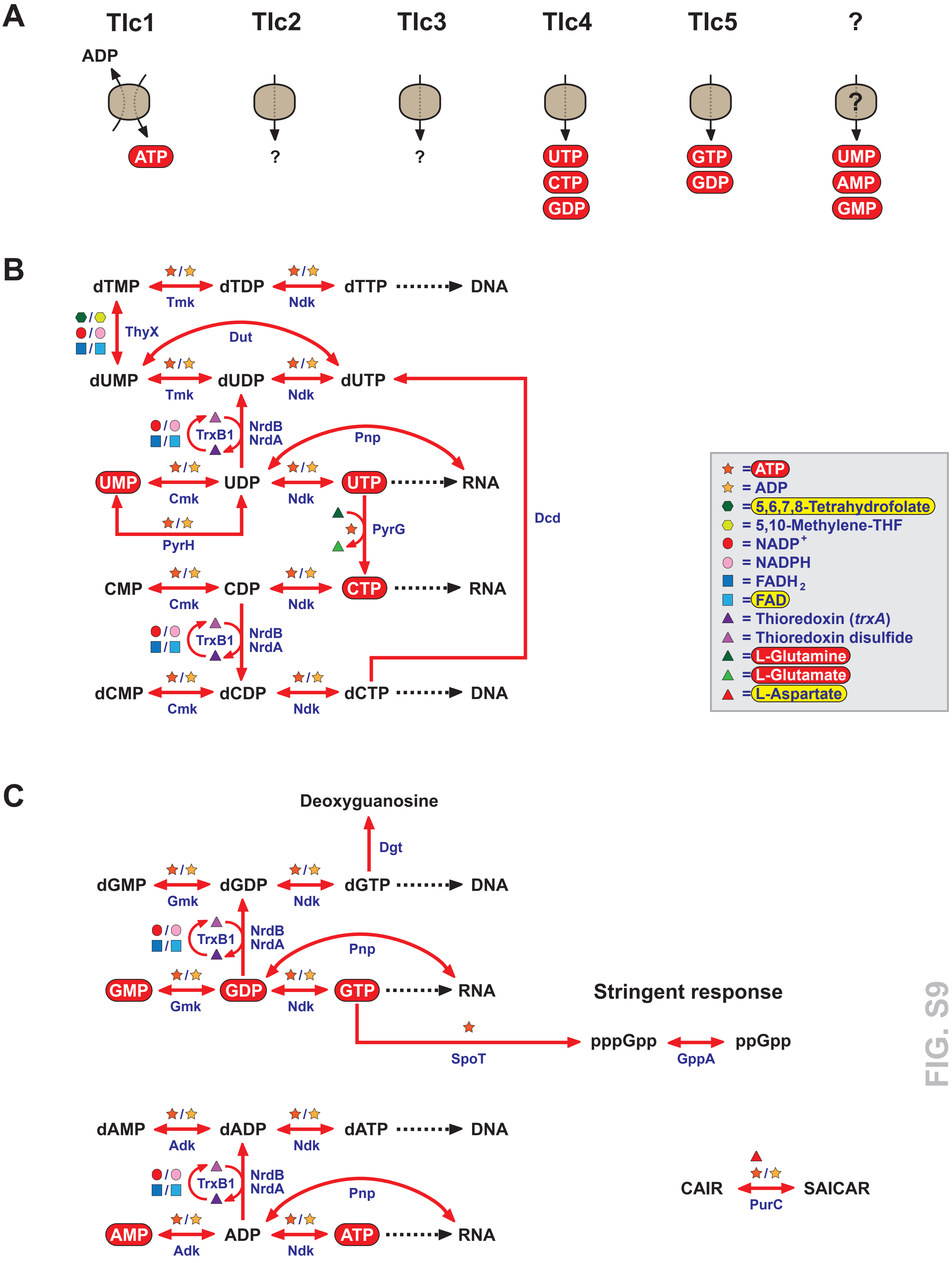

A
Tlc1
Tlc2
Tlc3
Tlc4
Tlc5
?
ADP
ATP
UTP
CTP
GDP
GTP
GDP
UMP
AMP
GMP
B
dTMP
dTDP
dTTP
DNA
Tmk
Ndk
ThyX
Dut
dUMP
dUDP
dUTP
TrxB1
NrdB
NrdA
Pnp
UMP
UDP
UTP
RNA
Cmk
PyrH
PyrG
Dcd
CMP
CDP
CTP
dCMP
dCDP
dCTP
= ATP
= ADP
= 5,6,7,8-Tetrahydrofolate
= 5,10-Methylene-THF
= NADP+
= NADPH
= FADH2
= FAD
= Thioredoxin (trxA)
= Thioredoxin disulfide
= L-Glutamine
= L-Glutamate
= L-Aspartate
C
Deoxyguanosine
Dgt
dGMP
dGDP
dGTP
Gmk
GMP
GDP
GTP
Stringent response
pppGpp
ppGpp
SpoT
GppA
dAMP
dADP
dATP
Adk
AMP
ADP
ATP
CAIR
SAICAR
PurC

FIG. S9
